# Enhanced Seed Viability Correlates with Husk Adhesion in Barley (*Hordeum vulgare* L.)

**DOI:** 10.3390/plants15192976

**Published:** 2026-09-29

**Authors:** Smitha Chundakkad, Workie Zegeye, Paola Higuera-Poveda, Saranya Cherukudi-Mattuvayal, Eleni Vikeli, Ajay Siluveru, Pauline Stephenson, Richard Horler, Simon Orford, Liz Sayers, Noam Chayut

**Affiliations:** Germplasm Resources Unit, John Innes Centre, Norwich Research Park, Colney Lane, Norwich NR4 7UH, UK; smitha.chundakkad@jic.ac.uk (S.C.); workie.zegeye@jic.ac.uk (W.Z.);

**Keywords:** seed longevity, ex situ conservation, genebank, phenotyping, grain protein content, barley (*Hordeum vulgare* L.)

## Abstract

Understanding seed longevity in genebanks is fundamental for effective long-term conservation of plant genetic resources. Although storage environment, species biology, seed age, and oxygen exposure are known to influence viability, substantial variation in germination among accessions stored under identical conditions suggests the presence of additional genetic determinants. Here, we analysed 947 barley seed lots (686 hulled and 261 hulless) across nine harvest years (1975–2017) to assess the relationship between seed viability and a broad range of phenotypic traits, including plant phenology, inflorescence and seed morphology, and seed macronutrient composition. Each harvest year represented a distinct set of genebank accessions cultivated under uniform field conditions and stored for equal durations. Hulled barley consistently exhibited higher viability than hulless barley, and grain protein content was negatively associated with germination in five of the seven years studied. Additionally, year-specific but statistically significant associations were detected for ear emergence timing and lemma pigmentation. These results underscore the value of comprehensive genebank descriptors and phenotypic metadata for improving predictions of seed longevity and supporting strategic germplasm conservation.

## 1. Introduction

Conservation of crop genetic diversity is essential for sustaining a growing global population and mitigating the impacts of climate change on food production. Germplasm diversity provides the foundation for crop improvement, enabling allele discovery, trait utilisation, and long-term breeding innovation. To safeguard this genetic variation and prevent the loss of potentially valuable alleles, approximately 1750 ex situ genebanks have been established worldwide over the past half-century. Together, they conserve more than 7.4 million plant accessions, nearly 45% of which are cereals [1,2]. Genebanks curate germplasm and its associated information in accessible formats and play a crucial role in enabling use by scientists, breeders, and other stakeholders. A clear understanding of seed longevity under ex situ conditions is therefore fundamental to effective genebank operation and management [3].

Although tailored storage conditions substantially prolong seed longevity, deterioration cannot be entirely prevented. Ageing seed lots ultimately require regeneration or re-collection [1]. During regeneration, crop descriptors are routinely used to characterise plants and seeds throughout growth, harvest, and post-harvest stages. These descriptors consist of distinctive, heritable traits that are simple to score and help verify the accuracy of regeneration events. In the Germplasm Resources Unit at the John Innes Centre (JIC), barley descriptors include plant height, heading date, ear and awn type, grain pigmentation, hullness, and grain size. Additional phenotypes recorded historically at the Plant Breeding Institute (PBI), Cambridge, and subsequently at JIC were digitised into SeedStor (https://www.seedstor.ac.uk, accessed on 1 June 2026), a germplasm information management system [4], providing over 200,000 phenotypic data points for approximately 9000 barley accessions. These searchable datasets enable users to select germplasm based on defined traits and allow custodians to verify new regenerations against previous records.

Accurate prediction of seed longevity is central to the sustainable and cost-effective management of ex situ collections [5]. Recent reviews summarise advances in understanding the biological and environmental determinants of seed ageing and their implications for predicting seed longevity [6]. While longevity typically varies more between species than within species [7], genetic variation influencing viability has been identified in several crop and model species, including wheat [2,8], tomato [9], bell pepper [10], rice [11], and *Arabidopsis* [12,13]. Key environmental drivers of viability loss include seed age, storage temperature and relative humidity (RH), fluctuations in these conditions, oxygen damage, and the initial seed quality [6]. In addition, the maternal environment during seed development may influence subsequent longevity [14].

Barley (*Hordeum vulgare* L.) is the fourth most important cereal crop globally, widely cultivated and consumed [15], and a key model species for cereal genetics. Consequently, it is extensively conserved in genebanks, with more than 400,000 accessions preserved worldwide [16,17]. The barley materials investigated in this study derive from the UKRI-BBSRC Small-Grain Cereal Collection. The studied germplasm has been maintained under cool, dry storage conditions throughout its conservation history. Before 1978, seeds were stored in a thick-walled barn that provided relatively stable, cool, dry ambient conditions. From 1978 to 2018, the collection was conserved under controlled conditions at 1.5 °C (±2) and 8–10% (±2) RH. In 2018, storage conditions were adjusted to 4 °C (±2) and 12% (±2) RH, followed by a further adjustment in 2021 to 5 °C (±2) and 12% (±2) RH. The changes, reflected in genebank management decisions, were applied uniformly across the collection. Seeds are stored in breathable paper envelopes, allowing passive equilibration with the controlled low-humidity storage environment without additional pre-storage drying. Regeneration was carried out at PBI trial sites in Cambridge, UK, until 1990, after which the collection was transferred to JIC, Norwich, UK, where it has been conserved since.

Routine germination testing typically reports values between ~75% and 100% for high-quality genebank seed lots, as lower-viability stocks are generally replaced [3]. However, in earlier decades of the present collection, germination tests were not performed routinely. Instead, accessions were regenerated when stocks ran low or when seeds reached 20–25 years of age. Importantly for this work, seed that was sampled for regeneration or not prioritised for long-term curation remained stored alongside other accessions under identical conditions. This “discard material”, kept rather than destroyed, provides a rare opportunity to examine long-term viability loss in real-world genebank storage over up to 45 years. Seeds from such harvest years exhibited wide germination variability (20–100%), reflecting genuine viability decay under cool-dry conditions.

In this study, we analysed barley germination data from historic seed lots and linked them with extensive phenotypic datasets to explore associations between seed viability and crop descriptors. We discuss the practical implications of these relationships for advancing ex situ seed conservation planning.

## 2. Results

Across the studied barley collection, 82.2% of accessions (7857) were hulled, and 14.8% (1363) were hulless. Germination percentages of hulled and hulless accessions were compared for the oldest seed lots (41–45 years old; harvested in 1974–1978) and for more recent harvest years (HYs) 2003, 2004, 2016, and 2017. A total of 947 germination tests were conducted, comprising 686 hulled and 261 hulless accessions (Supplementary Table S1).

**Figure 1:**
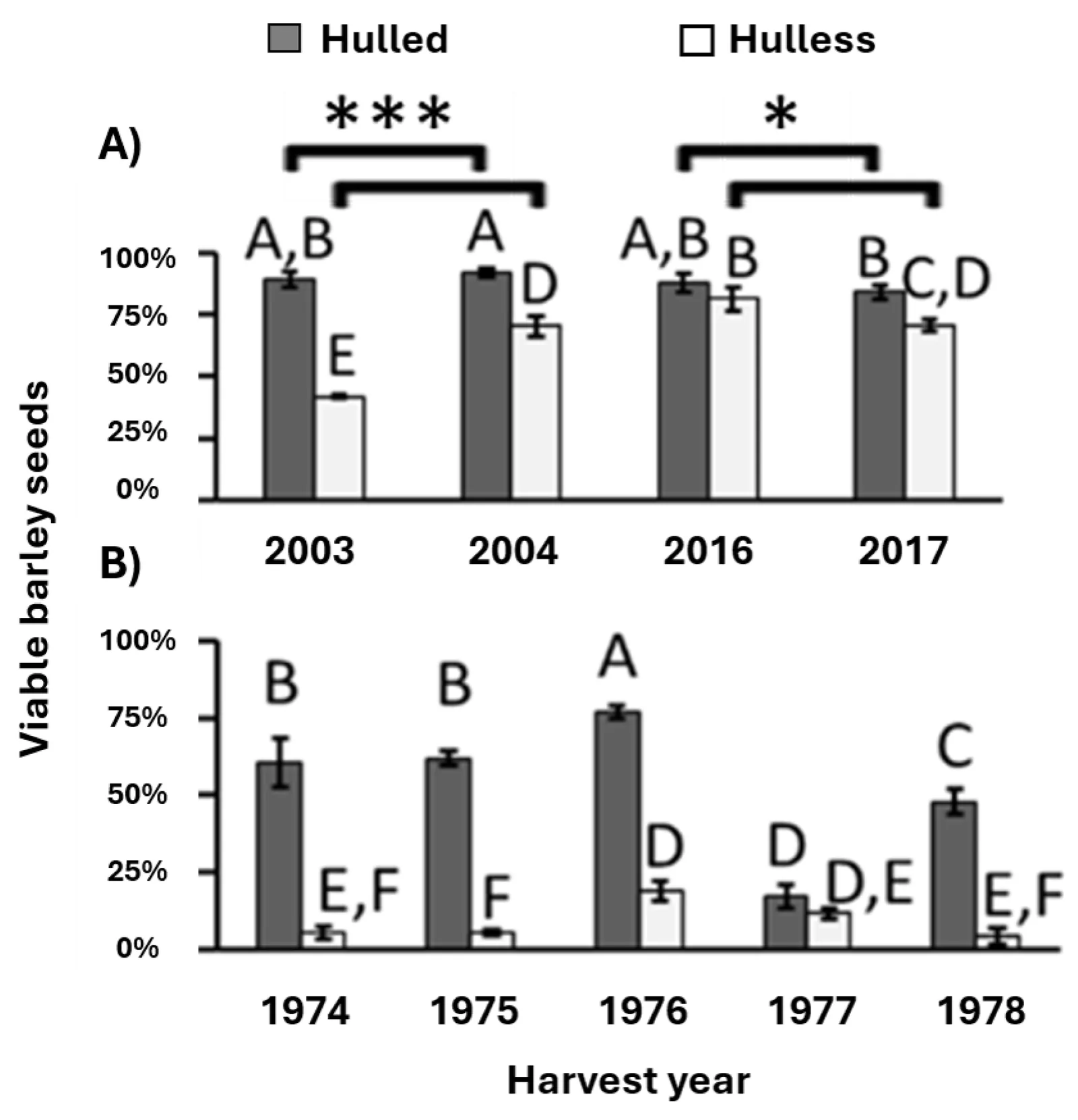
(**A**) Percentage of viable hulled compared to hulless barley seeds harvested in 2003, 2004, 2016, and 1917 (*n* = 250, 350, 250, 220, respectively) at the John Innes Centre field trial station, Norwich, UK. The experiment was conducted in 2020. Bars not marked by similar letters represent statistically different averages (*p* < 0.05; Tukey HSD ANOVA). Linked bars are significantly different (* *p* < 0.05, *** *p* < 0.001). (**B**) Percent of viable hulled compared to hulless barley seeds harvested in 1974, 1975, 1976, 1977, and 1978 (*n* = 24, 100, 100, 100, 60, respectively) in PBI fields, Cambridgeshire, UK. The experiment was conducted in 2019. Bars not marked with the same letters represent statistically different averages (*p* < 0.05, Tukey HSD ANOVA).

Hulled accessions consistently exhibited higher viability across all harvest years (HYs) and exceeded the significance threshold in seven of the nine tested years (Figure 1). Differences were particularly pronounced in the intermediate-aged seed lots. In the 2003 and 2004 HYs, 16–17-year-old hulled seeds remained highly viable (89.4% and 92.0%, respectively), while hulless seeds showed markedly lower viability (42.0% and 70.5%; Pv < 0.001). Similarly, in 3–4-year-old material from HYs 2016 and 2017, hulled seeds averaged 86.1% viability, compared with 79.7% for hulless seeds (Pv = 0.0196). In the oldest seed lots (HYs 1974–1978), hulled accessions averaged 52.8% viability, whereas hulless accessions averaged only 9.2% (Figure 1).

Given the strong effect of hull presence on seed viability and the relatively small number of hulless accessions (261 in total), hulless material was excluded from subsequent analyses to allow identification of more subtle factors influencing seed longevity. Analyses therefore focused on hulled accessions, assessing correlations between germination rate and grain macronutrient composition (protein, starch, and neutral detergent fibre (NDF)), seed morphometrics (grain length, width, and thousand-grain weight (TGW), and additional phenotypic descriptors recorded in SeedStor and described in Section 4.

In total, 990 hulled accessions from seven HYs (1975, 1976, 1979, 2003, 2004, 2016, and 2017) were phenotyped (1975: *n* = 62; 1976: *n* = 359; 1979: *n* = 118; 2003: *n* = 57; 2004: *n* = 280; 2016: *n* = 56; 2017: *n* = 58). All macronutrient and morphometric data are summarised in Supplementary Table S2. HY 1976, grown at the PBI field site (Cambridge, UK), displayed the widest range of germination values (2–100%, mean 62.2 ± 23%).

**Figure 2:**
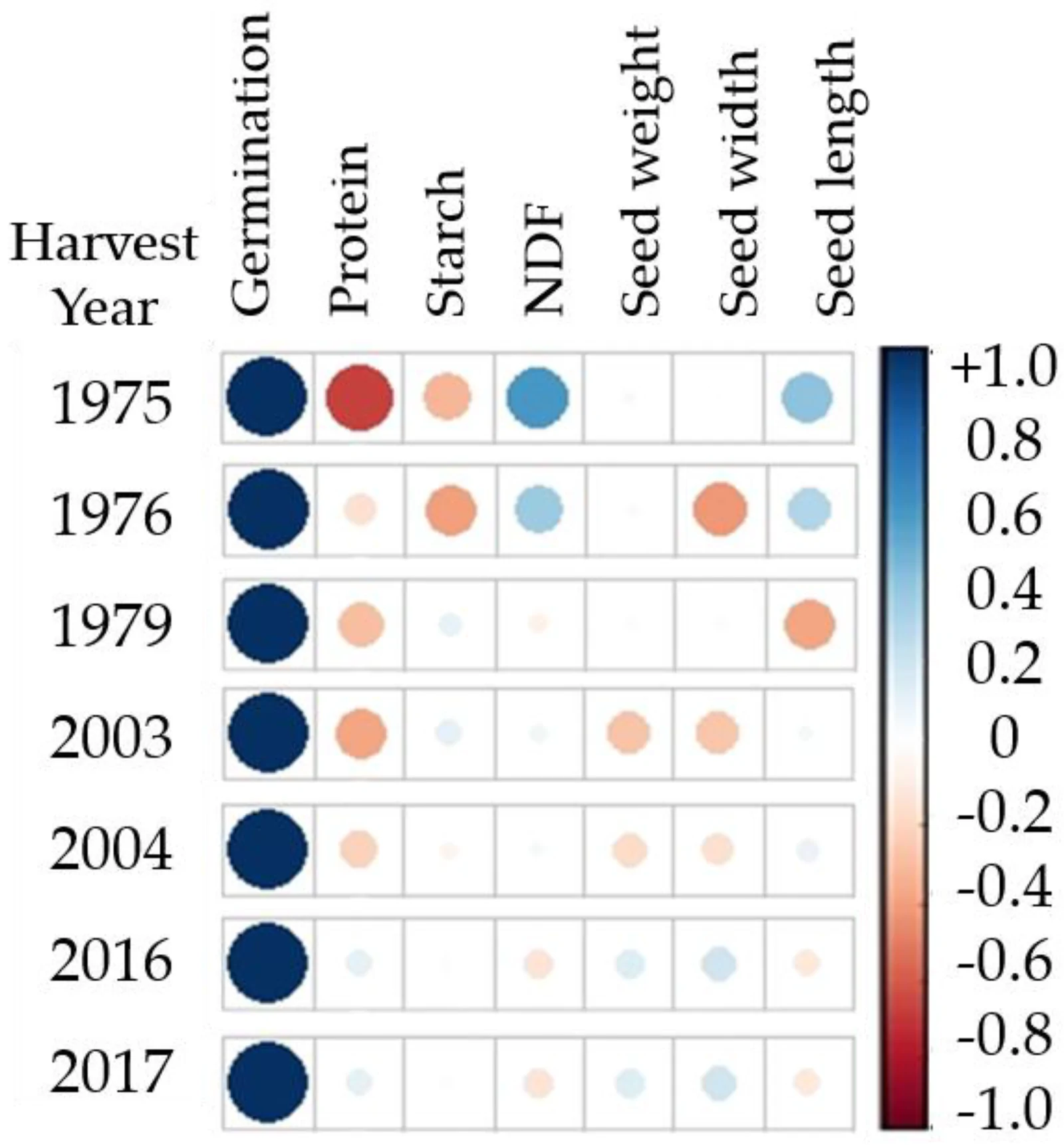
Correlation analysis of barley seed viability with grain protein, starch, and fibre content, seed weight, width, and length. The seed lots were harvested in seven different years listed on the left-hand side of the panel. Coloured circles illustrate the correlation analysis summary statistics. Blue and red imply positive and negative correlation, respectively, and colour depth represents the correlation coefficient (r) values, according to the key on the right-hand side. The bluer the colour, the greater the positive correlation coefficient (r); the redder the colour, the greater the negative correlation coefficient (r).

Correlation analyses revealed consistent negative associations between seed viability and grain protein content (GPC) in five of the seven HYs (Figure 2). No other trait showed comparable consistency. In the two earliest HYs (1975 and 1976), grain starch content was negatively correlated with viability, whereas NDF content was positively correlated. For grain-size traits (TGW, width, length), correlations were inconsistent across HYs: of 21 comparisons, 8 were negative, 7 positive, and 6 neutral. Overall, no quantitative trait showed a uniformly consistent relationship with conserved seed viability. Correlation coefficients (r) and significance values (P) are provided in Supplementary Table S3.

We further assessed associations between seed viability and nine highly heritable genebank descriptors: aleurone colour, lemma colour, rachilla hair length, awn length and presence, ear emergence date, spike density, spike type, number of spikelets per spike, and spike attitude. Based on the available data, we focused on four HYs: 1976, 2003, 2004, and 2016. No trait exhibited a consistent, statistically significant association across all four HYs (Table 1). However, in two HYs (1976 and 2004), viability was positively associated with early ear emergence and negatively associated with lemma pigmentation. Additionally, blue aleurone colour was positively associated with viability in HY 2003.

## 3. Discussion

Seed longevity under ex situ conservation is primarily determined by storage temperature, relative humidity, and oxygen availability [6]. In this study, we examined additional, subtler factors influencing seed viability within barley, asking to what extent plant and seed characteristics are associated with long-term germination outcomes. We analysed highly heritable genebank descriptors, including hull presence, flowering time, inflorescence morphology, and pigmentation. We complemented these with high-throughput, non-destructive phenotyping of grain macronutrient composition (protein, starch, and NDF) and seed morphometrics. As historical initial viability data were unavailable, we could not estimate longevity parameters such as P50 [5]. Instead, we used relative viability values within each harvest year (HY), allowing phenotypic associations to be assessed among accessions that shared comparable field and storage conditions, including sowing and harvest dates, the time between harvest and storage, and the duration of conservation. Seeds of wheat and barley stored at 4 °C in genebanks deteriorate more rapidly than samples stored at −20 °C, indicating that lower-temperature storage improves seed longevity during long-term conservation [18].

Nagel et al. [19] demonstrated intraspecific variation in cereal seed longevity within genebank environments. Induced viability decay as well as natural seed viability loss under −18 °C storage have been mapped to specific loci on bread wheat [2] and durum wheat [8] chromosomes. More recent Chip-SNP-based GWAS analyses identified SNP markers and candidate genes associated with seed viability decay [20,21]. Understanding seed longevity as a quantitative genetic trait has significant implications for ex situ conservation [22].

In rice, the *Rc* gene, which encodes a basic helix-loop-helix transcription factor, was recently identified as a key regulator of seed survival under ex situ storage [11]. ‘Longevity alleles’ may have been subject to evolutionary pressures during domestication. The shift from natural to anthropogenic seed dispersal likely favoured alleles that enhance seed survival. Consequently, such positive longevity regulators, potentially linked to other traits, may be enriched in genebank collections during long-term conservation.

Genome-wide association studies (GWAS) have become a valuable approach for identifying genomic regions underlying complex traits by leveraging historical recombination in diverse populations [23]. Recent GWAS studies in millets by Kannababu et al. [24,25] identified genomic regions associated with seed longevity traits in pearl millet and finger millet. In pearl millet, the SNP *PMSnpB394* on chromosome 2 was associated with 14 seed longevity traits, with candidate genes involved in hormone signalling, stress response, metabolism, and seed coat development processes. Similarly, in finger millet, the pleiotropic SNP *FM_SNP_9478* on chromosome 7B was associated with germination after ageing, and the candidate genes *DET1* and *Expansin-A2* contribute to seed coat integrity, thereby influencing germination and longevity.

The genetics of seed longevity have been extensively reviewed [10]. In addition to 22 studies in *Arabidopsis thaliana*, QTLs or markers associated with altered longevity have been reported in rice, barley, wheat, maize, soybean, lettuce, and tomato. Linking viability decay rates to genomic loci and heritable agronomic, morphological, and quality traits highlights the risk of changes in the genetic composition of genebank collections during long-term conservation. Recently, we developed a molecular labelling approach using genomic resources from barley and tetraploid wheat genebank-genomics projects, providing practical tools for monitoring accession identity, detecting misclassification and genetic inconsistencies, and supporting the maintenance of genetic integrity in cereal genebank collections [26]. The complexity of the genetic regulation of seed viability suggests that ex situ conservation may unintentionally select for longer-lived or more vigorous accessions. This could alter allelic frequencies in conserved populations, potentially causing unintended evolutionary changes.

### 3.1. Hull Presence Is a Major Determinant of Barley Seed Longevity

Hulless barley grains were substantially shorter-lived than hulled grains under cool-dry medium-term storage. This finding is consistent with earlier reports showing enhanced longevity of hulled cereals under ambient conditions, including barley, spelt wheat (*Triticum spelta*), and timothy (*Phleum pratense*) [27,28]. More recent work on oats by Shah et al. [29] reported that seeds stored with intact hulls retained viability, whereas seeds stored without hulls failed to germinate, highlighting the protective role of the hull during storage. Artificial ageing studies in barley have identified a major QTL near *nud* on chromosome 7H, the gene governing the hulled/hulless trait, as a key determinant of seed longevity [30,31,32]. Consistent with these previous findings, hulled accessions in our study exhibited higher viability across all tested HYs (Figure 1). Importantly, the magnitude of this effect was substantial in practical genebank terms. For example, 44–45-year-old hulled accessions from HY 1975 and 1976 retained average viabilities of 61.7% and 76.8%, respectively, whereas hulless accessions from the same years declined to 5.1% and 19%. This difference highlights the practical difference between a perfectly regenerable collection and one at risk of severe genetic erosion. Since existing barley longevity models were developed using hulled cultivars [7,33], our results indicate that hulled and hulless barley should be treated separately when predicting seed longevity and designing long-term conservation strategies.

### 3.2. Associations Between Seed Viability and Genebank Descriptors

After establishing hull presence as a dominant factor, subsequent analyses focused on hulled accessions to identify subtler associations. While our study did not examine interactions between hullness and other traits due to limited hulless sample size, it provides valuable insights into real-world viability decay using historic material that would be difficult to replicate experimentally. The observed associations do not imply causality; rather, they highlight repeated, statistically significant correlations. Biologically meaningful correlations may arise in several ways. They could reflect (i) direct or indirect effects of the trait on seed longevity, (ii) genetic linkage between the trait and loci regulating longevity, or (iii) shared regulatory pathways influencing both traits. Numerous examples of QTLs and markers associated with seed longevity support these genetic connections [10].

Flowering time provides a clear example of a potential direct effect. In two HYs (1976 and 2004), earlier ear emergence was associated with enhanced seed viability. The accessions evaluated originated from broad geographic ranges, spanning diverse climates and photoperiod regimes. Consequently, variation in flowering time likely reflects differential adaptation to UK field conditions, influencing grain-filling duration, maturation timing, and ambient conditions during seed desiccation, all of which are known to affect seed longevity. Similarly, the persistent husk in hulled barley may physically buffer the caryopsis against oxygen diffusion and temperature or humidity fluctuations, thereby enhancing seed longevity during storage.

### 3.3. Grain Protein Content Is Negatively Associated with Seed Viability

Among grain macronutrients, grain protein content (GPC) showed the most consistent relationship with seed viability. Across five older HYs, higher GPC was significantly associated with lower germination after prolonged storage (Figure 2). In contrast, starch and NDF exhibited no consistent associations.

NIR-based estimation of grain composition in barley is well established [34,35,36]. Although seed storage proteins are positively associated with longevity in *Arabidopsis* and are genetically linked [37,38], we observed a negative association between higher GPC and long-term seed viability in barley; the contribution of individual storage protein fractions (e.g., albumins, globulins, prolamins, glutenins) remains unknown. Studies in other crops provide further evidence that protein-related changes may contribute to seed deterioration. In quinoa, storage-related protein modifications, including protein insolubilisation and accumulation of advanced glycation end-products (AGEs), have been associated with seed deterioration and reduced seed quality [39]. In wheat, accumulation of AGEs during storage was also associated with reduced enzyme activity and germination, suggesting that protein-related modifications may contribute to seed deterioration [40]. Long-term ageing studies in wheat and barley have linked declining seed viability with ROS accumulation and lipid oxidation, highlighting the role of oxidative damage in seed deterioration [41].

Importantly, GPC in barley is a complex trait influenced by multiple genetic factors and strong genotype-by-environment interactions [42,43,44]. Previous studies linking high GPC to improved seedling vigour and yield manipulated protein content through environmental nitrogen inputs [45,46,47]. In contrast, our study assessed inherent, genetically influenced differences among accessions rather than environmentally induced variation.

Notably, negative associations between barley GPC and seed viability were also reported using NIR-based spectral analyses linked to germination and vigour parameters [48], in strong agreement with our findings. Together, these results indicate that higher GPC is associated with reduced long-term seed viability under ex situ storage, with implications for both conservation and utilisation.

## 4. Materials and Methods

### 4.1. Storage Conditions

Seeds were stored in non-sealed paper envelopes to allow free airflow within a large (600 m^3^) controlled-environment seed storage room maintained between 1.5 and 5 °C and 8–15% RH. The barley collections analysed in this study were consistently stored on the same set of shelving racks, with harvest years (HYs) grouped and retained together. To the best of our knowledge, seed lots were not separated during the storage period except when individual packets were temporarily removed for testing or distribution. Thus, each HY experienced identical conservation conditions from harvest onwards.

### 4.2. Germplasm

We analysed field-produced bulk seeds harvested at the John Innes Centre experimental field station (2000–2018) and at the PBI Cambridge (1972–1990). The barley accessions included in this study are listed in Supplementary Tables S1 and S2. All materials are publicly accessible via SeedStor (https://www.seedstor.ac.uk) as part of the UK contribution to the Multilateral System of the International Treaty on Plant Genetic Resources for Food and Agriculture (ITPGRFA).

### 4.3. Germination Test

Seeds were considered viable if both a healthy shoot and root (≥1 cm) developed. Hard, dormant seeds were scored as non-viable. For each accession, 50 seeds were tested using folded pleated cellulose paper (100%) moistened by ~1-s immersion in room-temperature filtered tap water and enclosed in a sealed plastic bag. After moistening, seeds were imbibed for three days at 6 °C and then transferred to 22 °C. Germination was assessed at 7, 14, and 18 days. The cumulative number of viable seeds across the three assessments was used to calculate the germination percentage.

Germination tests for HYs 2003, 2004, 2016, and 2017 were performed in 2020 (seed ages: 17, 16, 4, and 3 years, respectively). Tests for HYs 1974–1978 were conducted in 2019 (seed ages: 45, 44, 43, 42, and 41 years, respectively). HY 1979 was evaluated in 2023 (seed age: 44 years).

As an experimental control, during 2018–2020, we included a wheat cv. Paragon bulk harvested in 2004 at JIC; aliquots from the same seed source were tested alongside each germination experiment. Control germination averaged 84.3% (82.5–89.1%). For experiments conducted in 2023, barley cv. Minerva (harvested 2015) was used as the control and exhibited 94.2–100% germination, with a mean of 97%.

### 4.4. Statistical Analysis

Each HY was treated as an independent experiment because it represented a different set of barley accessions rather than the same seed lots assessed repeatedly during storage. Thus, harvest year and storage duration could not be independently separated from accession composition. Therefore, we compared germination among accessions within each harvest year, rather than estimating seed viability decay rates over time. Associations between germination rate and categorical (or discrete numeric) phenotypic traits from SeedStor were analysed using *t*-tests (two categories) or one-way ANOVA (three or more categories). Means and standard errors of the mean (SEM) were calculated in Microsoft Excel. Tukey-Kramer HSD tests and SEMs for Figure 1 were generated using JMP version 17.2 (SAS Institute).

Correlations between germination and continuous quantitative traits were calculated and visualised using the “corrplot” package in R v4.2.2. Exact *p*-values for all analyses are provided in the figures and tables. Statistical significance in the text was interpreted using a conventional α = 0.05 threshold.

### 4.5. Trait Definitions and Measurement Methods

#### 4.5.1. Macronutrient Analysis

Grain protein, starch, and neutral detergent fibre (NDF) contents were measured using Near-Infrared Spectroscopy (DA-7250 NIR Grain Analyser; Perten Instruments, Hägersten, Sweden). Values were normalised to grain dry weight using instrument-generated moisture calibrations.

Seed morphometrics: seed length, seed width, and thousand-grain weight (TGW) were measured using the Marvin Seed Analyser (GTA Sensorik GmbH, Wesel, Germany).

#### 4.5.2. Historic SeedStor Traits

SeedStor records include 69 traits representing 219,921 phenotypic data points for the barley collection (publicly accessible at https://www.seedstor.ac.uk). Nine traits were analysed here, selected for relevance to seed morphology, inflorescence characteristics, pigmentation, development, and composition. Trait names follow the Crop Ontology [49]; historical SeedStor terminology is indicated where different. These traits were historically recorded using the following standardised categorical descriptors.

Grain hullness—Categories: hulled/ hulless (SeedStor: Kernel covering: covered/ naked grain). Hulled grains retain lemma and palea after threshing; hulless grains lose the husk during threshing.

Lemma colour—Pigmentation of the lemma assessed visually at the early dough stage. SeedStor categories include yellow, violet, brown, black, grey, green, mixed, and orange. For this study, entries were grouped as yellow vs. pigmented (violet, brown, black, grey).

Spike attitude—Categories: erect/horizontal/pendulous (SeedStor: Ear Drop). Describes spike angle relative to the culm at harvest.

Awn length and awn presence—Categories: long/equal/short/no awn. Defined based on the relative length of the awn compared with the spike (Long: awn longer than spike; Equal: awn plus spike tip equals spike length; Short: awn shorter than the spike; No awn: complete absence of awns at harvest).

Rachilla hair length—Categories: long/short. Based on the estimated rachilla hair length on the ventral grain surface.

Spike type—Categories: 2-row/6-row. Determined by the fertility of lateral spikelets per rachis node, resulting in two or six visible rows of kernels.

Spike density—Categories: lax/average/dense. SeedStor defines nine classes (very lax to very dense) based on spikelet spacing (mm per spikelet); these were merged into three groups: Lax: >4.4 mm; Average: 2.5–3.9 mm; and Dense: <2.1 mm.

Ear emergence—Categories: early/typical/late. A relative measure of flowering time. The SeedStor database records five classes; “slightly early” and “slightly late” were merged with early and late, respectively. Ear emergence was defined as the stage at which 50% of tillers showed the inflorescence halfway emerged from the flag leaf.

Aleurone colour—Categories: blue/white. Indicates the presence or absence of visible blue anthocyanin pigmentation in the aleurone layer.

## 5. Conclusions

Genetic, environmental, and morphological factors interact to affect the decay of seed viability in a complex manner. As omics studies become feasible at a genebank scale, linking genomic regions to heritable plant phenotypes and seed longevity may contribute to more effective ex situ conservation of beneficial crop alleles.

In cool-dry medium-term conservation conditions, hulless barley seeds’ viability was significantly lower than that of hulled seeds. We recommend that barley curators consider practical decay rates of hulless accessions and plan separate regeneration cycles for hulled and hulless grain types.

In this study, barley grain protein content (GPC) was negatively correlated with seed viability. Therefore, GPC could also be a relevant factor in seed conservation.

## Figures and Tables

**Table 1:** Germination (mean %) and its association with 9 barley highly heritable traits descriptors with categorical values obtained from SeedStor historic data for seed lots from 4 harvest years. ‘*n*’ is the total number of measured accessions. Mean % is the average viability % of the related accessions. SEM is the standard error of the mean, and Pv is the calculated *p* value of the *t*-test (in case of two variables) or ANOVA test in case of more than two variables. Pv values below the accepted cut-off of 0.05, indicating statistically significant results. ND means no data was available in the historical record.

Traits	Parameter	Harvest Year 1976				Harvest Year 2003				Harvest Year 2004				Harvest Year 2016			
		*n*	Mean	SEM	Pv	*n*	Mean	SEM	Pv	*n*	Mean	SEM	Pv	*n*	Mean	SEM	Pv
Aleurone colour	Blue	40	64	6.6	0.36	21	94	0.3	0.03	54	94	0.1	0.11	14	92	1.4	0.24
	White	278	60	5.1		39	87	0.4		228	94	0		39	85	3.3	
Lemma colour	Pigmented	112	58	6.1	0.01	2	93	1	0.67	5	83	3.3	0.01	7	93	2.5	0.2
	Yellow	332	64	5.2		58	89	1.6		279	92	0.5		15	80	6.5	
Rachilla hair length	Long	56	77	2.5	0.67	46	90	1.9	0.36	228	92	0.6	0.83	31	87	3.1	0.74
	Short	33	78	2.8		14	87	2.4		56	92	1.1		18	86	4.9	
Awn length and awn presence	Long	40	80	2.1	0.08	28	92	1.6	0.41	82	93	0.8	0.06	11	90	5.4	0.56
	Equal	40	73	3.4		25	88	2.7		171	91	0.7		32	86	3.6	
	Short	5	76	6.1		2	95	1		2	98	2.4		ND	ND	ND	
	No awn	5	92	1.9		2	96	4		10	88	4.6		ND	ND	ND	
Ear emergence	Early	41	83	0.3	0.03	16	92	3.8	0.58	41	95	0.8	0.01	ND	ND	ND	
	Typical	19	71	1.0		12	87	4.5		29	89	2.1		5	92	3.7	0.6
	Late	25	78	0.6		32	89	1.6		212	92	0.6		47	88	2.2	
Ear laxness	Lax	23	76	3.3	0.37	15	88	3.6	0.85	41	93	1.3	0.78	15	86	4.7	0.4
	Average	52	79	2.3		34	90	2.3		141	92	0.7		17	80	5.9	
	Dense	7	86	6.0		9	91	2.3		86	92	0.9			ND	ND	
Node per ear	Few	66	80	2.0	0.31	46	91	1.5	0.31	139	92	0.7	0.233	15	75	7.2	0.244
	Average	15	73	4.4		8	84	7.3		79	93	0.7		8	88	4.1	
	Many	2	79	9.0		6	87	4.8		64	91	1.3			ND	ND	
Ear type	2 row	27	73	0.7	0.12	17	86	3.8	0.16	166	92	0	0.475	35	87	3.3	0.776
	6 row	63	79	0.3		43	91	1.6		118	92	0.1		9	85	6.1	
Ear Drop	Erect	6	72	6.4	0.33	16	90	3.4	0.98	68	91	1.2	0.515	16	85	4.6	0.195
	Horizontal	64	79	2.0		20	90	2.3		65	92	1.2		7	75	7.1	
	Pendulous	14	82	4.2		19	89	3.2		140	92	0.6		4	96	1.7	

## Data Availability

The original contributions presented in this study are included in the article/Supplementary Materials. Further inquiries can be directed to the corresponding author.

## Supplementary Materials

The following supporting information can be downloaded at: https://www.mdpi.com/article/10.3390/plants15192976/s1, Table S1: Germination rates of hulled and hulless accessions from the oldest seed lots (41–45 years old; harvested in 1974–1978) and from more recent harvest years (HY) 2003, 2004, 2016, and 2017; Table S2: All the phenotypes of hulled accessions from seven HYs (1975, 1976, 1979, 2003, 2004, 2016, and 2017) for the macronutrient and grain size data; Table S3: Summary statistics of correlation analysis of barley seed viability with grain protein, starch, and NDF content, seed weight, width, and length.
