# Supplementary material for: Enhanced Seed Viability Correlates with Husk Adhesion in Barley (Hordeum vulgare L.)
Source: Plants (Basel). 2026 Sep 29;15(19):2976. doi: 10.3390/plants15192976 (PMC13666928; doi:10.3390/plants15192976)
Supplement: Supplementary file 1 [file plants-15-02976-s001.zip › plants-4473565-supplementary.pdf]

**Supplementary Table S1. Germination rates of hulled and hulless accessions from the oldest seed lots (41-45 years old; harvested in 1974-1978) and from more recent harvest years (HY) 2003, 2004, 2016, and 2017.**

| Accession Code | Accession Name | Harvest Year | Grain type (Hulled/ Hulless) | Test year | Seed age (years) | Germination % |
|---|---|---|---|---|---|---|
| B18988 | ITJ Partial MR Ve-Pr 3 | 2017 | Hulled grains | 2020 | 3 | 100 |
| B19044 | ITJ Partial MR Pr-HS 10 | 2017 | Hulled grains | 2020 | 3 | 100 |
| B20079 | Fergie | 2017 | Hulled grains | 2020 | 3 | 100 |
| B20107 | VDH 905/81 | 2017 | Hulled grains | 2020 | 3 | 100 |
| B20167 | S BON 12 | 2017 | Hulled grains | 2020 | 3 | 100 |
| B20278 | Dae-Chon-Yuc-Kac | 2017 | Hulled grains | 2020 | 3 | 100 |
| B20461 | Druchingun-Sairai | 2017 | Hulled grains | 2020 | 3 | 100 |
| B3334 | Ankara 157/37 | 2017 | Hulled grains | 2020 | 3 | 100 |
| B8264 | North Carolina Hooded 26 | 2017 | Hulled grains | 2020 | 3 | 100 |
| B18948 | ITJ Partial MR Pr-GV 3 | 2017 | Hulled grains | 2020 | 3 | 98 |
| B19036 | ITJ Partial MR Pr-HS 2 | 2017 | Hulled grains | 2020 | 3 | 98 |
| B19052 | ITJ Partial MR Pr-Ve 1 | 2017 | Hulled grains | 2020 | 3 | 98 |
| B20556 | Franklin | 2017 | Hulled grains | 2020 | 3 | 98 |
| B5005 | BGM 35 Yellow Leaf | 2017 | Hulled grains | 2020 | 3 | 98 |
| B5919 | Welgevallen 48/54-2-5A | 2017 | Hulled grains | 2020 | 3 | 98 |
| B18964 | ITJ Partial MR HS-GV 4 | 2017 | Hulled grains | 2020 | 3 | 96 |
| B19012 | ITJ Partial MR Ku-GV 7 | 2017 | Hulled grains | 2020 | 3 | 96 |
| B20314 | Orge 14j | 2017 | Hulled grains | 2020 | 3 | 96 |
| B18996 | ITJ Partial MR Ve-Pr 11 | 2017 | Hulled grains | 2020 | 3 | 94 |
| B19060 | ITJ Partial MR Pr-Ve 9 | 2017 | Hulled grains | 2020 | 3 | 94 |
| B19871 | 19635 Co 90 | 2017 | Hulled grains | 2020 | 3 | 94 |
| B19884 | CI 3411 | 2017 | Hulled grains | 2020 | 3 | 94 |
| B20048 | HJ 117-940-15 | 2017 | Hulled grains | 2020 | 3 | 94 |

| | | | | | | |
|---|---|---|---|---|---|---|
| B20130 | Assala-06 | 2017 | Hulled grains | 2020 | 3 | 94 |
| B20156 | Er*Apm | 2017 | Hulled grains | 2020 | 3 | 94 |
| B20397 | Zairai-Rokkaku | 2017 | Hulled grains | 2020 | 3 | 94 |
| B4052 | Afghan R 1041 | 2017 | Hulled grains | 2020 | 3 | 94 |
| B19020 | ITJ Partial MR Ku-GV 15 | 2017 | Hulled grains | 2020 | 3 | 92 |
| B20234 | Belts 60-1809 | 2017 | Hulled grains | 2020 | 3 | 92 |
| B20267 | IAR/B 326-4 | 2017 | Hulled grains | 2020 | 3 | 92 |
| B19004 | ITJ Partial MR GP-Pr 6 | 2017 | Hulled grains | 2020 | 3 | 90 |
| B20216 | CI 8618 | 2017 | Hulled grains | 2020 | 3 | 90 |
| B20296 | Suwon 15 | 2017 | Hulled grains | 2020 | 3 | 90 |
| B20372 | Pao An Tien 3 | 2017 | Hulled grains | 2020 | 3 | 90 |
| B20548 | NB 96-4 | 2017 | Hulled grains | 2020 | 3 | 90 |
| B18972 | ITJ Partial MR HS-GV 12 | 2017 | Hulled grains | 2020 | 3 | 88 |
| B19028 | ITJ Partial MR GP-HS 8 | 2017 | Hulled grains | 2020 | 3 | 88 |
| B20039 | HLB 19-3-12 | 2017 | Hulled grains | 2020 | 3 | 88 |
| B20975 | Scholar | 2017 | Hulled grains | 2020 | 3 | 88 |
| B18980 | ITJ Partial MR HS-GV 20 | 2017 | Hulled grains | 2020 | 3 | 86 |
| B5243 | Welgevallen 65/55-19 | 2017 | Hulled grains | 2020 | 3 | 86 |
| B18864 | Taylor Y 446 | 2017 | Hulled grains | 2020 | 3 | 84 |
| B18956 | ITJ Partial MR Pr-GV 11 | 2017 | Hulled grains | 2020 | 3 | 82 |
| B20185 | WI 2291 | 2017 | Hulled grains | 2020 | 3 | 82 |
| B20259 | IAR/B 410-1 | 2017 | Hulled grains | 2020 | 3 | 80 |
| B20540 | NB 92 | 2017 | Hulled grains | 2020 | 3 | 76 |
| B4324 | Hansen 181b | 2017 | Hulled grains | 2020 | 3 | 76 |
| B20138 | C 63 | 2017 | Hulled grains | 2020 | 3 | 72 |
| B17911 | Ethiopia R 364/4 | 2017 | Hulled grains | 2020 | 3 | 70 |
| B20288 | Suwon 5 | 2017 | Hulled grains | 2020 | 3 | 68 |
| B3754 | Ethiopia R 39 | 2017 | Hulled grains | 2020 | 3 | 66 |
| B18940 | ITJ Partial MR St-HS 3 | 2017 | Hulled grains | 2020 | 3 | 62 |

| | | | | | | |
|---|---|---|---|---|---|---|
| B20408 | Chikurin | 2017 | Hulled grains | 2020 | 3 | 62 |
| B7673 | Bere | 2017 | Hulled grains | 2020 | 3 | 62 |
| B19535 | Trifolium | 2017 | Hulled grains | 2020 | 3 | 60 |
| B17604 | Ethiopia K 549 | 2017 | Hulled grains | 2020 | 3 | 44 |
| B20202 | Ethiopia | 2017 | Hulled grains | 2020 | 3 | 44 |
| B3835 | Ethiopia R 507 | 2017 | Hulled grains | 2020 | 3 | 44 |
| B20175 | S BON 79 | 2017 | Hulled grains | 2020 | 3 | 24 |
| B20250 | GAW 104-7 | 2017 | Hulled grains | 2020 | 3 | 24 |
| B16843 | Pakistan 63 BN-1 | 2017 | Hulless | 2020 | 3 | 100 |
| B17815 | Ethiopia R 110/2 | 2017 | Hulless | 2020 | 3 | 100 |
| B19860 | Tibet Walston 2/2 | 2017 | Hulless | 2020 | 3 | 100 |
| B20358 | Ta Kung Kuan | 2017 | Hulless | 2020 | 3 | 100 |
| B3711 | Kobin Katagi | 2017 | Hulless | 2020 | 3 | 100 |
| B7157 | Excelsior | 2017 | Hulless | 2020 | 3 | 100 |
| B19577 | CI 7751 | 2017 | Hulless | 2020 | 3 | 98 |
| B20376 | Chiao Chuang 2 | 2017 | Hulless | 2020 | 3 | 98 |
| B5063 | Zoned Leaf zbc | 2017 | Hulless | 2020 | 3 | 98 |
| B17664 | Ethiopia K 644 | 2017 | Hulless | 2020 | 3 | 96 |
| B20322 | CI 9281 | 2017 | Hulless | 2020 | 3 | 96 |
| B5868 | Welgevallen 526-1-1 | 2017 | Hulless | 2020 | 3 | 96 |
| B6564 | Pakistan 33 BN-1 | 2017 | Hulless | 2020 | 3 | 94 |
| B16908 | Pakistan 72B BN-1 | 2017 | Hulless | 2020 | 3 | 94 |
| B20427 | CI 9416 | 2017 | Hulless | 2020 | 3 | 94 |
| B20413 | CI 9396 | 2017 | Hulless | 2020 | 3 | 92 |
| B20466 | Mushinchiang 2 | 2017 | Hulless | 2020 | 3 | 92 |
| B7092 | Turkey R 539 | 2017 | Hulless | 2020 | 3 | 92 |
| B7429 | Hizahachi | 2017 | Hulless | 2020 | 3 | 92 |
| B16770 | Pakistan 52 BN-5 | 2017 | Hulless | 2020 | 3 | 88 |
| B17044 | Pakistan 82 BN-1 | 2017 | Hulless | 2020 | 3 | 88 |

| | | | | | | |
|---|---|---|---|---|---|---|
| B17877 | Ethiopia R 265/3 | 2017 | Hulless | 2020 | 3 | 88 |
| B8024 | Bozu Mochi | 2017 | Hulless | 2020 | 3 | 88 |
| B9437 | GAW 103-1 | 2017 | Hulless | 2020 | 3 | 88 |
| B19540 | Nackte von Nepal | 2017 | Hulless | 2020 | 3 | 86 |
| B20446 | Kikinshiraze | 2017 | Hulless | 2020 | 3 | 86 |
| B4439 | Hindukusch | 2017 | Hulless | 2020 | 3 | 86 |
| B6529 | Pakistan 31B BN-9 | 2017 | Hulless | 2020 | 3 | 86 |
| B19715 | India 128 BC-2 | 2017 | Hulless | 2020 | 3 | 84 |
| B20276 | Chae-Rae-Yuk-Kac | 2017 | Hulless | 2020 | 3 | 84 |
| B20365 | Mu Shih Chiang 2 | 2017 | Hulless | 2020 | 3 | 84 |
| B4979 | BGM 7 Round Seed | 2017 | Hulless | 2020 | 3 | 84 |
| B20438 | Kosaba 1 | 2017 | Hulless | 2020 | 3 | 82 |
| B4067 | Afghan R 1184 | 2017 | Hulless | 2020 | 3 | 82 |
| B5364 | Pakistan 30 BN | 2017 | Hulless | 2020 | 3 | 82 |
| B6239 | India 144 BN | 2017 | Hulless | 2020 | 3 | 82 |
| B7517 | Triple Awn Lemma tr | 2017 | Hulless | 2020 | 3 | 82 |
| B5354 | Pakistan 16 BN | 2017 | Hulless | 2020 | 3 | 80 |
| B17496 | Ethiopia K 404 | 2017 | Hulless | 2020 | 3 | 80 |
| B4825 | Phari Tibet | 2017 | Hulless | 2020 | 3 | 80 |
| B6220 | India 126 BN | 2017 | Hulless | 2020 | 3 | 80 |
| B16725 | Pakistan 48 BN-1 | 2017 | Hulless | 2020 | 3 | 78 |
| B8362 | Ethiopia | 2017 | Hulless | 2020 | 3 | 78 |
| B20041 | PA 656 | 2017 | Hulless | 2020 | 3 | 76 |
| B16937 | Pakistan 74B BN-1 | 2017 | Hulless | 2020 | 3 | 66 |
| B19480 | White Hulless | 2017 | Hulless | 2020 | 3 | 66 |
| B17430 | Ethiopia K 268 | 2017 | Hulless | 2020 | 3 | 64 |
| B3550 | Orzo Palestina Nuda | 2017 | Hulless | 2020 | 3 | 62 |
| B3798 | Ethiopia R 270 | 2017 | Hulless | 2020 | 3 | 62 |
| B19685 | India 112 BN-1 | 2017 | Hulless | 2020 | 3 | 58 |

| | | | | | | |
|---|---|---|---|---|---|---|
| B3814 | Ethiopia R 363 | 2017 | Hulless | 2020 | 3 | 58 |
| B5363 | Pakistan 29 BN | 2017 | Hulless | 2020 | 3 | 58 |
| B18892 | N 23A-9 | 2017 | Hulless | 2020 | 3 | 48 |
| B16807 | Pakistan 56 BN-7 | 2017 | Hulless | 2020 | 3 | 44 |
| B5391 | Pakistan 59 BN | 2017 | Hulless | 2020 | 3 | 44 |
| B16702 | Pakistan 46 BN-1 | 2017 | Hulless | 2020 | 3 | 28 |
| B17070 | Pakistan 85 BN-1 | 2017 | Hulless | 2020 | 3 | 28 |
| B16667 | Pakistan 41 BN-1 | 2017 | Hulless | 2020 | 3 | 26 |
| B16885 | Pakistan 69 BN-1 | 2017 | Hulless | 2020 | 3 | 18 |
| B19472 | Ianthinum Korn | 2017 | Hulless | 2020 | 3 | 90 |
| B17414 | Cintruenigo | 2016 | Hulled grains | 2020 | 4 | 98 |
| B17685 | Ethiopia K 688 | 2016 | Hulled grains | 2020 | 4 | 76 |
| B17707 | Ethiopia K 713 | 2016 | Hulled grains | 2020 | 4 | 88 |
| B17727 | Ethiopia K 737 | 2016 | Hulled grains | 2020 | 4 | 90 |
| B17746 | Ethiopia K 757 | 2016 | Hulled grains | 2020 | 4 | 68 |
| B17768 | Ethiopia K 302 | 2016 | Hulled grains | 2020 | 4 | 94 |
| B18393 | Globe | 2016 | Hulled grains | 2020 | 4 | 100 |
| B18587 | Kerry | 2016 | Hulled grains | 2020 | 4 | 96 |
| B20476 | Chikurin Ibaraki 1 | 2016 | Hulled grains | 2020 | 4 | 100 |
| B20906 | #N/A | 2016 | Hulled grains | 2020 | 4 | 94 |
| B20929 | #N/A | 2016 | Hulled grains | 2020 | 4 | 84 |
| B20943 | #N/A | 2016 | Hulled grains | 2020 | 4 | 96 |
| B20957 | #N/A | 2016 | Hulled grains | 2020 | 4 | 100 |
| B24234 | Bandulla | 2016 | Hulled grains | 2020 | 4 | 92 |
| B3342 | Cb 272/2 | 2016 | Hulled grains | 2020 | 4 | 98 |
| B3647 | Morocco Tilougguite | 2016 | Hulled grains | 2020 | 4 | 94 |
| B4441 | Hindukusch | 2016 | Hulled grains | 2020 | 4 | 10 |
| B4929 | Ethiopia | 2016 | Hulled grains | 2020 | 4 | 80 |
| B4971 | Ethiopia | 2016 | Hulled grains | 2020 | 4 | 74 |

| | | | | | | |
|---|---|---|---|---|---|---|
| B5321 | Dickson | 2016 | Hulled grains | 2020 | 4 | 38 |
| B5771 | Amakei 18 | 2016 | Hulled grains | 2020 | 4 | 78 |
| B6067 | Akagi | 2016 | Hulled grains | 2020 | 4 | 94 |
| B6178 | Allegro | 2016 | Hulled grains | 2020 | 4 | 92 |
| B7088 | Turkey R 495 | 2016 | Hulled grains | 2020 | 4 | 30 |
| B7105 | Turkey R 753 | 2016 | Hulled grains | 2020 | 4 | 62 |
| B7119 | Turkey R 1784 | 2016 | Hulled grains | 2020 | 4 | 98 |
| B7133 | Turkey R 2082 | 2016 | Hulled grains | 2020 | 4 | 66 |
| B7229 | Angora | 2016 | Hulled grains | 2020 | 4 | 94 |
| B7315 | USSR Don | 2016 | Hulled grains | 2020 | 4 | 94 |
| B7364 | Pallidum Suer | 2016 | Hulled grains | 2020 | 4 | 94 |
| B7580 | Algerian CI 1179 | 2016 | Hulled grains | 2020 | 4 | 82 |
| B7818 | Afghan R 214 | 2016 | Hulled grains | 2020 | 4 | 92 |
| B8065 | Algerian CI 1179 | 2016 | Hulled grains | 2020 | 4 | 94 |
| B8389 | Ragusa 6-9, Hybernum | 2016 | Hulled grains | 2020 | 4 | 96 |
| B8503 | Egypt 4 | 2016 | Hulled grains | 2020 | 4 | 94 |
| B8965 | Turkey | 2016 | Hulled grains | 2020 | 4 | 98 |
| B8980 | Turkey | 2016 | Hulled grains | 2020 | 4 | 100 |
| B8994 | Turkey | 2016 | Hulled grains | 2020 | 4 | 98 |
| B9009 | Turkey | 2016 | Hulled grains | 2020 | 4 | 100 |
| B9024 | Turkey | 2016 | Hulled grains | 2020 | 4 | 94 |
| B9038 | Turkey | 2016 | Hulled grains | 2020 | 4 | 94 |
| B9052 | Turkey | 2016 | Hulled grains | 2020 | 4 | 92 |
| B9070 | Turkey | 2016 | Hulled grains | 2020 | 4 | 96 |
| B9085 | Turkey | 2016 | Hulled grains | 2020 | 4 | 94 |
| B9100 | Turkey PI 342165 | 2016 | Hulled grains | 2020 | 4 | 96 |
| B9122 | Turkey PI 342187 | 2016 | Hulled grains | 2020 | 4 | 66 |
| B9146 | Turkey PI 342213 | 2016 | Hulled grains | 2020 | 4 | 98 |
| B9164 | Turkey PI 342238 | 2016 | Hulled grains | 2020 | 4 | 90 |

| | | | | | | |
|---|---|---|---|---|---|---|
| B9187 | Turkey | 2016 | Hulled grains | 2020 | 4 | 96 |
| B9206 | Turkey | 2016 | Hulled grains | 2020 | 4 | 98 |
| B9229 | GAW 14-2 | 2016 | Hulled grains | 2020 | 4 | 98 |
| B9250 | GAW 36-1 | 2016 | Hulled grains | 2020 | 4 | 94 |
| B9293 | GAW 84A-2 | 2016 | Hulled grains | 2020 | 4 | 86 |
| B9317 | GAW 111-1 | 2016 | Hulled grains | 2020 | 4 | 86 |
| B9358 | GAW 151-6 | 2016 | Hulled grains | 2020 | 4 | 98 |
| B9385 | GAW 77-3 | 2016 | Hulled grains | 2020 | 4 | 100 |
| B9409 | GAW 136-1 | 2016 | Hulled grains | 2020 | 4 | 94 |
| B9441 | GAW 114-3 | 2016 | Hulled grains | 2020 | 4 | 100 |
| B19632 | Corona | 2016 | Hulled grains | 2020 | 4 | 92 |
| B3748 | Guiza 117 | 2016 | Hulled grains | 2020 | 4 | 92 |
| B17650 | Ethiopia K 613 | 2016 | Hulless | 2020 | 4 | 76 |
| B17664 | Ethiopia K 644 | 2016 | Hulless | 2020 | 4 | 98 |
| B18910 | Nudimelancrothnon | 2016 | Hulless | 2020 | 4 | 92 |
| B20474 | Mihori Hadaka 3 | 2016 | Hulless | 2020 | 4 | 96 |
| B20814 | Nubet | 2016 | Hulless | 2020 | 4 | 100 |
| B3471 | Hokuto Hadaka | 2016 | Hulless | 2020 | 4 | 100 |
| B3501 | Jet | 2016 | Hulless | 2020 | 4 | 100 |
| B3697 | Shimabara | 2016 | Hulless | 2020 | 4 | 88 |
| B3723 | Mochi Mugi | 2016 | Hulless | 2020 | 4 | 88 |
| B4059 | Afghan R 1117 | 2016 | Hulless | 2020 | 4 | 28 |
| B4068 | Afghan R 1208 | 2016 | Hulless | 2020 | 4 | 90 |
| B4073 | Afghan R 1340 | 2016 | Hulless | 2020 | 4 | 70 |
| B4167 | Esedra | 2016 | Hulless | 2020 | 4 | 82 |
| B4171 | Pisa | 2016 | Hulless | 2020 | 4 | 68 |
| B4238 | NARB 6211 | 2016 | Hulless | 2020 | 4 | 14 |
| B4413 | Prokupkuv Nahy | 2016 | Hulless | 2020 | 4 | 98 |
| B4428 | Jaydee | 2016 | Hulless | 2020 | 4 | 58 |

| | | | | | | |
|---|---|---|---|---|---|---|
| B4584 | Hiproly | 2016 | Hulless | 2020 | 4 | 52 |
| B5827 | China | 2016 | Hulless | 2020 | 4 | 86 |
| B6124 | Nudinka | 2016 | Hulless | 2020 | 4 | 100 |
| B7146 | Maksimir 202 | 2016 | Hulless | 2020 | 4 | 92 |
| B7979 | Hiproly | 2016 | Hulless | 2020 | 4 | 94 |
| B8271 | Tanbajiro | 2016 | Hulless | 2020 | 4 | 96 |
| B9435 | GAW 101-1 | 2016 | Hulless | 2020 | 4 | 92 |
| B3345 | C 205 | 2004 | Hulled grains | 2020 | 16 | 94 |
| B3346 | Cb 283/3 | 2004 | Hulled grains | 2020 | 16 | 96 |
| B3352 | Konia | 2004 | Hulled grains | 2020 | 16 | 88 |
| B3359 | Odesski 17 | 2004 | Hulled grains | 2020 | 16 | 96 |
| B3382 | HB 199/74/2/1 | 2004 | Hulled grains | 2020 | 16 | 72 |
| B3384 | Akan Mugi | 2004 | Hulled grains | 2020 | 16 | 96 |
| B3389 | Ariel | 2004 | Hulled grains | 2020 | 16 | 78 |
| B3397 | Ethiopia 11 | 2004 | Hulled grains | 2020 | 16 | 100 |
| B3400 | Amsel | 2004 | Hulled grains | 2020 | 16 | 86 |
| B3401 | Agio | 2004 | Hulled grains | 2020 | 16 | 100 |
| B3402 | Arabische 4716 | 2004 | Hulled grains | 2020 | 16 | 100 |
| B3405 | Abed 0166 | 2004 | Hulled grains | 2020 | 16 | 100 |
| B3406 | Abed 0-44 | 2004 | Hulled grains | 2020 | 16 | 76 |
| B3407 | Abed 0-43 | 2004 | Hulled grains | 2020 | 16 | 94 |
| B3409 | Abed Denso | 2004 | Hulled grains | 2020 | 16 | 94 |
| B3411 | Abed 0315 | 2004 | Hulled grains | 2020 | 16 | 92 |
| B3412 | Abed 0193 | 2004 | Hulled grains | 2020 | 16 | 86 |
| B3415 | Beikoku 4 | 2004 | Hulled grains | 2020 | 16 | 90 |
| B3418 | Batna | 2004 | Hulled grains | 2020 | 16 | 74 |
| B3419 | Browarny Pzhr | 2004 | Hulled grains | 2020 | 16 | 68 |
| B3427 | Bankuti Korai | 2004 | Hulled grains | 2020 | 16 | 100 |
| B3429 | Barin | 2004 | Hulled grains | 2020 | 16 | 84 |

| | | | | | | |
|---|---|---|---|---|---|---|
| B3431 | Chevallier Chile | 2004 | Hulled grains | 2020 | 16 | 94 |
| B3434 | CIV 1131 | 2004 | Hulled grains | 2020 | 16 | 84 |
| B3442 | Compana | 2004 | Hulled grains | 2020 | 16 | 72 |
| B3447 | Dioszegi 625 Sor | 2004 | Hulled grains | 2020 | 16 | 100 |
| B3449 | Ebro | 2004 | Hulled grains | 2020 | 16 | 100 |
| B3455 | Elsa | 2004 | Hulled grains | 2020 | 16 | 72 |
| B3457 | France 21 | 2004 | Hulled grains | 2020 | 16 | 84 |
| B3460 | Firlbecks 3 | 2004 | Hulled grains | 2020 | 16 | 90 |
| B3461 | Freja | 2004 | Hulled grains | 2020 | 16 | 92 |
| B3462 | Gateway | 2004 | Hulled grains | 2020 | 16 | 90 |
| B3464 | Gotlands | 2004 | Hulled grains | 2020 | 16 | 92 |
| B3465 | Gold | 2004 | Hulled grains | 2020 | 16 | 94 |
| B3466 | Goldthorpe | 2004 | Hulled grains | 2020 | 16 | 98 |
| B3468 | Gordon 55 | 2004 | Hulled grains | 2020 | 16 | 90 |
| B3470 | Guld | 2004 | Hulled grains | 2020 | 16 | 100 |
| B3472 | Hakata 2 | 2004 | Hulled grains | 2020 | 16 | 90 |
| B3473 | Harubin | 2004 | Hulled grains | 2020 | 16 | 90 |
| B3474 | Harada Omugi | 2004 | Hulled grains | 2020 | 16 | 98 |
| B3476 | Haisa 2 | 2004 | Hulled grains | 2020 | 16 | 98 |
| B3477 | Haisa 1 | 2004 | Hulled grains | 2020 | 16 | 84 |
| B3479 | Heimdal | 2004 | Hulled grains | 2020 | 16 | 90 |
| B3480 | Helmi | 2004 | Hulled grains | 2020 | 16 | 88 |
| B3481 | Hunter | 2004 | Hulled grains | 2020 | 16 | 96 |
| B3483 | Hatvani Sor | 2004 | Hulled grains | 2020 | 16 | 90 |
| B3488 | HC 23 | 2004 | Hulled grains | 2020 | 16 | 96 |
| B3494 | Iceland 1 | 2004 | Hulled grains | 2020 | 16 | 96 |
| B3498 | Imperial 1554 | 2004 | Hulled grains | 2020 | 16 | 100 |
| B3499 | Imperial 1552 | 2004 | Hulled grains | 2020 | 16 | 90 |
| B3506 | Kazimierski | 2004 | Hulled grains | 2020 | 16 | 92 |

| | | | | | | |
|---|---|---|---|---|---|---|
| B3508 | Kaluga | 2004 | Hulled grains | 2020 | 16 | 94 |
| B3516 | Louhi | 2004 | Hulled grains | 2020 | 16 | 98 |
| B3519 | Mazowiecki | 2004 | Hulled grains | 2020 | 16 | 50 |
| B3521 | Nutans Moskva | 2004 | Hulled grains | 2020 | 16 | 98 |
| B3525 | MGH 55221 | 2004 | Hulled grains | 2020 | 16 | 94 |
| B3528 | Maltworthy | 2004 | Hulled grains | 2020 | 16 | 96 |
| B3529 | Multan | 2004 | Hulled grains | 2020 | 16 | 90 |
| B3545 | Odessa | 2004 | Hulled grains | 2020 | 16 | 92 |
| B3570 | Ricardo | 2004 | Hulled grains | 2020 | 16 | 98 |
| B3581 | Standwell | 2004 | Hulled grains | 2020 | 16 | 94 |
| B3588 | Stephan | 2004 | Hulled grains | 2020 | 16 | 78 |
| B3590 | Seringei | 2004 | Hulled grains | 2020 | 16 | 90 |
| B3593 | Smyrnakorn 3 | 2004 | Hulled grains | 2020 | 16 | 94 |
| B3595 | Smyrnakorn | 2004 | Hulled grains | 2020 | 16 | 92 |
| B3597 | Shunsei | 2004 | Hulled grains | 2020 | 16 | 92 |
| B3601 | Tiflis | 2004 | Hulled grains | 2020 | 16 | 90 |
| B3603 | T * B | 2004 | Hulled grains | 2020 | 16 | 94 |
| B3605 | Taktaharkanyi Hanna | 2004 | Hulled grains | 2020 | 16 | 92 |
| B3608 | Vagabond | 2004 | Hulled grains | 2020 | 16 | 96 |
| B3610 | Vankkuri | 2004 | Hulled grains | 2020 | 16 | 98 |
| B3611 | Volla | 2004 | Hulled grains | 2020 | 16 | 74 |
| B3612 | Vollkorngerste | 2004 | Hulled grains | 2020 | 16 | 82 |
| B3616 | Britta | 2004 | Hulled grains | 2020 | 16 | 96 |
| B3618 | Wase Rokkaku | 2004 | Hulled grains | 2020 | 16 | 94 |
| B3619 | Boreham Warrior | 2004 | Hulled grains | 2020 | 16 | 98 |
| B3622 | HB 248/17/4 | 2004 | Hulled grains | 2020 | 16 | 96 |
| B3624 | HB 40/66/5 | 2004 | Hulled grains | 2020 | 16 | 94 |
| B3625 | HB 34/2/3/33 | 2004 | Hulled grains | 2020 | 16 | 92 |
| B3626 | HB 256/3/2/2 | 2004 | Hulled grains | 2020 | 16 | 92 |

| | | | | | | |
|---|---|---|---|---|---|---|
| B3628 | HB 281/6/6/5 | 2004 | Hulled grains | 2020 | 16 | 94 |
| B3630 | HB 279/5/1/3 | 2004 | Hulled grains | 2020 | 16 | 92 |
| B3635 | Liberty | 2004 | Hulled grains | 2020 | 16 | 58 |
| B3636 | Lyallpur 3642 | 2004 | Hulled grains | 2020 | 16 | 84 |
| B3638 | No 710 | 2004 | Hulled grains | 2020 | 16 | 94 |
| B3643 | Conway | 2004 | Hulled grains | 2020 | 16 | 94 |
| B3645 | Morocco Z Ahanesal | 2004 | Hulled grains | 2020 | 16 | 88 |
| B3646 | Morocco Azilal | 2004 | Hulled grains | 2020 | 16 | 100 |
| B3648 | Morocco Marrakech | 2004 | Hulled grains | 2020 | 16 | 92 |
| B3650 | Morocco Imilchil | 2004 | Hulled grains | 2020 | 16 | 96 |
| B3651 | Morocco Meknes | 2004 | Hulled grains | 2020 | 16 | 94 |
| B3653 | Precoce du Maroc 227b | 2004 | Hulled grains | 2020 | 16 | 92 |
| B3655 | Morocco 89 | 2004 | Hulled grains | 2020 | 16 | 90 |
| B3656 | Morocco 077 | 2004 | Hulled grains | 2020 | 16 | 88 |
| B3657 | Morocco 071 | 2004 | Hulled grains | 2020 | 16 | 94 |
| B3658 | Simmonds 2 | 2004 | Hulled grains | 2020 | 16 | 94 |
| B3659 | Simmonds 1 | 2004 | Hulled grains | 2020 | 16 | 84 |
| B3670 | Rogers | 2004 | Hulled grains | 2020 | 16 | 100 |
| B3672 | Comma | 2004 | Hulled grains | 2020 | 16 | 100 |
| B3674 | Philip | 2004 | Hulled grains | 2020 | 16 | 98 |
| B3679 | Nr 5652 | 2004 | Hulled grains | 2020 | 16 | 100 |
| B3680 | Nr 1241 | 2004 | Hulled grains | 2020 | 16 | 72 |
| B3683 | Nutans 6040 | 2004 | Hulled grains | 2020 | 16 | 100 |
| B3685 | ligeva 1104 | 2004 | Hulled grains | 2020 | 16 | 92 |
| B3686 | Leningradsky | 2004 | Hulled grains | 2020 | 16 | 98 |
| B3689 | Giaginsky | 2004 | Hulled grains | 2020 | 16 | 90 |
| B3690 | ColchiCum 10/30 | 2004 | Hulled grains | 2020 | 16 | 92 |
| B3718 | Opal B | 2004 | Hulled grains | 2020 | 16 | 96 |
| B3721 | Nikaku Chevallier | 2004 | Hulled grains | 2020 | 16 | 94 |

| | | | | | | |
|---|---|---|---|---|---|---|
| B3722 | Hanna | 2004 | Hulled grains | 2020 | 16 | 82 |
| B3725 | Kagoshima Golden Melon | 2004 | Hulled grains | 2020 | 16 | 96 |
| B3726 | Tochigi Golden Melon | 2004 | Hulled grains | 2020 | 16 | 90 |
| B3727 | Morocco | 2004 | Hulled grains | 2020 | 16 | 98 |
| B3730 | Mosane | 2004 | Hulled grains | 2020 | 16 | 90 |
| B3732 | Moravian Barley | 2004 | Hulled grains | 2020 | 16 | 98 |
| B3746 | Astrid | 2004 | Hulled grains | 2020 | 16 | 98 |
| B3747 | Mamie | 2004 | Hulled grains | 2020 | 16 | 94 |
| B3954 | Cebada Negra Manfredi | 2004 | Hulled grains | 2020 | 16 | 86 |
| B3957 | HB 293/5/3/18/1 | 2004 | Hulled grains | 2020 | 16 | 98 |
| B3958 | HB 293/25/2/8/1 | 2004 | Hulled grains | 2020 | 16 | 100 |
| B3963 | Rika * F1(Baladi 16 * Rika)33 | 2004 | Hulled grains | 2020 | 16 | 98 |
| B3965 | BK * 279.711)53-5 YR Resistant | 2004 | Hulled grains | 2020 | 16 | 88 |
| B3983 | Grignon | 2004 | Hulled grains | 2020 | 16 | 94 |
| B3992 | Latisglumatum | 2004 | Hulled grains | 2020 | 16 | 96 |
| B3997 | Ledeci Beta 40 | 2004 | Hulled grains | 2020 | 16 | 96 |
| B3998 | Martonvasar FB 102 | 2004 | Hulled grains | 2020 | 16 | 82 |
| B4000 | Mezohetvesi 68 | 2004 | Hulled grains | 2020 | 16 | 78 |
| B4018 | BT Hazera 127/1 | 2004 | Hulled grains | 2020 | 16 | 96 |
| B4114 | Cordova | 2004 | Hulled grains | 2020 | 16 | 94 |
| B4148 | Ta b7266 | 2004 | Hulled grains | 2020 | 16 | 100 |
| B4151 | Arvo | 2004 | Hulled grains | 2020 | 16 | 90 |
| B4159 | Linha de Arivat | 2004 | Hulled grains | 2020 | 16 | 98 |
| B4160 | EAN 97599 | 2004 | Hulled grains | 2020 | 16 | 94 |
| B4161 | Juromenha | 2004 | Hulled grains | 2020 | 16 | 100 |
| B4174 | Turk | 2004 | Hulled grains | 2020 | 16 | 98 |
| B4176 | Noyep | 2004 | Hulled grains | 2020 | 16 | 94 |
| B4177 | Research | 2004 | Hulled grains | 2020 | 16 | 94 |

| | | | | | | |
|---|---|---|---|---|---|---|
| B4179 | Tibet 1 | 2004 | Hulled grains | 2020 | 16 | 76 |
| B4207 | MGH Rothwell 59175 | 2004 | Hulled grains | 2020 | 16 | 100 |
| B4209 | Fitis | 2004 | Hulled grains | 2020 | 16 | 100 |
| B4210 | Ortolan | 2004 | Hulled grains | 2020 | 16 | 98 |
| B4211 | Ariel*F1(BNP 55*Piroline))9 | 2004 | Hulled grains | 2020 | 16 | 100 |
| B4213 | MGH 60118 | 2004 | Hulled grains | 2020 | 16 | 98 |
| B4215 | Maris Druid | 2004 | Hulled grains | 2020 | 16 | 94 |
| B4228 | NARB 5078 | 2004 | Hulled grains | 2020 | 16 | 100 |
| B4240 | NARB 6251 | 2004 | Hulled grains | 2020 | 16 | 98 |
| B4248 | Hansen 448 latispicatum | 2004 | Hulled grains | 2020 | 16 | 96 |
| B4255 | Hansen 132a2 | 2004 | Hulled grains | 2020 | 16 | 84 |
| B4271 | Hansen 160 transiens | 2004 | Hulled grains | 2020 | 16 | 96 |
| B4341 | Hansen 221 Rehmii | 2004 | Hulled grains | 2020 | 16 | 90 |
| B4355 | Schiemann 8 | 2004 | Hulled grains | 2020 | 16 | 92 |
| B4382 | Betina | 2004 | Hulled grains | 2020 | 16 | 100 |
| B4387 | HB 417/17/4/7 | 2004 | Hulled grains | 2020 | 16 | 92 |
| B4391 | Turkey | 2004 | Hulled grains | 2020 | 16 | 94 |
| B4392 | Turkey | 2004 | Hulled grains | 2020 | 16 | 96 |
| B4399 | Celechovicky Hanacky | 2004 | Hulled grains | 2020 | 16 | 92 |
| B4401 | Semcicky Hospodarsky | 2004 | Hulled grains | 2020 | 16 | 98 |
| B4403 | Slovensky 802 | 2004 | Hulled grains | 2020 | 16 | 90 |
| B4414 | Rajski | 2004 | Hulled grains | 2020 | 16 | 100 |
| B4418 | Sanalta | 2004 | Hulled grains | 2020 | 16 | 96 |
| B4419 | Dorset | 2004 | Hulled grains | 2020 | 16 | 78 |
| B4420 | Nutans 1447 | 2004 | Hulled grains | 2020 | 16 | 94 |
| B4422 | Nutans | 2004 | Hulled grains | 2020 | 16 | 96 |
| B4423 | Nutans | 2004 | Hulled grains | 2020 | 16 | 92 |
| B4424 | Nutans 2 | 2004 | Hulled grains | 2020 | 16 | 90 |
| B4426 | Nutans | 2004 | Hulled grains | 2020 | 16 | 84 |

| | | | | | | |
|---|---|---|---|---|---|---|
| B4445 | Kreta | 2004 | Hulled grains | 2020 | 16 | 98 |
| B4447 | Anatolien | 2004 | Hulled grains | 2020 | 16 | 88 |
| B4449 | Anatolien | 2004 | Hulled grains | 2020 | 16 | 94 |
| B4450 | Anatolien | 2004 | Hulled grains | 2020 | 16 | 94 |
| B4453 | Hindukusch | 2004 | Hulled grains | 2020 | 16 | 96 |
| B4455 | Peloponnes | 2004 | Hulled grains | 2020 | 16 | 100 |
| B4456 | Peloponnes | 2004 | Hulled grains | 2020 | 16 | 94 |
| B4461 | Uruguay | 2004 | Hulled grains | 2020 | 16 | 92 |
| B4463 | Afghan | 2004 | Hulled grains | 2020 | 16 | 98 |
| B4488 | M64-76 | 2004 | Hulled grains | 2020 | 16 | 100 |
| B4493 | Calcu INTA | 2004 | Hulled grains | 2020 | 16 | 100 |
| B4494 | Buck Cruz Del Sur | 2004 | Hulled grains | 2020 | 16 | 98 |
| B4498 | Malteria Heda | 2004 | Hulled grains | 2020 | 16 | 84 |
| B4499 | HB 551/103/2/3/4 | 2004 | Hulled grains | 2020 | 16 | 100 |
| B4501 | H 328 2150 | 2004 | Hulled grains | 2020 | 16 | 76 |
| B4505 | H 286 2154 | 2004 | Hulled grains | 2020 | 16 | 94 |
| B4506 | H 286 2155 | 2004 | Hulled grains | 2020 | 16 | 96 |
| B4508 | H 344 2203 | 2004 | Hulled grains | 2020 | 16 | 96 |
| B4509 | H 350 2209 | 2004 | Hulled grains | 2020 | 16 | 84 |
| B4511 | H 350 2212 | 2004 | Hulled grains | 2020 | 16 | 92 |
| B4512 | H 350 2213 | 2004 | Hulled grains | 2020 | 16 | 100 |
| B4513 | H 350 2214 | 2004 | Hulled grains | 2020 | 16 | 100 |
| B4514 | H 350 2215 | 2004 | Hulled grains | 2020 | 16 | 98 |
| B4516 | H 350 2221 | 2004 | Hulled grains | 2020 | 16 | 96 |
| B4517 | H 351 2222 | 2004 | Hulled grains | 2020 | 16 | 86 |
| B4518 | H 351 2223 | 2004 | Hulled grains | 2020 | 16 | 88 |
| B4519 | H 352 2226 | 2004 | Hulled grains | 2020 | 16 | 92 |
| B4520 | H 316 2229 | 2004 | Hulled grains | 2020 | 16 | 100 |
| B4521 | H 337 2232 | 2004 | Hulled grains | 2020 | 16 | 92 |

| | | | | | | |
|---|---|---|---|---|---|---|
| B4525 | Hellas | 2004 | Hulled grains | 2020 | 16 | 92 |
| B4528 | Malteria 150 | 2004 | Hulled grains | 2020 | 16 | 76 |
| B4532 | Felda | 2004 | Hulled grains | 2020 | 16 | 96 |
| B4540 | Refsum | 2004 | Hulled grains | 2020 | 16 | 98 |
| B4541 | Lauinger Bachtalgerste | 2004 | Hulled grains | 2020 | 16 | 84 |
| B4553 | Afghan K 231 | 2004 | Hulled grains | 2020 | 16 | 80 |
| B4558 | Sejet 51/1732 | 2004 | Hulled grains | 2020 | 16 | 100 |
| B4563 | Erbil | 2004 | Hulled grains | 2020 | 16 | 98 |
| B4564 | Afghan K 228 | 2004 | Hulled grains | 2020 | 16 | 90 |
| B4567 | IHS 13-7-4-4 | 2004 | Hulled grains | 2020 | 16 | 96 |
| B4568 | IHS 13-10-2-3 | 2004 | Hulled grains | 2020 | 16 | 94 |
| B4572 | IHS 17-17-1-4-1 | 2004 | Hulled grains | 2020 | 16 | 98 |
| B4578 | J 70-1 | 2004 | Hulled grains | 2020 | 16 | 90 |
| B4579 | J 70-2 | 2004 | Hulled grains | 2020 | 16 | 92 |
| B4580 | J 70-3 | 2004 | Hulled grains | 2020 | 16 | 98 |
| B4581 | J 70-4 | 2004 | Hulled grains | 2020 | 16 | 92 |
| B4582 | J 70-5 | 2004 | Hulled grains | 2020 | 16 | 100 |
| B4583 | Bonanza | 2004 | Hulled grains | 2020 | 16 | 98 |
| B4585 | Ethiopia CI 5791 | 2004 | Hulled grains | 2020 | 16 | 94 |
| B4598 | HB 419/66/1/4 | 2004 | Hulled grains | 2020 | 16 | 100 |
| B4599 | HB 414/2/2/3/3 | 2004 | Hulled grains | 2020 | 16 | 98 |
| B4600 | HB 769/10/8 | 2004 | Hulled grains | 2020 | 16 | 100 |
| B4601 | Crusader | 2004 | Hulled grains | 2020 | 16 | 96 |
| B4602 | Feronia | 2004 | Hulled grains | 2020 | 16 | 100 |
| B4606 | Convaritat Labile | 2004 | Hulled grains | 2020 | 16 | 90 |
| B4607 | Convaritat Labile | 2004 | Hulled grains | 2020 | 16 | 76 |
| B4609 | Convaritat Labile | 2004 | Hulled grains | 2020 | 16 | 74 |
| B4610 | Convaritat Labile | 2004 | Hulled grains | 2020 | 16 | 64 |
| B4611 | HB 346 | 2004 | Hulled grains | 2020 | 16 | 86 |

| | | | | | | |
|---|---|---|---|---|---|---|
| B4623 | HB 551/29/102/3 | 2004 | Hulled grains | 2020 | 16 | 98 |
| B4624 | HB 551/48/19/5/3 | 2004 | Hulled grains | 2020 | 16 | 90 |
| B4625 | HB 551/48/26/11 | 2004 | Hulled grains | 2020 | 16 | 100 |
| B4632 | C 177 | 2004 | Hulled grains | 2020 | 16 | 90 |
| B4633 | B 2145 | 2004 | Hulled grains | 2020 | 16 | 80 |
| B4640 | Gipsy | 2004 | Hulled grains | 2020 | 16 | 94 |
| B4645 | Tunesian | 2004 | Hulled grains | 2020 | 16 | 90 |
| B4646 | Arma | 2004 | Hulled grains | 2020 | 16 | 76 |
| B4648 | Ciro | 2004 | Hulled grains | 2020 | 16 | 78 |
| B4649 | Dans Irisaka 8-31 | 2004 | Hulled grains | 2020 | 16 | 72 |
| B4653 | Nemcinovskij | 2004 | Hulled grains | 2020 | 16 | 100 |
| B4677 | Universe | 2004 | Hulled grains | 2020 | 16 | 94 |
| B4688 | Wisconsin H 35-7-2-1-3 | 2004 | Hulled grains | 2020 | 16 | 92 |
| B4690 | Mimi | 2004 | Hulled grains | 2020 | 16 | 98 |
| B4693 | Pryor | 2004 | Hulled grains | 2020 | 16 | 74 |
| B4694 | Sagres | 2004 | Hulled grains | 2020 | 16 | 98 |
| B4786 | Nepal 102A BC-1 | 2004 | Hulled grains | 2020 | 16 | 94 |
| B5088 | Nepal 21A BC-1 | 2004 | Hulled grains | 2020 | 16 | 94 |
| B5092 | Nepal 31A BC-1 | 2004 | Hulled grains | 2020 | 16 | 100 |
| B5093 | Nepal 34A BC-1 | 2004 | Hulled grains | 2020 | 16 | 100 |
| B5097 | Nepal 41A BC-1 | 2004 | Hulled grains | 2020 | 16 | 98 |
| B5098 | Nepal 48A BC-1 | 2004 | Hulled grains | 2020 | 16 | 96 |
| B5100 | Nepal 57B BC-1 | 2004 | Hulled grains | 2020 | 16 | 92 |
| B5101 | Nepal 58A BC-1 | 2004 | Hulled grains | 2020 | 16 | 94 |
| B5105 | Nepal 65C BC-1 | 2004 | Hulled grains | 2020 | 16 | 98 |
| B5106 | Nepal 66A BC-1 | 2004 | Hulled grains | 2020 | 16 | 98 |
| B5107 | Nepal 71A BC-1 | 2004 | Hulled grains | 2020 | 16 | 90 |
| B5108 | Nepal 72A BC-1 | 2004 | Hulled grains | 2020 | 16 | 100 |
| B5110 | Nepal 73A BC-1 | 2004 | Hulled grains | 2020 | 16 | 94 |

| | | | | | | |
|---|---|---|---|---|---|---|
| B5113 | Nepal 83A BC-1 | 2004 | Hulled grains | 2020 | 16 | 78 |
| B5114 | Nepal 85A BC-1 | 2004 | Hulled grains | 2020 | 16 | 94 |
| B5115 | Nepal 88A BN-1 | 2004 | Hulled grains | 2020 | 16 | 100 |
| B5118 | Nepal 97A BC-1 | 2004 | Hulled grains | 2020 | 16 | 78 |
| B3504 | Kitami Hadaka | 2004 | Hulless | 2020 | 16 | 94 |
| B3507 | Kherson | 2004 | Hulless | 2020 | 16 | 52 |
| B3682 | Mestny | 2004 | Hulless | 2020 | 16 | 66 |
| B3699 | Sagatairyu 2 | 2004 | Hulless | 2020 | 16 | 82 |
| B3970 | Juk Ha | 2004 | Hulless | 2020 | 16 | 94 |
| B3974 | Wanju | 2004 | Hulless | 2020 | 16 | 90 |
| B4164 | Anguillara | 2004 | Hulless | 2020 | 16 | 46 |
| B4165 | Esame | 2004 | Hulless | 2020 | 16 | 64 |
| B4168 | Espresso | 2004 | Hulless | 2020 | 16 | 98 |
| B4172 | Prode | 2004 | Hulless | 2020 | 16 | 32 |
| B4178 | Hulless Barley | 2004 | Hulless | 2020 | 16 | 22 |
| B4185 | Tibet 7 | 2004 | Hulless | 2020 | 16 | 68 |
| B4186 | Tibet 8 | 2004 | Hulless | 2020 | 16 | 40 |
| B4187 | Tibet 9 | 2004 | Hulless | 2020 | 16 | 32 |
| B4188 | Tibet 10 | 2004 | Hulless | 2020 | 16 | 50 |
| B4192 | Tibet 14 | 2004 | Hulless | 2020 | 16 | 78 |
| B4193 | Tibet 15 | 2004 | Hulless | 2020 | 16 | 70 |
| B4194 | Tibet 16 | 2004 | Hulless | 2020 | 16 | 90 |
| B4195 | Tibet 17 | 2004 | Hulless | 2020 | 16 | 86 |
| B4197 | Tibet 21 | 2004 | Hulless | 2020 | 16 | 80 |
| B4199 | Tibet 23 | 2004 | Hulless | 2020 | 16 | 96 |
| B4200 | Tibet 35 | 2004 | Hulless | 2020 | 16 | 96 |
| B4202 | Tibet 38 | 2004 | Hulless | 2020 | 16 | 68 |
| B4204 | Tibet 40 | 2004 | Hulless | 2020 | 16 | 96 |
| B4219 | Tibet 42 | 2004 | Hulless | 2020 | 16 | 82 |

| | | | | | | |
|---|---|---|---|---|---|---|
| B4221 | Tibet 13299/3 | 2004 | Hulless | 2020 | 16 | 98 |
| B4223 | Tibet 13299/K | 2004 | Hulless | 2020 | 16 | 100 |
| B4224 | Tibet 13299/M | 2004 | Hulless | 2020 | 16 | 88 |
| B4244 | NARB 6096 | 2004 | Hulless | 2020 | 16 | 100 |
| B4283 | Hansen 236 | 2004 | Hulless | 2020 | 16 | 100 |
| B4351 | Schiemann 3 | 2004 | Hulless | 2020 | 16 | 100 |
| B4359 | Sel Gat | 2004 | Hulless | 2020 | 16 | 96 |
| B4415 | Buff | 2004 | Hulless | 2020 | 16 | 72 |
| B4416 | Columbische Nackte | 2004 | Hulless | 2020 | 16 | 68 |
| B4438 | Tibet | 2004 | Hulless | 2020 | 16 | 82 |
| B4462 | Mittelamerika | 2004 | Hulless | 2020 | 16 | 76 |
| B4679 | Von Rumkers 3367/31622 | 2004 | Hulless | 2020 | 16 | 30 |
| B4681 | Von Rumkers 3657/523811 | 2004 | Hulless | 2020 | 16 | 40 |
| B4684 | Von Rumkers 4454/51942 | 2004 | Hulless | 2020 | 16 | 28 |
| B4685 | Von Rumkers 3477 | 2004 | Hulless | 2020 | 16 | 40 |
| B4686 | Anca | 2004 | Hulless | 2020 | 16 | 70 |
| B5095 | Nepal 37A BN-1 | 2004 | Hulless | 2020 | 16 | 48 |
| B5109 | Nepal 72B BN-1 | 2004 | Hulless | 2020 | 16 | 22 |
| B3533 | Nordost | 2003 | Hulled grains | 2020 | 17 | 94 |
| B3548 | Otterbacher | 2003 | Hulled grains | 2020 | 17 | 74 |
| B3681 | Mestny | 2003 | Hulled grains | 2020 | 17 | 92 |
| B3698 | Aizu 4 | 2003 | Hulled grains | 2020 | 17 | 90 |
| B3828 | Ethiopia R 460 | 2003 | Hulled grains | 2020 | 17 | 74 |
| B3959 | Conquest | 2003 | Hulled grains | 2020 | 17 | 90 |
| B3973 | Baik Daik | 2003 | Hulled grains | 2020 | 17 | 86 |
| B3975 | Baik Maik | 2003 | Hulled grains | 2020 | 17 | 100 |
| B3976 | Harrison | 2003 | Hulled grains | 2020 | 17 | 100 |
| B3999 | Martonvasari FB 104 | 2003 | Hulled grains | 2020 | 17 | 96 |
| B4002 | Aizu 7 | 2003 | Hulled grains | 2020 | 17 | 78 |

| | | | | | | |
|---|---|---|---|---|---|---|
| B4017 | Israeli 264 | 2003 | Hulled grains | 2020 | 17 | 98 |
| B4019 | Omer (B5-58) | 2003 | Hulled grains | 2020 | 17 | 84 |
| B4119 | Rogers | 2003 | Hulled grains | 2020 | 17 | 96 |
| B4149 | Tammi | 2003 | Hulled grains | 2020 | 17 | 82 |
| B4150 | Otra | 2003 | Hulled grains | 2020 | 17 | 88 |
| B4243 | NARB 6555 | 2003 | Hulled grains | 2020 | 17 | 86 |
| B4421 | Nutans 3006 | 2003 | Hulled grains | 2020 | 17 | 98 |
| B4431 | 62-420-11 | 2003 | Hulled grains | 2020 | 17 | 96 |
| B4432 | Coho | 2003 | Hulled grains | 2020 | 17 | 92 |
| B4486 | Maris Baldric | 2003 | Hulled grains | 2020 | 17 | 94 |
| B4490 | M65-94 | 2003 | Hulled grains | 2020 | 17 | 94 |
| B4492 | Negra Manfredi MAG | 2003 | Hulled grains | 2020 | 17 | 94 |
| B4496 | Magnif 102 Inta | 2003 | Hulled grains | 2020 | 17 | 82 |
| B4502 | H 285 2151 | 2003 | Hulled grains | 2020 | 17 | 74 |
| B4504 | H 286 2153 | 2003 | Hulled grains | 2020 | 17 | 88 |
| B4507 | H 279 2176 | 2003 | Hulled grains | 2020 | 17 | 62 |
| B4510 | H 350 2211 | 2003 | Hulled grains | 2020 | 17 | 94 |
| B4515 | H 350 2220 | 2003 | Hulled grains | 2020 | 17 | 98 |
| B4524 | Kristina | 2003 | Hulled grains | 2020 | 17 | 94 |
| B4539 | Donnes | 2003 | Hulled grains | 2020 | 17 | 90 |
| B4544 | Modia | 2003 | Hulled grains | 2020 | 17 | 70 |
| B4559 | Ariana | 2003 | Hulled grains | 2020 | 17 | 98 |
| B4561 | J 20 | 2003 | Hulled grains | 2020 | 17 | 94 |
| B4573 | IHS 17-17-3-2-2 | 2003 | Hulled grains | 2020 | 17 | 96 |
| B4575 | IHS 19-18-2-2 | 2003 | Hulled grains | 2020 | 17 | 94 |
| B4576 | IHS 29-19-2-1-3 | 2003 | Hulled grains | 2020 | 17 | 100 |
| B4631 | Maris Dingo | 2003 | Hulled grains | 2020 | 17 | 92 |
| B4661 | Dornburger 4-Z Futtergerste | 2003 | Hulled grains | 2020 | 17 | 90 |
| B4687 | Drake | 2003 | Hulled grains | 2020 | 17 | 86 |

| | | | | | | |
|---|---|---|---|---|---|---|
| B4695 | MGH 6271-7 | 2003 | Hulled grains | 2020 | 17 | 96 |
| B5090 | Nepal 25A BC-1 | 2003 | Hulled grains | 2020 | 17 | 96 |
| B5094 | Nepal 37A BC-1 | 2003 | Hulled grains | 2020 | 17 | 94 |
| B5103 | Nepal 61A BC-1 | 2003 | Hulled grains | 2020 | 17 | 98 |
| B5104 | Nepal 62A BC-1 | 2003 | Hulled grains | 2020 | 17 | 90 |
| B5120 | Nepal 103B BC-1 | 2003 | Hulled grains | 2020 | 17 | 94 |
| B5703 | Nepal 103B BC-5 | 2003 | Hulled grains | 2020 | 17 | 98 |
| B18333 | WID-104 | 2003 | Hulled grains | 2020 | 17 | 48 |
| B3540 | 13 Novi Sad | 2003 | Hulless | 2020 | 17 | 26 |
| B4169 | Lecce | 2003 | Hulless | 2020 | 17 | 22 |
| B4170 | Leonessa | 2003 | Hulless | 2020 | 17 | 24 |
| B4409 | Ethiopie Ab 1105 | 2003 | Hulless | 2020 | 17 | 40 |
| B4410 | Dometzkoer Paradies | 2003 | Hulless | 2020 | 17 | 44 |
| B4412 | Michalovicky Nahy | 2003 | Hulless | 2020 | 17 | 32 |
| B4682 | Von Rumkers 3657/52382 | 2003 | Hulless | 2020 | 17 | 32 |
| B4683 | Von Rumkers 4084/50532 | 2003 | Hulless | 2020 | 17 | 28 |
| B5091 | Nepal 25A BN-1 | 2003 | Hulless | 2020 | 17 | 78 |
| B5119 | Nepal 103A BC-1 | 2003 | Hulless | 2020 | 17 | 76 |
| B5695 | Nepal 97A BC-6 | 2003 | Hulless | 2020 | 17 | 60 |
| B17231 | PF 5368 | 1978 | Hulled grains | 2019 | 41 | 96 |
| B17232 | PF 5801 | 1978 | Hulled grains | 2019 | 41 | 62 |
| B17234 | MG 4127-5 | 1978 | Hulled grains | 2019 | 41 | 60 |
| B17235 | Cebeco 7524 | 1978 | Hulled grains | 2019 | 41 | 82 |
| B17295 | HJ 248/22/672 | 1978 | Hulled grains | 2019 | 41 | 94 |
| B17296 | HW 48/73/15/4 | 1978 | Hulled grains | 2019 | 41 | 88 |
| B17297 | HJ 462/10/1569 | 1978 | Hulled grains | 2019 | 41 | 86 |
| B17298 | HW 48/42/1/5 | 1978 | Hulled grains | 2019 | 41 | 70 |
| B17299 | HJ 193/3/1488 | 1978 | Hulled grains | 2019 | 41 | 52 |
| B18208 | Iran 1 | 1978 | Hulled grains | 2019 | 41 | 86 |

| | | | | | | |
|---|---|---|---|---|---|---|
| B18209 | Iran 2 | 1978 | Hulled grains | 2019 | 41 | 46 |
| B18210 | Iran 3 | 1978 | Hulled grains | 2019 | 41 | 34 |
| B18212 | Iran 5 | 1978 | Hulled grains | 2019 | 41 | 90 |
| B3565 | Pereszteg i 3 | 1978 | Hulled grains | 2019 | 41 | 70 |
| B3745 | Spontaneum spontaneum | 1978 | Hulled grains | 2019 | 41 | 68 |
| B3802 | Ethiopia R 281 | 1978 | Hulled grains | 2019 | 41 | 32 |
| B3864 | Ethiopia R 159 | 1978 | Hulled grains | 2019 | 41 | 36 |
| B4569 | IHS 13-13-6-3-1 | 1978 | Hulled grains | 2019 | 41 | 68 |
| B4912 | Decorticatum | 1978 | Hulled grains | 2019 | 41 | 24 |
| B5021 | BGM 51 Short Straw | 1978 | Hulled grains | 2019 | 41 | 66 |
| B5024 | 63/1140 R/582 | 1978 | Hulled grains | 2019 | 41 | 48 |
| B5029 | Third Outer Glume trd | 1978 | Hulled grains | 2019 | 41 | 24 |
| B5034 | 63 Ab 5401 53/0/16 | 1978 | Hulled grains | 2019 | 41 | 10 |
| B5036 | 63 Ab 5397 53/0/24 | 1978 | Hulled grains | 2019 | 41 | 40 |
| B5346 | Uniculm Compana (Kindred) | 1978 | Hulled grains | 2019 | 41 | 4 |
| B5442 | Notch 1 | 1978 | Hulled grains | 2019 | 41 | 60 |
| B5726 | E 9149 BV-75 | 1978 | Hulled grains | 2019 | 41 | 2 |
| B5949 | HB 848/48/23/106 | 1978 | Hulled grains | 2019 | 41 | 84 |
| B5956 | NE 72637 | 1978 | Hulled grains | 2019 | 41 | 96 |
| B6003 | UCD 76-104 | 1978 | Hulled grains | 2019 | 41 | 36 |
| B6011 | UCD 72-61-4 | 1978 | Hulled grains | 2019 | 41 | 62 |
| B6015 | UCD 75-10 | 1978 | Hulled grains | 2019 | 41 | 58 |
| B6016 | UCD 75-12 | 1978 | Hulled grains | 2019 | 41 | 66 |
| B6060 | HO 465-1 | 1978 | Hulled grains | 2019 | 41 | 62 |
| B6085 | H 150 | 1978 | Hulled grains | 2019 | 41 | 36 |
| B6087 | BE 734123 | 1978 | Hulled grains | 2019 | 41 | 70 |
| B6129 | OSB 70028 | 1978 | Hulled grains | 2019 | 41 | 16 |
| B6194 | HCB 27/1/14 | 1978 | Hulled grains | 2019 | 41 | 70 |
| B6377 | Riso Mutant 7 | 1978 | Hulled grains | 2019 | 41 | 70 |

| | | | | | | |
|---|---|---|---|---|---|---|
| B6378 | Riso Mutant 56 | 1978 | Hulled grains | 2019 | 41 | 64 |
| B6380 | HLM 449 | 1978 | Hulled grains | 2019 | 41 | 24 |
| B6381 | BGM 62 | 1978 | Hulled grains | 2019 | 41 | 44 |
| B7562 | Translocation T3 7A | 1978 | Hulled grains | 2019 | 41 | 62 |
| B7832 | Afghan R 938 | 1978 | Hulled grains | 2019 | 41 | 6 |
| B8102 | MC 5540 | 1978 | Hulled grains | 2019 | 41 | 30 |
| B8282 | CBHN(66)7/106(H) | 1978 | Hulled grains | 2019 | 41 | 76 |
| B8283 | CBRD(66)7/618C | 1978 | Hulled grains | 2019 | 41 | 50 |
| B9249 | GAW 35-1 | 1978 | Hulled grains | 2019 | 41 | 0 |
| B9256 | GAW 42-1 | 1978 | Hulled grains | 2019 | 41 | 0 |
| B9261 | GAW 44-5 | 1978 | Hulled grains | 2019 | 41 | 0 |
| B9394 | GAW 82-3 | 1978 | Hulled grains | 2019 | 41 | 0 |
| B9599 | Hembar * Beecher 10L | 1978 | Hulled grains | 2019 | 41 | 0 |
| B9850 | South America 2.33 | 1978 | Hulled grains | 2019 | 41 | 38 |
| B9868 | South America 6.38 | 1978 | Hulled grains | 2019 | 41 | 2 |
| B9890 | South America 6.243 | 1978 | Hulled grains | 2019 | 41 | 2 |
| B4286 | Hansen 534 erectonudum | 1978 | Hulless | 2019 | 41 | 0 |
| B5041 | Chlorina Seedling fc | 1978 | Hulless | 2019 | 41 | 0 |
| B5053 | Albino Seedling Anan | 1978 | Hulless | 2019 | 41 | 0 |
| B6382 | Uzu (Semi-brachytic) uz | 1978 | Hulless | 2019 | 41 | 10 |
| B6383 | Xantha Seedling Xnxn | 1978 | Hulless | 2019 | 41 | 12 |
| B16955 | Pakistan 75A BC-3 | 1977 | Hulled grains | 2019 | 42 | 28 |
| B16956 | Pakistan 75A BC-4 | 1977 | Hulled grains | 2019 | 42 | 16 |
| B16960 | Pakistan 75A BC-8 | 1977 | Hulled grains | 2019 | 42 | 50 |
| B16979 | Pakistan 76A BN-7 | 1977 | Hulled grains | 2019 | 42 | 18 |
| B16981 | Pakistan 76A BN-9 | 1977 | Hulled grains | 2019 | 42 | 42 |
| B17094 | Pakistan 90A BC-6 | 1977 | Hulled grains | 2019 | 42 | 8 |
| B17102 | Pakistan 90A BC-14 | 1977 | Hulled grains | 2019 | 42 | 0 |
| B17103 | Pakistan 90A BC-15 | 1977 | Hulled grains | 2019 | 42 | 22 |

| | | | | | | |
|---|---|---|---|---|---|---|
| B17122 | Pakistan 91 BC-5 | 1977 | Hulled grains | 2019 | 42 | 10 |
| B17130 | Pakistan 92 BC-8 | 1977 | Hulled grains | 2019 | 42 | 8 |
| B17150 | Pakistan 94 BC-8 | 1977 | Hulled grains | 2019 | 42 | 6 |
| B17158 | Pakistan 95 BC-6 | 1977 | Hulled grains | 2019 | 42 | 0 |
| B17159 | Pakistan 95 BC-7 | 1977 | Hulled grains | 2019 | 42 | 20 |
| B17168 | Pakistan 96 BC-8 | 1977 | Hulled grains | 2019 | 42 | 0 |
| B17170 | Pakistan 96 BC-10 | 1977 | Hulled grains | 2019 | 42 | 4 |
| B17180 | Pakistan 97 BC-5 | 1977 | Hulled grains | 2019 | 42 | 10 |
| B17183 | Pakistan 97 BC-8 | 1977 | Hulled grains | 2019 | 42 | 6 |
| B17190 | Pakistan 98 BC-3 | 1977 | Hulled grains | 2019 | 42 | 6 |
| B17194 | Pakistan 98 BC-7 | 1977 | Hulled grains | 2019 | 42 | 8 |
| B17199 | Pakistan 99 BC-2 | 1977 | Hulled grains | 2019 | 42 | 26 |
| B17201 | Pakistan 99 BC-4 | 1977 | Hulled grains | 2019 | 42 | 10 |
| B17206 | Pakistan 99 BC-9 | 1977 | Hulled grains | 2019 | 42 | 16 |
| B17218 | Pakistan 100 BC-9 | 1977 | Hulled grains | 2019 | 42 | 26 |
| B17222 | Pakistan 101 BC-2 | 1977 | Hulled grains | 2019 | 42 | 10 |
| B17228 | Pakistan 101 BC-8 | 1977 | Hulled grains | 2019 | 42 | 12 |
| B5862 | Welgevallen 521-3-1A | 1977 | Hulled grains | 2019 | 42 | 94 |
| B5914 | Welgevallen 46/76-1-5 | 1977 | Hulled grains | 2019 | 42 | 80 |
| B6511 | Pakistan 31A BN-2 | 1977 | Hulled grains | 2019 | 42 | 6 |
| B6617 | Pakistan 38A BC-7 | 1977 | Hulled grains | 2019 | 42 | 6 |
| B6623 | Pakistan 38A BC-13 | 1977 | Hulled grains | 2019 | 42 | 0 |
| B6650 | Pakistan 39 BC-11 | 1977 | Hulled grains | 2019 | 42 | 4 |
| B7417 | Trifurcated | 1977 | Hulled grains | 2019 | 42 | 0 |
| B16677 | Pakistan 42 BN-3 | 1977 | Hulless | 2019 | 42 | 10 |
| B16680 | Pakistan 42 BN-6 | 1977 | Hulless | 2019 | 42 | 2 |
| B16701 | Pakistan 45 BN-12 | 1977 | Hulless | 2019 | 42 | 12 |
| B16708 | Pakistan 46 BN-7 | 1977 | Hulless | 2019 | 42 | 36 |
| B16715 | Pakistan 47 BN-4 | 1977 | Hulless | 2019 | 42 | 32 |

| | | | | | | |
|---|---|---|---|---|---|---|
| B16722 | Pakistan 47 BN-11 | 1977 | Hulless | 2019 | 42 | 26 |
| B16740 | Pakistan 49 BN-3 | 1977 | Hulless | 2019 | 42 | 2 |
| B16741 | Pakistan 49 BN-4 | 1977 | Hulless | 2019 | 42 | 0 |
| B16743 | Pakistan 49 BN-6 | 1977 | Hulless | 2019 | 42 | 2 |
| B16758 | Pakistan 51 BN-2 | 1977 | Hulless | 2019 | 42 | 30 |
| B16760 | Pakistan 51 BN-4 | 1977 | Hulless | 2019 | 42 | 16 |
| B16762 | Pakistan 51 BN-6 | 1977 | Hulless | 2019 | 42 | 32 |
| B16806 | Pakistan 56 BN-6 | 1977 | Hulless | 2019 | 42 | 18 |
| B16812 | Pakistan 56 BN-12 | 1977 | Hulless | 2019 | 42 | 0 |
| B16847 | Pakistan 63 BN-5 | 1977 | Hulless | 2019 | 42 | 8 |
| B16852 | Pakistan 63 BN-10 | 1977 | Hulless | 2019 | 42 | 14 |
| B16855 | Pakistan 64 BN-2 | 1977 | Hulless | 2019 | 42 | 8 |
| B16864 | Pakistan 66 BN-3 | 1977 | Hulless | 2019 | 42 | 50 |
| B16866 | Pakistan 66 BN-5 | 1977 | Hulless | 2019 | 42 | 6 |
| B16873 | Pakistan 67 BN-4 | 1977 | Hulless | 2019 | 42 | 12 |
| B16877 | Pakistan 67 BN-8 | 1977 | Hulless | 2019 | 42 | 16 |
| B16881 | Pakistan 68 BN-3 | 1977 | Hulless | 2019 | 42 | 10 |
| B16894 | Pakistan 71A BC-2 | 1977 | Hulless | 2019 | 42 | 16 |
| B16914 | Pakistan 72B BN-7 | 1977 | Hulless | 2019 | 42 | 10 |
| B16921 | Pakistan 73 BN-3 | 1977 | Hulless | 2019 | 42 | 0 |
| B16925 | Pakistan 73 BN-7 | 1977 | Hulless | 2019 | 42 | 0 |
| B16941 | Pakistan 74B BN-5 | 1977 | Hulless | 2019 | 42 | 14 |
| B16961 | Pakistan 75B BN-1 | 1977 | Hulless | 2019 | 42 | 8 |
| B16963 | Pakistan 75B BN-3 | 1977 | Hulless | 2019 | 42 | 14 |
| B16995 | Pakistan 76B BN-10 | 1977 | Hulless | 2019 | 42 | 2 |
| B17001 | Pakistan 77 BN-3 | 1977 | Hulless | 2019 | 42 | 6 |
| B17006 | Pakistan 77 BN-8 | 1977 | Hulless | 2019 | 42 | 12 |
| B17015 | Pakistan 78 BN-7 | 1977 | Hulless | 2019 | 42 | 0 |
| B17019 | Pakistan 79 BN-2 | 1977 | Hulless | 2019 | 42 | 0 |

| | | | | | | |
|---|---|---|---|---|---|---|
| B17029 | Pakistan 80 BN-6 | 1977 | Hulless | 2019 | 42 | 0 |
| B17030 | Pakistan 80 BN-7 | 1977 | Hulless | 2019 | 42 | 2 |
| B17036 | Pakistan 81 BN-3 | 1977 | Hulless | 2019 | 42 | 0 |
| B17046 | Pakistan 82 BN-3 | 1977 | Hulless | 2019 | 42 | 0 |
| B17061 | Pakistan 84 BN-4 | 1977 | Hulless | 2019 | 42 | 16 |
| B17064 | Pakistan 84 BN-7 | 1977 | Hulless | 2019 | 42 | 4 |
| B17065 | Pakistan 84 BN-8 | 1977 | Hulless | 2019 | 42 | 2 |
| B17067 | Pakistan 84 BN-10 | 1977 | Hulless | 2019 | 42 | 2 |
| B17068 | Pakistan 84 BN-11 | 1977 | Hulless | 2019 | 42 | 0 |
| B17073 | Pakistan 85 BN-4 | 1977 | Hulless | 2019 | 42 | 0 |
| B17074 | Pakistan 85 BN-5 | 1977 | Hulless | 2019 | 42 | 0 |
| B17104 | Pakistan 90B BN-1 | 1977 | Hulless | 2019 | 42 | 2 |
| B17109 | Pakistan 90B BN-6 | 1977 | Hulless | 2019 | 42 | 0 |
| B6394 | Pakistan 6 BN-5 | 1977 | Hulless | 2019 | 42 | 16 |
| B6397 | Pakistan 6 BN-8 | 1977 | Hulless | 2019 | 42 | 10 |
| B6399 | Pakistan 6 BN-10 | 1977 | Hulless | 2019 | 42 | 6 |
| B6414 | Pakistan 15 BN-3 | 1977 | Hulless | 2019 | 42 | 8 |
| B6417 | Pakistan 15 BN-6 | 1977 | Hulless | 2019 | 42 | 2 |
| B6418 | Pakistan 15 BN-7 | 1977 | Hulless | 2019 | 42 | 0 |
| B6456 | Pakistan 24 BN-7 | 1977 | Hulless | 2019 | 42 | 28 |
| B6460 | Pakistan 25 BN-2 | 1977 | Hulless | 2019 | 42 | 0 |
| B6476 | Pakistan 27 BN-1 | 1977 | Hulless | 2019 | 42 | 0 |
| B6497 | Pakistan 29 BN-5 | 1977 | Hulless | 2019 | 42 | 40 |
| B6531 | Pakistan 31B BN-11 | 1977 | Hulless | 2019 | 42 | 0 |
| B6540 | Pakistan 32A BN-5 | 1977 | Hulless | 2019 | 42 | 2 |
| B6547 | Pakistan 32A BN-12 | 1977 | Hulless | 2019 | 42 | 30 |
| B6584 | Pakistan 35 BN-6 | 1977 | Hulless | 2019 | 42 | 24 |
| B6592 | Pakistan 36 BN-5 | 1977 | Hulless | 2019 | 42 | 44 |
| B6595 | Pakistan 36 BN-8 | 1977 | Hulless | 2019 | 42 | 20 |

| | | | | | | |
|---|---|---|---|---|---|---|
| B6596 | Pakistan 36 BN-9 | 1977 | Hulless | 2019 | 42 | 42 |
| B6598 | Pakistan 37 BN-2 | 1977 | Hulless | 2019 | 42 | 16 |
| B6627 | Pakistan 38B BN-4 | 1977 | Hulless | 2019 | 42 | 48 |
| B6649 | Pakistan 39 BC-10 | 1977 | Hulless | 2019 | 42 | 2 |
| B6652 | Pakistan 39 BC-13 | 1977 | Hulless | 2019 | 42 | 2 |
| B4715 | Nepal 8A BC-1 | 1976 | Hulled grains | 2019 | 43 | 82 |
| B4730 | Nepal 32A BC-1 | 1976 | Hulled grains | 2019 | 43 | 88 |
| B4732 | Nepal 35A BC-1 | 1976 | Hulled grains | 2019 | 43 | 80 |
| B4734 | Nepal 38A BC-1 | 1976 | Hulled grains | 2019 | 43 | 100 |
| B4740 | Nepal 49A BC-1 | 1976 | Hulled grains | 2019 | 43 | 100 |
| B4752 | Nepal 60A BC-1 | 1976 | Hulled grains | 2019 | 43 | 92 |
| B4758 | Nepal 65B BC-1 | 1976 | Hulled grains | 2019 | 43 | 92 |
| B4776 | Nepal 92A BC-1 | 1976 | Hulled grains | 2019 | 43 | 84 |
| B4887 | H-14 | 1976 | Hulled grains | 2019 | 43 | 82 |
| B5076 | HB 797/72/5/7 | 1976 | Hulled grains | 2019 | 43 | 86 |
| B5078 | HB 797/92/7/3 | 1976 | Hulled grains | 2019 | 43 | 68 |
| B5083 | Nepal 3A BC-1 | 1976 | Hulled grains | 2019 | 43 | 88 |
| B5084 | Nepal 4A BC-1 | 1976 | Hulled grains | 2019 | 43 | 80 |
| B5180 | Welgevallen 60/14-23 | 1976 | Hulled grains | 2019 | 43 | 68 |
| B5181 | Welgevallen 60/21-35 | 1976 | Hulled grains | 2019 | 43 | 60 |
| B5191 | Welgevallen 60/14-166 | 1976 | Hulled grains | 2019 | 43 | 86 |
| B5199 | Welgevallen 62/13-113 | 1976 | Hulled grains | 2019 | 43 | 96 |
| B5203 | Welgevallen 60/17-2-16 | 1976 | Hulled grains | 2019 | 43 | 58 |
| B5212 | Welgevallen 65/30-9 | 1976 | Hulled grains | 2019 | 43 | 100 |
| B5216 | Welgevallen 65/31-101 | 1976 | Hulled grains | 2019 | 43 | 88 |
| B5218 | Welgevallen 60/19-49 | 1976 | Hulled grains | 2019 | 43 | 96 |
| B5224 | Welgevallen 65/4-1 | 1976 | Hulled grains | 2019 | 43 | 68 |
| B5235 | Welgevallen 62/13-6 | 1976 | Hulled grains | 2019 | 43 | 82 |
| B5240 | Welgevallen 65/5-4 | 1976 | Hulled grains | 2019 | 43 | 34 |

| | | | | | | |
|---|---|---|---|---|---|---|
| B5248 | Welgevallen 60/19-1-5 | 1976 | Hulled grains | 2019 | 43 | 70 |
| B5250 | Welgevallen 62/13-48 | 1976 | Hulled grains | 2019 | 43 | 42 |
| B5254 | Welgevallen 65/4-6 | 1976 | Hulled grains | 2019 | 43 | 72 |
| B5259 | Welgevallen 65/43-4 | 1976 | Hulled grains | 2019 | 43 | 78 |
| B5262 | Welgevallen 60/A-3-11 | 1976 | Hulled grains | 2019 | 43 | 84 |
| B5271 | Welgevallen 65/31-97 | 1976 | Hulled grains | 2019 | 43 | 98 |
| B5289 | Welgevallen 62/13-88 | 1976 | Hulled grains | 2019 | 43 | 36 |
| B5290 | Welgevallen 62/13-41 | 1976 | Hulled grains | 2019 | 43 | 42 |
| B5291 | Welgevallen 60/19-59 | 1976 | Hulled grains | 2019 | 43 | 52 |
| B5295 | Welgevallen 62/19-2-15 | 1976 | Hulled grains | 2019 | 43 | 70 |
| B5303 | HJ 34/55/226/6/10 | 1976 | Hulled grains | 2019 | 43 | 92 |
| B5307 | HW 10/1166/9 | 1976 | Hulled grains | 2019 | 43 | 88 |
| B5319 | Minnesota 70-2 | 1976 | Hulled grains | 2019 | 43 | 96 |
| B5324 | Minnesota 92-43 | 1976 | Hulled grains | 2019 | 43 | 98 |
| B5348 | HB 822/6/4 | 1976 | Hulled grains | 2019 | 43 | 70 |
| B5438 | Multinodal Dwarf 398 | 1976 | Hulled grains | 2019 | 43 | 72 |
| B5450 | Nepal 12B BC-3 | 1976 | Hulled grains | 2019 | 43 | 78 |
| B5451 | Nepal 12B BC-4 | 1976 | Hulled grains | 2019 | 43 | 66 |
| B5452 | Nepal 15A BN-2 | 1976 | Hulled grains | 2019 | 43 | 92 |
| B5462 | Nepal 27A BC-3 | 1976 | Hulled grains | 2019 | 43 | 56 |
| B5463 | Nepal 27A BC-4 | 1976 | Hulled grains | 2019 | 43 | 56 |
| B5473 | Nepal 36A BC-3 | 1976 | Hulled grains | 2019 | 43 | 96 |
| B5479 | Nepal 43A BC-2 | 1976 | Hulled grains | 2019 | 43 | 80 |
| B5483 | Nepal 44A BC-3 | 1976 | Hulled grains | 2019 | 43 | 84 |
| B5488 | Nepal 50A BC-4 | 1976 | Hulled grains | 2019 | 43 | 96 |
| B5492 | Nepal 52A BC-2 | 1976 | Hulled grains | 2019 | 43 | 54 |
| B5495 | Nepal 54A BC-3 | 1976 | Hulled grains | 2019 | 43 | 56 |
| B5504 | Nepal 59A BC-4 | 1976 | Hulled grains | 2019 | 43 | 96 |
| B5506 | Nepal 59A BC-6 | 1976 | Hulled grains | 2019 | 43 | 86 |

| | | | | | | |
|---|---|---|---|---|---|---|
| B5507 | Nepal 60A BC-2 | 1976 | Hulled grains | 2019 | 43 | 98 |
| B5513 | Nepal 60A BC-8 | 1976 | Hulled grains | 2019 | 43 | 70 |
| B5518 | Nepal 61A BC-6 | 1976 | Hulled grains | 2019 | 43 | 96 |
| B5519 | Nepal 61A BC-7 | 1976 | Hulled grains | 2019 | 43 | 88 |
| B5541 | Nepal 81A BN-5 | 1976 | Hulled grains | 2019 | 43 | 78 |
| B5555 | Nepal 88A BC-7 | 1976 | Hulled grains | 2019 | 43 | 92 |
| B5563 | Nepal 92A BC-4 | 1976 | Hulled grains | 2019 | 43 | 80 |
| B5581 | Nepal 102A BC-4 | 1976 | Hulled grains | 2019 | 43 | 68 |
| B5601 | Nepal 3A BC-4 | 1976 | Hulled grains | 2019 | 43 | 78 |
| B5630 | Nepal 54B BC-2 | 1976 | Hulled grains | 2019 | 43 | 94 |
| B5632 | Nepal 54B BC-4 | 1976 | Hulled grains | 2019 | 43 | 78 |
| B5635 | Nepal 57B BC-2 | 1976 | Hulled grains | 2019 | 43 | 96 |
| B5644 | Nepal 61A BC-3 | 1976 | Hulled grains | 2019 | 43 | 92 |
| B5657 | Nepal 78A BC-3 | 1976 | Hulled grains | 2019 | 43 | 66 |
| B5674 | Nepal 83A BC-7 | 1976 | Hulled grains | 2019 | 43 | 86 |
| B5692 | Nepal 97A BC-3 | 1976 | Hulled grains | 2019 | 43 | 92 |
| B5697 | Nepal 103A BC-3 | 1976 | Hulled grains | 2019 | 43 | 90 |
| B5698 | Nepal 103A BC-4 | 1976 | Hulled grains | 2019 | 43 | 90 |
| B5705 | Nepal 103B BC-7 | 1976 | Hulled grains | 2019 | 43 | 68 |
| B5711 | No 8 | 1976 | Hulled grains | 2019 | 43 | 78 |
| B5723 | CMB-72-123-1Y-1B-1Y-DB | 1976 | Hulled grains | 2019 | 43 | 96 |
| B5750 | BGS 101 | 1976 | Hulled grains | 2019 | 43 | 76 |
| B5852 | Welgevallen 500-1-2-1 | 1976 | Hulled grains | 2019 | 43 | 84 |
| B5864 | Welgevallen 521-3-2 | 1976 | Hulled grains | 2019 | 43 | 64 |
| B5872 | Welgevallen 530-3-3B | 1976 | Hulled grains | 2019 | 43 | 92 |
| B5884 | Welgevallen 582-2-1 | 1976 | Hulled grains | 2019 | 43 | 72 |
| B5887 | Welgevallen 582-3-2B | 1976 | Hulled grains | 2019 | 43 | 50 |
| B5903 | Welgevallen 595-15 | 1976 | Hulled grains | 2019 | 43 | 86 |
| B5917 | Welgevallen 46/76-1-10A | 1976 | Hulled grains | 2019 | 43 | 92 |

| | | | | | | |
|---|---|---|---|---|---|---|
| B5952 | Minnesota M 69-109 | 1976 | Hulled grains | 2019 | 43 | 62 |
| B5955 | M 2657 | 1976 | Hulled grains | 2019 | 43 | 56 |
| B5968 | Nepal 128 | 1976 | Hulled grains | 2019 | 43 | 74 |
| B5969 | Nepal 129 | 1976 | Hulled grains | 2019 | 43 | 50 |
| B5973 | Nepal 135 | 1976 | Hulled grains | 2019 | 43 | 76 |
| B5986 | Nepal 164 | 1976 | Hulled grains | 2019 | 43 | 28 |
| B5992 | Nepal 174 | 1976 | Hulled grains | 2019 | 43 | 18 |
| B8168 | Male Sterile Proctor ms1 | 1976 | Hulled grains | 2019 | 43 | 66 |
| B20120 | Nepal 92A BN-2/1 | 1976 | Hulless | 2019 | 43 | 12 |
| B4723 | Nepal 19A BN-1 | 1976 | Hulless | 2019 | 43 | 12 |
| B4762 | Nepal 72A BN-1 | 1976 | Hulless | 2019 | 43 | 20 |
| B4784 | Nepal 100A BN-1 | 1976 | Hulless | 2019 | 43 | 18 |
| B5546 | Nepal 84A BN-2 | 1976 | Hulless | 2019 | 43 | 16 |
| B5557 | Nepal 88A BC-9 | 1976 | Hulless | 2019 | 43 | 14 |
| B5585 | Nepal 105A BC-2 | 1976 | Hulless | 2019 | 43 | 28 |
| B5656 | Nepal 78A BC-2 | 1976 | Hulless | 2019 | 43 | 44 |
| B5661 | Nepal 78A BC-7 | 1976 | Hulless | 2019 | 43 | 10 |
| B5668 | Nepal 82A BN-4 | 1976 | Hulless | 2019 | 43 | 16 |
| B5069 | HB 791/67/7/5 | 1975 | Hulled grains | 2019 | 44 | 88 |
| B6667 | Nepal 1A BC | 1975 | Hulled grains | 2019 | 44 | 68 |
| B6668 | Nepal 3A BC | 1975 | Hulled grains | 2019 | 44 | 56 |
| B6669 | Nepal 4A BC | 1975 | Hulled grains | 2019 | 44 | 66 |
| B6670 | Nepal 5A BC | 1975 | Hulled grains | 2019 | 44 | 66 |
| B6671 | Nepal 6A BC | 1975 | Hulled grains | 2019 | 44 | 70 |
| B6673 | Nepal 7B BC | 1975 | Hulled grains | 2019 | 44 | 80 |
| B6674 | Nepal 8A BC | 1975 | Hulled grains | 2019 | 44 | 80 |
| B6675 | Nepal 9A BC | 1975 | Hulled grains | 2019 | 44 | 42 |
| B6676 | Nepal 12A BC | 1975 | Hulled grains | 2019 | 44 | 42 |
| B6678 | Nepal 12B BC | 1975 | Hulled grains | 2019 | 44 | 60 |

| | | | | | | |
|---|---|---|---|---|---|---|
| B6679 | Nepal 15A BC | 1975 | Hulled grains | 2019 | 44 | 74 |
| B6681 | Nepal 16A BC | 1975 | Hulled grains | 2019 | 44 | 66 |
| B6682 | Nepal 17A BC | 1975 | Hulled grains | 2019 | 44 | 88 |
| B6684 | Nepal 19B BC | 1975 | Hulled grains | 2019 | 44 | 70 |
| B6685 | Nepal 20A BC | 1975 | Hulled grains | 2019 | 44 | 80 |
| B6686 | Nepal 21A BC | 1975 | Hulled grains | 2019 | 44 | 62 |
| B6688 | Nepal 23A BC | 1975 | Hulled grains | 2019 | 44 | 70 |
| B6689 | Nepal 25A BC | 1975 | Hulled grains | 2019 | 44 | 82 |
| B6692 | Nepal 28A BC | 1975 | Hulled grains | 2019 | 44 | 54 |
| B6696 | Nepal 32A BC | 1975 | Hulled grains | 2019 | 44 | 92 |
| B6697 | Nepal 33A BC | 1975 | Hulled grains | 2019 | 44 | 86 |
| B6698 | Nepal 34A BC | 1975 | Hulled grains | 2019 | 44 | 86 |
| B6699 | Nepal 35A BC | 1975 | Hulled grains | 2019 | 44 | 80 |
| B6700 | Nepal 36A BC | 1975 | Hulled grains | 2019 | 44 | 74 |
| B6703 | Nepal 38A BC | 1975 | Hulled grains | 2019 | 44 | 62 |
| B6704 | Nepal 39A BC | 1975 | Hulled grains | 2019 | 44 | 42 |
| B6705 | Nepal 40A BC | 1975 | Hulled grains | 2019 | 44 | 66 |
| B6706 | Nepal 41A BC | 1975 | Hulled grains | 2019 | 44 | 90 |
| B6708 | Nepal 44A BC | 1975 | Hulled grains | 2019 | 44 | 72 |
| B6709 | Nepal 46A BC | 1975 | Hulled grains | 2019 | 44 | 98 |
| B6710 | Nepal 47A BN | 1975 | Hulled grains | 2019 | 44 | 78 |
| B6711 | Nepal 48A BC | 1975 | Hulled grains | 2019 | 44 | 80 |
| B6712 | Nepal 49A BC | 1975 | Hulled grains | 2019 | 44 | 90 |
| B6713 | Nepal 50A BC | 1975 | Hulled grains | 2019 | 44 | 46 |
| B6714 | Nepal 51A BC | 1975 | Hulled grains | 2019 | 44 | 54 |
| B6715 | Nepal 52A BC | 1975 | Hulled grains | 2019 | 44 | 86 |
| B6716 | Nepal 54A BC | 1975 | Hulled grains | 2019 | 44 | 14 |
| B6718 | Nepal 54B BC | 1975 | Hulled grains | 2019 | 44 | 66 |
| B6720 | Nepal 55A BC | 1975 | Hulled grains | 2019 | 44 | 72 |

| | | | | | | |
|---|---|---|---|---|---|---|
| B6721 | Nepal 56A BC | 1975 | Hulled grains | 2019 | 44 | 62 |
| B6724 | Nepal 57B BC | 1975 | Hulled grains | 2019 | 44 | 62 |
| B6725 | Nepal 58A BC | 1975 | Hulled grains | 2019 | 44 | 68 |
| B6726 | Nepal 59A BC | 1975 | Hulled grains | 2019 | 44 | 42 |
| B6729 | Nepal 61A BC | 1975 | Hulled grains | 2019 | 44 | 50 |
| B6731 | Nepal 61B BC | 1975 | Hulled grains | 2019 | 44 | 42 |
| B6732 | Nepal 62A BC | 1975 | Hulled grains | 2019 | 44 | 26 |
| B6734 | Nepal 65A BC | 1975 | Hulled grains | 2019 | 44 | 64 |
| B6735 | Nepal 65B BC | 1975 | Hulled grains | 2019 | 44 | 66 |
| B6736 | Nepal 65C BC | 1975 | Hulled grains | 2019 | 44 | 64 |
| B6737 | Nepal 66A BC | 1975 | Hulled grains | 2019 | 44 | 44 |
| B6738 | Nepal 67A BN | 1975 | Hulled grains | 2019 | 44 | 30 |
| B6739 | Nepal 68A BC | 1975 | Hulled grains | 2019 | 44 | 66 |
| B6741 | Nepal 72A BC | 1975 | Hulled grains | 2019 | 44 | 76 |
| B6744 | Nepal 73A BC | 1975 | Hulled grains | 2019 | 44 | 74 |
| B6745 | Nepal 75A BC | 1975 | Hulled grains | 2019 | 44 | 36 |
| B6746 | Nepal 77A BC | 1975 | Hulled grains | 2019 | 44 | 62 |
| B6747 | Nepal 78A BC | 1975 | Hulled grains | 2019 | 44 | 12 |
| B6749 | Nepal 79A BC | 1975 | Hulled grains | 2019 | 44 | 50 |
| B6752 | Nepal 83A BC | 1975 | Hulled grains | 2019 | 44 | 36 |
| B6753 | Nepal 83A BN | 1975 | Hulled grains | 2019 | 44 | 30 |
| B6759 | Nepal 88A BC | 1975 | Hulled grains | 2019 | 44 | 24 |
| B6763 | Nepal 92A BC | 1975 | Hulled grains | 2019 | 44 | 34 |
| B6769 | Nepal 97A BC | 1975 | Hulled grains | 2019 | 44 | 50 |
| B6774 | Nepal 101A BN | 1975 | Hulled grains | 2019 | 44 | 44 |
| B6775 | Nepal 102A BC | 1975 | Hulled grains | 2019 | 44 | 64 |
| B6778 | Nepal 103B BC | 1975 | Hulled grains | 2019 | 44 | 56 |
| B6781 | Nepal 106A BC | 1975 | Hulled grains | 2019 | 44 | 78 |
| B6783 | Nepal 107A BC | 1975 | Hulled grains | 2019 | 44 | 48 |

| | | | | | | |
|---|---|---|---|---|---|---|
| B6786 | Nepal 109A BC | 1975 | Hulled grains | 2019 | 44 | 66 |
| B4181 | Tibet 3 | 1975 | Hulless | 2019 | 44 | 0 |
| B4182 | Tibet 4 | 1975 | Hulless | 2019 | 44 | 4 |
| B4198 | Tibet 22 | 1975 | Hulless | 2019 | 44 | 0 |
| B4408 | Ethiopie EP 80 | 1975 | Hulless | 2019 | 44 | 0 |
| B4676 | Mongolei | 1975 | Hulless | 2019 | 44 | 0 |
| B6677 | Nepal 12A BN | 1975 | Hulless | 2019 | 44 | 10 |
| B6680 | Nepal 15A BN | 1975 | Hulless | 2019 | 44 | 10 |
| B6690 | Nepal 25A BN | 1975 | Hulless | 2019 | 44 | 2 |
| B6694 | Nepal 30A BN | 1975 | Hulless | 2019 | 44 | 4 |
| B6702 | Nepal 37A BN | 1975 | Hulless | 2019 | 44 | 0 |
| B6719 | Nepal 54B BN | 1975 | Hulless | 2019 | 44 | 10 |
| B6722 | Nepal 56A BN | 1975 | Hulless | 2019 | 44 | 6 |
| B6728 | Nepal 60A BN | 1975 | Hulless | 2019 | 44 | 6 |
| B6740 | Nepal 68A BN | 1975 | Hulless | 2019 | 44 | 20 |
| B6742 | Nepal 72A BN | 1975 | Hulless | 2019 | 44 | 2 |
| B6743 | Nepal 72B BN | 1975 | Hulless | 2019 | 44 | 8 |
| B6748 | Nepal 78A BN | 1975 | Hulless | 2019 | 44 | 4 |
| B6751 | Nepal 82A BN | 1975 | Hulless | 2019 | 44 | 4 |
| B6754 | Nepal 84A BC | 1975 | Hulless | 2019 | 44 | 10 |
| B6755 | Nepal 84A BN | 1975 | Hulless | 2019 | 44 | 0 |
| B6757 | Nepal 86A BN | 1975 | Hulless | 2019 | 44 | 0 |
| B6758 | Nepal 87A BN | 1975 | Hulless | 2019 | 44 | 2 |
| B6760 | Nepal 88A BN | 1975 | Hulless | 2019 | 44 | 14 |
| B6761 | Nepal 88B BN | 1975 | Hulless | 2019 | 44 | 6 |
| B6762 | Nepal 89A BN | 1975 | Hulless | 2019 | 44 | 4 |
| B6764 | Nepal 92A BN | 1975 | Hulless | 2019 | 44 | 8 |
| B6765 | Nepal 93A BN | 1975 | Hulless | 2019 | 44 | 8 |
| B6771 | Nepal 99A BN | 1975 | Hulless | 2019 | 44 | 8 |

| | | | | | | |
|---|---|---|---|---|---|---|
| B6773 | Nepal 100A BN | 1975 | Hulless | 2019 | 44 | 2 |
| B6782 | Nepal 106A BN | 1975 | Hulless | 2019 | 44 | 2 |
| B3502 | J 53 | 1974 | Hulled grains | 2019 | 45 | 70 |
| B3520 | Monte Cristo | 1974 | Hulled grains | 2019 | 45 | 12 |
| B3580 | South America | 1974 | Hulled grains | 2019 | 45 | 14 |
| B3736 | Spontaneum langunculiforme | 1974 | Hulled grains | 2019 | 45 | 12 |
| B4384 | HB 407/90 | 1974 | Hulled grains | 2019 | 45 | 50 |
| B4465 | Leningrad 13059 | 1974 | Hulled grains | 2019 | 45 | 82 |
| B4466 | Leningrad 13060 | 1974 | Hulled grains | 2019 | 45 | 98 |
| B4503 | H 286 2152 | 1974 | Hulled grains | 2019 | 45 | 52 |
| B4620 | BnP 55 | 1974 | Hulled grains | 2019 | 45 | 56 |
| B4795 | Nepal 109A BC-1 | 1974 | Hulled grains | 2019 | 45 | 98 |
| B5089 | Nepal 22A BC-1 | 1974 | Hulled grains | 2019 | 45 | 60 |
| B5096 | Nepal 40A BC-1 | 1974 | Hulled grains | 2019 | 45 | 90 |
| B5099 | Nepal 54B BC-1 | 1974 | Hulled grains | 2019 | 45 | 84 |
| B5111 | Nepal 78A BC-1 | 1974 | Hulled grains | 2019 | 45 | 70 |
| B3514 | Lyallpur Selection | 1974 | Hulless | 2019 | 45 | 2 |
| B3695 | Shiromugi 8 | 1974 | Hulless | 2019 | 45 | 2 |
| B4190 | Tibet 12 | 1974 | Hulless | 2019 | 45 | 0 |
| B4217 | Tibet 32 | 1974 | Hulless | 2019 | 45 | 0 |
| B4225 | Tibet 13300/16 | 1974 | Hulless | 2019 | 45 | 0 |
| B4349 | Schiemann 1 | 1974 | Hulless | 2019 | 45 | 0 |
| B5102 | Nepal 60A BN-1 | 1974 | Hulless | 2019 | 45 | 12 |
| B5112 | Nepal 82A BN-1 | 1974 | Hulless | 2019 | 45 | 16 |
| B5116 | Nepal 88B BN-1 | 1974 | Hulless | 2019 | 45 | 12 |
| B5117 | Nepal 94A BN-1 | 1974 | Hulless | 2019 | 45 | 12 |

**Supplementary Table S2. All the phenotypes of hulled accessions from seven HYs (1975, 1976, 1979, 2003, 2004, 2016, and 2017) for the macronutrient and grain size data.**

| Accession Code | Harvest Year | Grain type (Hulled/ Hulless) | Germination rate (%) | Grain Protein Content (%) | Grain Starch Content (%) | Grain NDF Content (%) | Thousand Grain Weight (g) | Grain Width (mm) | Grain Length (mm) |
|---|---|---|---|---|---|---|---|---|---|
| B6705 | 1975 | Hulled | 66 | 17.86 | 50.62 | 5.34 | 28.15 | 3.06 | 8.54 |
| B6775 | 1975 | Hulled | 64 | 20.75 | 50.73 | 4.61 | 29.71 | 3.04 | 8.32 |
| B6786 | 1975 | Hulled | 66 | 18.50 | 51.04 | 4.99 | 31.59 | 3.02 | 8.99 |
| B6769 | 1975 | Hulled | 50 | 20.00 | 51.15 | 4.69 | 31.60 | 3.13 | 8.57 |
| B6724 | 1975 | Hulled | 62 | 19.83 | 51.15 | 4.91 | 31.62 | 3.18 | 8.92 |
| B6737 | 1975 | Hulled | 44 | 19.36 | 51.24 | 4.97 | 32.44 | 3.19 | 8.40 |
| B6739 | 1975 | Hulled | 66 | 21.49 | 51.36 | 3.98 | 38.28 | 3.20 | 9.04 |
| B6708 | 1975 | Hulled | 72 | 19.62 | 51.67 | 4.59 | 33.92 | 3.14 | 8.85 |
| B6709 | 1975 | Hulled | 98 | 18.35 | 51.82 | 5.01 | 28.93 | 3.14 | 8.31 |
| B6718 | 1975 | Hulled | 66 | 19.07 | 51.92 | 4.97 | 30.42 | 3.21 | 8.15 |
| B6703 | 1975 | Hulled | 62 | 18.03 | 51.92 | 4.77 | 31.79 | 3.05 | 8.93 |
| B6689 | 1975 | Hulled | 82 | 19.92 | 51.97 | 4.46 | 30.93 | 3.21 | 7.99 |
| B6686 | 1975 | Hulled | 62 | 19.24 | 52.10 | 4.81 | 31.17 | 3.19 | 8.30 |
| B6753 | 1975 | Hulled | 30 | 21.22 | 52.10 | 3.99 | 31.74 | 3.17 | 8.43 |
| B6778 | 1975 | Hulled | 56 | 18.80 | 52.13 | 4.88 | 30.19 | 3.06 | 8.65 |
| B6678 | 1975 | Hulled | 60 | 19.57 | 52.28 | 4.87 | 31.03 | 3.08 | 9.00 |
| B6699 | 1975 | Hulled | 80 | 17.91 | 52.28 | 4.66 | 34.18 | 3.15 | 9.11 |
| B6681 | 1975 | Hulled | 66 | 19.09 | 52.32 | 4.54 | 30.53 | 3.22 | 7.55 |
| B6726 | 1975 | Hulled | 42 | 19.16 | 52.36 | 4.71 | 28.25 | 2.95 | 8.55 |
| B6711 | 1975 | Hulled | 80 | 20.02 | 52.45 | 4.59 | 27.46 | 3.12 | 7.59 |
| B6781 | 1975 | Hulled | 78 | 19.10 | 52.47 | 4.72 | 28.15 | 2.99 | 8.55 |

| | | | | | | | | | |
|---|---|---|---|---|---|---|---|---|---|
| B6774 | 1975 | Hulled | 44 | 20.72 | 52.47 | 4.00 | 29.78 | 3.02 | 8.33 |
| B6736 | 1975 | Hulled | 64 | 19.18 | 52.47 | 5.05 | 33.11 | 3.20 | 8.59 |
| B6729 | 1975 | Hulled | 50 | 20.12 | 52.49 | 4.56 | 34.52 | 3.18 | 8.86 |
| B6704 | 1975 | Hulled | 42 | 17.95 | 52.51 | 4.80 | 35.77 | 3.20 | 8.59 |
| B6679 | 1975 | Hulled | 74 | 19.09 | 52.59 | 4.36 | 28.62 | 3.11 | 7.93 |
| B6721 | 1975 | Hulled | 62 | 20.04 | 52.75 | 4.47 | 29.64 | 3.12 | 8.28 |
| B6720 | 1975 | Hulled | 72 | 17.15 | 52.79 | 4.86 | 32.98 | 3.26 | 8.33 |
| B6698 | 1975 | Hulled | 86 | 17.36 | 52.91 | 4.99 | 27.93 | 2.90 | 9.18 |
| B6714 | 1975 | Hulled | 54 | 17.01 | 53.11 | 5.29 | 29.01 | 3.03 | 8.68 |
| B6712 | 1975 | Hulled | 90 | 16.25 | 53.13 | 5.31 | 31.51 | 3.06 | 8.75 |
| B6735 | 1975 | Hulled | 66 | 18.16 | 53.15 | 4.13 | 37.43 | 3.35 | 8.38 |
| B6710 | 1975 | Hulled | 78 | 20.08 | 53.17 | 4.06 | 31.39 | 3.12 | 8.40 |
| B6696 | 1975 | Hulled | 92 | 16.32 | 53.24 | 4.81 | 29.64 | 2.99 | 9.88 |
| B6746 | 1975 | Hulled | 62 | 22.19 | 53.31 | 3.79 | 34.75 | 3.21 | 7.88 |
| B6688 | 1975 | Hulled | 70 | 16.86 | 53.36 | 4.76 | 24.22 | 2.83 | 8.36 |
| B6752 | 1975 | Hulled | 36 | 20.42 | 53.40 | 4.61 | 29.53 | 3.04 | 8.06 |
| B6725 | 1975 | Hulled | 68 | 17.18 | 53.42 | 5.18 | 34.05 | 3.18 | 9.06 |
| B6759 | 1975 | Hulled | 24 | 22.19 | 53.63 | 3.68 | 26.44 | 3.04 | 7.98 |
| B6734 | 1975 | Hulled | 64 | 19.13 | 53.74 | 3.85 | 36.59 | 3.28 | 8.22 |
| B6744 | 1975 | Hulled | 74 | 17.96 | 53.79 | 4.36 | 32.80 | 3.14 | 8.46 |
| B6667 | 1975 | Hulled | 68 | 16.79 | 53.81 | 5.01 | 26.66 | 2.85 | 8.53 |
| B6749 | 1975 | Hulled | 50 | 19.69 | 54.03 | 3.98 | 33.93 | 3.19 | 8.26 |
| B6685 | 1975 | Hulled | 80 | 16.29 | 54.15 | 4.77 | 32.08 | 3.13 | 9.06 |
| B6741 | 1975 | Hulled | 76 | 18.05 | 54.15 | 3.73 | 33.92 | 3.26 | 7.83 |
| B6700 | 1975 | Hulled | 74 | 17.57 | 54.16 | 4.26 | 33.85 | 3.09 | 9.44 |
| B6692 | 1975 | Hulled | 54 | 16.44 | 54.32 | 4.47 | 32.07 | 3.13 | 9.01 |
| B6713 | 1975 | Hulled | 46 | 21.47 | 54.63 | 3.68 | 33.55 | 3.15 | 7.73 |

| | | | | | | | | | |
|---|---|---|---|---|---|---|---|---|---|
| B6715 | 1975 | Hulled | 86 | 15.86 | 54.65 | 4.76 | 29.70 | 3.06 | 8.73 |
| B6697 | 1975 | Hulled | 86 | 16.42 | 54.71 | 4.60 | 26.83 | 2.90 | 9.50 |
| B6731 | 1975 | Hulled | 42 | 20.71 | 54.71 | 3.56 | 30.71 | 3.15 | 7.69 |
| B6706 | 1975 | Hulled | 90 | 18.80 | 54.73 | 4.10 | 31.18 | 3.25 | 7.34 |
| B6783 | 1975 | Hulled | 48 | 19.45 | 55.19 | 4.28 | 35.20 | 3.20 | 9.05 |
| B6684 | 1975 | Hulled | 70 | 18.02 | 55.52 | 4.17 | 27.71 | 3.02 | 7.84 |
| B6732 | 1975 | Hulled | 26 | 21.18 | 55.53 | 3.27 | 30.89 | 3.10 | 7.58 |
| B6763 | 1975 | Hulled | 34 | 21.89 | 55.69 | 3.19 | 32.59 | 3.17 | 7.64 |
| B6682 | 1975 | Hulled | 88 | 17.00 | 56.31 | 4.07 | 30.38 | 3.10 | 7.78 |
| B5069 | 1975 | Hulled | 88 | 17.48 | 56.89 | 4.66 | 33.01 | 3.20 | 8.12 |
| B6745 | 1975 | Hulled | 36 | 21.38 | 57.67 | 2.96 | 30.71 | 3.09 | 7.75 |
| B6738 | 1975 | Hulled | 30 | 20.70 | 57.97 | 3.38 | 31.91 | 3.07 | 7.75 |
| B6747 | 1975 | Hulled | 12 | 22.19 | 59.13 | 2.76 | 25.59 | 3.02 | 7.22 |
| B6716 | 1975 | Hulled | 14 | 21.49 | 59.26 | 2.55 | 28.34 | 3.07 | 6.94 |
| B5905 | 1976 | Hulled | 72 | 18.55 | 58.90 | 4.57 | 32.72 | 3.16 | 10.14 |
| B5906 | 1976 | Hulled | 68 | 19.17 | 59.30 | 4.69 | 32.37 | 3.07 | 10.00 |
| B5908 | 1976 | Hulled | 46 | 18.62 | 58.68 | 4.77 | 34.63 | 3.17 | 10.03 |
| B5912 | 1976 | Hulled | 46 | 20.05 | 59.24 | 4.47 | 46.62 | 3.50 | 9.19 |
| B5915 | 1976 | Hulled | 46 | 18.68 | 58.63 | 5.22 | 43.40 | 3.22 | 11.79 |
| B5916 | 1976 | Hulled | 58 | 19.12 | 58.05 | 5.42 | 42.83 | 3.22 | 12.24 |
| B5917 | 1976 | Hulled | 60 | 18.91 | 57.24 | 5.99 | 42.53 | 3.29 | 12.29 |
| B5921 | 1976 | Hulled | 62 | 17.98 | 57.27 | 5.67 | 45.88 | 3.36 | 12.32 |
| B5923 | 1976 | Hulled | 58 | 19.60 | 59.18 | 4.61 | 37.44 | 3.20 | 9.72 |
| B5924 | 1976 | Hulled | 20 | 20.45 | 59.34 | 4.47 | 41.30 | 3.26 | 10.50 |
| B5925 | 1976 | Hulled | 68 | 18.32 | 59.80 | 4.68 | 39.32 | 3.41 | 10.06 |
| B5926 | 1976 | Hulled | 66 | 20.39 | 57.34 | 5.14 | 34.87 | 3.02 | 11.01 |
| B5938 | 1976 | Hulled | 62 | 16.77 | 57.32 | 5.55 | 40.81 | 3.39 | 10.36 |

| | | | | | | | | | |
|---|---|---|---|---|---|---|---|---|---|
| B5941 | 1976 | Hulled | 92 | 21.35 | 57.74 | 4.69 | 43.63 | 3.66 | 8.35 |
| B5947 | 1976 | Hulled | 58 | 10.08 | 64.16 | 3.00 | 41.07 | 3.46 | 7.99 |
| B5948 | 1976 | Hulled | 22 | 15.15 | 61.79 | 3.31 | 37.86 | 3.31 | 8.07 |
| B5951 | 1976 | Hulled | 74 | 17.23 | 58.17 | 5.02 | 28.91 | 3.24 | 8.54 |
| B5952 | 1976 | Hulled | 42 | 17.95 | 57.05 | 5.93 | 31.96 | 3.25 | 9.91 |
| B5955 | 1976 | Hulled | 72 | 16.79 | 58.20 | 5.10 | 26.60 | 3.24 | 9.00 |
| B5965 | 1976 | Hulled | 24 | 13.62 | 59.15 | 4.42 | 25.67 | 2.94 | 8.34 |
| B5967 | 1976 | Hulled | 38 | 12.42 | 58.75 | 5.77 | 20.00 | 2.85 | 9.15 |
| B5968 | 1976 | Hulled | 84 | 12.02 | 60.56 | 4.71 | 29.55 | 2.97 | 8.80 |
| B5969 | 1976 | Hulled | 62 | 12.70 | 59.84 | 5.31 | 24.35 | 2.82 | 8.58 |
| B5970 | 1976 | Hulled | 24 | 14.17 | 58.99 | 6.11 | 18.54 | 2.72 | 9.26 |
| B5971 | 1976 | Hulled | 94 | 14.02 | 57.67 | 4.95 | 31.38 | 3.07 | 9.28 |
| B5972 | 1976 | Hulled | 84 | 10.99 | 60.40 | 4.59 | 28.93 | 3.11 | 8.97 |
| B5973 | 1976 | Hulled | 82 | 11.15 | 60.52 | 4.65 | 29.57 | 3.18 | 8.59 |
| B5974 | 1976 | Hulled | 2 | 15.15 | 55.15 | 7.59 | 15.35 | 2.95 | 8.64 |
| B5975 | 1976 | Hulled | 48 | 14.40 | 56.09 | 6.20 | 23.50 | 3.04 | 8.65 |
| B5976 | 1976 | Hulled | 78 | 11.44 | 58.63 | 5.09 | 26.51 | 2.93 | 8.02 |
| B5977 | 1976 | Hulled | 84 | 11.87 | 58.86 | 4.88 | 27.39 | 2.99 | 8.52 |
| B5978 | 1976 | Hulled | 64 | 13.13 | 60.44 | 5.73 | 20.26 | 2.92 | 8.30 |
| B5979 | 1976 | Hulled | 22 | 14.05 | 58.30 | 4.93 | 23.09 | 2.91 | 8.23 |
| B5980 | 1976 | Hulled | 40 | 11.14 | 68.87 | 1.52 | 29.45 | 3.73 | 6.65 |
| B5984 | 1976 | Hulled | 8 | 14.80 | 64.46 | 2.19 | 24.95 | 3.68 | 6.42 |
| B5985 | 1976 | Hulled | 2 | 19.42 | 55.73 | 5.55 | 24.25 | 2.99 | 8.51 |
| B5986 | 1976 | Hulled | 26 | 14.69 | 54.60 | 5.26 | 29.69 | 3.08 | 8.62 |
| B5987 | 1976 | Hulled | 0 | 16.10 | 65.26 | 2.04 | 22.55 | 3.55 | 7.06 |
| B5988 | 1976 | Hulled | 56 | 13.29 | 59.31 | 5.03 | 26.78 | 3.05 | 8.69 |
| B5989 | 1976 | Hulled | 46 | 11.88 | 57.34 | 5.01 | 29.10 | 3.06 | 8.89 |

| | | | | | | | | | |
|---|---|---|---|---|---|---|---|---|---|
| B5990 | 1976 | Hulled | 32 | 11.19 | 65.46 | 2.08 | 28.53 | 3.82 | 6.45 |
| B5991 | 1976 | Hulled | 4 | 15.84 | 55.63 | 5.18 | 23.40 | 2.95 | 8.48 |
| B5992 | 1976 | Hulled | 4 | 15.63 | 56.70 | 5.19 | 20.59 | 2.69 | 8.04 |
| B5994 | 1976 | Hulled | 72 | 14.63 | 58.13 | 4.50 | 29.57 | 3.13 | 8.41 |
| B6025 | 1976 | Hulled | 82 | 16.75 | 60.10 | 3.76 | 41.61 | 3.67 | 7.88 |
| B6037 | 1976 | Hulled | 92 | 15.17 | 59.68 | 3.53 | 38.43 | 3.36 | 8.70 |
| B6069 | 1976 | Hulled | 80 | 16.43 | 59.06 | 3.93 | 48.81 | 3.79 | 9.45 |
| B6138 | 1976 | Hulled | 70 | 12.18 | 61.74 | 3.46 | 38.05 | 3.45 | 8.14 |
| B6172 | 1976 | Hulled | 98 | 15.74 | 59.32 | 3.94 | 38.03 | 3.31 | 9.86 |
| B6188 | 1976 | Hulled | 76 | 18.33 | 58.21 | 4.32 | 32.36 | 3.32 | 8.51 |
| B5488 | 1976 | Hulled | 66 | 17.70 | 59.89 | 4.24 | 35.22 | 3.07 | 9.12 |
| B5489 | 1976 | Hulled | 28 | 16.57 | 60.31 | 3.96 | 37.43 | 3.13 | 9.72 |
| B5490 | 1976 | Hulled | 46 | 16.54 | 59.78 | 4.15 | 34.32 | 3.11 | 8.63 |
| B5491 | 1976 | Hulled | 52 | 16.00 | 58.14 | 4.28 | 43.12 | 3.39 | 8.66 |
| B5492 | 1976 | Hulled | 66 | 16.47 | 58.56 | 4.16 | 38.53 | 3.24 | 9.35 |
| B5493 | 1976 | Hulled | 60 | 15.80 | 59.40 | 3.96 | 37.30 | 3.22 | 8.71 |
| B5494 | 1976 | Hulled | 42 | 16.51 | 59.23 | 3.90 | 42.89 | 3.32 | 9.14 |
| B5495 | 1976 | Hulled | 36 | 17.62 | 58.69 | 3.95 | 43.81 | 3.34 | 8.94 |
| B5496 | 1976 | Hulled | 14 | 15.00 | 68.37 | 1.94 | 26.33 | 2.93 | 6.26 |
| B5497 | 1976 | Hulled | 18 | 15.68 | 67.34 | 1.71 | 33.69 | 3.29 | 6.99 |
| B5498 | 1976 | Hulled | 72 | 16.72 | 59.37 | 4.32 | 31.95 | 3.05 | 8.70 |
| B5499 | 1976 | Hulled | 74 | 16.91 | 59.54 | 3.75 | 37.65 | 3.17 | 8.91 |
| B5500 | 1976 | Hulled | 70 | 19.83 | 61.40 | 3.13 | 30.45 | 3.07 | 8.08 |
| B5501 | 1976 | Hulled | 92 | 16.58 | 59.37 | 4.33 | 33.88 | 3.12 | 9.07 |
| B5502 | 1976 | Hulled | 96 | 17.61 | 58.13 | 5.06 | 25.50 | 2.77 | 9.95 |
| B5503 | 1976 | Hulled | 96 | 16.28 | 58.14 | 5.13 | 26.84 | 2.83 | 9.88 |
| B5504 | 1976 | Hulled | 92 | 18.77 | 58.53 | 4.39 | 21.29 | 2.65 | 8.61 |

| | | | | | | | | | |
|---|---|---|---|---|---|---|---|---|---|
| B5505 | 1976 | Hulled | 76 | 15.67 | 58.93 | 4.62 | 29.72 | 2.85 | 9.42 |
| B5506 | 1976 | Hulled | 88 | 16.26 | 58.92 | 4.76 | 28.19 | 2.79 | 9.00 |
| B5507 | 1976 | Hulled | 98 | 16.45 | 58.69 | 4.61 | 28.93 | 2.82 | 9.21 |
| B5509 | 1976 | Hulled | 94 | 15.73 | 58.73 | 4.53 | 34.75 | 3.09 | 9.18 |
| B5510 | 1976 | Hulled | 84 | 15.42 | 59.36 | 4.51 | 33.54 | 2.97 | 9.25 |
| B5511 | 1976 | Hulled | 86 | 16.00 | 59.09 | 4.44 | 32.81 | 3.02 | 8.79 |
| B5512 | 1976 | Hulled | 92 | 16.33 | 58.75 | 4.72 | 30.47 | 2.91 | 9.35 |
| B5513 | 1976 | Hulled | 64 | 16.11 | 60.45 | 4.00 | 33.35 | 3.09 | 8.56 |
| B5514 | 1976 | Hulled | 88 | 14.38 | 59.06 | 4.51 | 38.03 | 3.17 | 9.51 |
| B5515 | 1976 | Hulled | 80 | 15.31 | 59.53 | 4.05 | 38.99 | 3.16 | 9.16 |
| B5517 | 1976 | Hulled | 64 | 16.89 | 59.10 | 4.25 | 44.20 | 3.32 | 9.54 |
| B5518 | 1976 | Hulled | 80 | 15.67 | 58.57 | 4.53 | 41.45 | 3.25 | 9.92 |
| B5519 | 1976 | Hulled | 90 | 16.02 | 58.21 | 4.57 | 42.77 | 3.22 | 10.09 |
| B5520 | 1976 | Hulled | 74 | 15.21 | 58.96 | 4.11 | 32.48 | 3.04 | 9.36 |
| B5521 | 1976 | Hulled | 82 | 15.78 | 58.52 | 4.47 | 32.16 | 3.03 | 8.59 |
| B5522 | 1976 | Hulled | 20 | 13.88 | 68.08 | 2.04 | 29.66 | 3.13 | 6.47 |
| B5523 | 1976 | Hulled | 20 | 14.22 | 67.63 | 2.05 | 32.92 | 3.23 | 6.50 |
| B5524 | 1976 | Hulled | 82 | 14.18 | 60.03 | 3.72 | 35.02 | 3.18 | 8.18 |
| B5526 | 1976 | Hulled | 74 | 15.09 | 60.14 | 4.04 | 33.63 | 3.08 | 8.80 |
| B5528 | 1976 | Hulled | 30 | 14.53 | 67.13 | 1.77 | 37.71 | 3.36 | 6.53 |
| B5531 | 1976 | Hulled | 78 | 18.15 | 58.78 | 4.26 | 31.92 | 3.09 | 8.86 |
| B5532 | 1976 | Hulled | 64 | 16.13 | 60.12 | 4.03 | 33.48 | 3.09 | 8.40 |
| B5536 | 1976 | Hulled | 88 | 14.29 | 60.72 | 3.82 | 32.35 | 3.07 | 8.41 |
| B5539 | 1976 | Hulled | 32 | 16.35 | 59.48 | 4.01 | 40.87 | 3.19 | 9.26 |
| B5541 | 1976 | Hulled | 66 | 16.32 | 58.93 | 4.14 | 41.13 | 3.14 | 9.69 |
| B5542 | 1976 | Hulled | 30 | 14.90 | 68.28 | 1.91 | 26.96 | 2.99 | 6.62 |
| B5546 | 1976 | Hulled | 18 | 17.69 | 67.27 | 2.18 | 24.74 | 2.87 | 6.49 |

| | | | | | | | | | |
|---|---|---|---|---|---|---|---|---|---|
| B5547 | 1976 | Hulled | 20 | 17.50 | 67.45 | 2.28 | 24.88 | 2.99 | 6.32 |
| B5548 | 1976 | Hulled | 8 | 18.83 | 66.99 | 2.17 | 26.48 | 2.90 | 6.53 |
| B5552 | 1976 | Hulled | 62 | 17.44 | 58.57 | 4.22 | 31.72 | 2.96 | 8.53 |
| B5553 | 1976 | Hulled | 20 | 18.42 | 66.60 | 2.09 | 27.39 | 3.57 | 6.91 |
| B5554 | 1976 | Hulled | 72 | 15.81 | 58.84 | 4.18 | 31.84 | 3.05 | 8.50 |
| B5555 | 1976 | Hulled | 74 | 16.66 | 58.28 | 4.11 | 38.59 | 3.26 | 8.38 |
| B5558 | 1976 | Hulled | 28 | 17.77 | 58.37 | 4.27 | 29.25 | 2.84 | 8.81 |
| B5559 | 1976 | Hulled | 78 | 16.04 | 58.43 | 4.03 | 36.09 | 3.17 | 8.54 |
| B5563 | 1976 | Hulled | 86 | 16.69 | 59.14 | 4.03 | 31.95 | 3.02 | 9.01 |
| B5564 | 1976 | Hulled | 72 | 16.16 | 58.70 | 4.06 | 41.91 | 3.30 | 9.22 |
| B5565 | 1976 | Hulled | 46 | 17.30 | 61.04 | 3.13 | 33.66 | 3.17 | 7.92 |
| B5566 | 1976 | Hulled | 20 | 18.74 | 66.21 | 2.16 | 30.28 | 3.65 | 7.04 |
| B5569 | 1976 | Hulled | 72 | 17.67 | 58.73 | 3.97 | 37.77 | 3.20 | 9.31 |
| B5572 | 1976 | Hulled | 58 | 15.84 | 59.42 | 3.85 | 37.53 | 3.27 | 8.90 |
| B5574 | 1976 | Hulled | 22 | 18.55 | 65.90 | 2.13 | 28.25 | 3.46 | 7.29 |
| B5575 | 1976 | Hulled | 30 | 20.45 | 65.72 | 1.96 | 32.29 | 3.65 | 7.45 |
| B5576 | 1976 | Hulled | 78 | 14.85 | 59.46 | 3.92 | 34.71 | 3.08 | 9.01 |
| B5577 | 1976 | Hulled | 84 | 15.41 | 59.46 | 4.05 | 34.60 | 3.05 | 9.03 |
| B5579 | 1976 | Hulled | 60 | 16.26 | 58.69 | 3.84 | 38.39 | 3.20 | 8.96 |
| B5581 | 1976 | Hulled | 60 | 16.49 | 59.30 | 3.84 | 36.20 | 3.18 | 9.46 |
| B5583 | 1976 | Hulled | 6 | 20.76 | 64.75 | 1.90 | 34.34 | 3.73 | 7.43 |
| B5584 | 1976 | Hulled | 70 | 16.74 | 58.26 | 4.38 | 36.53 | 3.54 | 9.50 |
| B5585 | 1976 | Hulled | 18 | 22.69 | 63.96 | 1.96 | 33.81 | 3.72 | 7.56 |
| B5586 | 1976 | Hulled | 42 | 17.08 | 58.92 | 3.85 | 41.66 | 3.31 | 8.49 |
| B5587 | 1976 | Hulled | 62 | 16.56 | 59.23 | 4.12 | 31.73 | 3.06 | 8.83 |
| B5590 | 1976 | Hulled | 90 | 16.52 | 58.77 | 4.49 | 28.09 | 2.88 | 9.30 |
| B5591 | 1976 | Hulled | 72 | 17.89 | 59.35 | 4.03 | 30.55 | 2.91 | 9.20 |

| | | | | | | | | | |
|---|---|---|---|---|---|---|---|---|---|
| B5592 | 1976 | Hulled | 16 | 20.06 | 64.64 | 2.06 | 31.18 | 3.85 | 6.97 |
| B5593 | 1976 | Hulled | 18 | 20.33 | 63.91 | 2.21 | 34.15 | 3.90 | 7.24 |
| B5594 | 1976 | Hulled | 36 | 20.94 | 64.64 | 2.04 | 27.77 | 3.63 | 7.00 |
| B5595 | 1976 | Hulled | 40 | 19.01 | 65.93 | 2.09 | 28.93 | 3.61 | 6.96 |
| B5601 | 1976 | Hulled | 82 | 16.86 | 58.86 | 4.35 | 35.26 | 3.06 | 8.78 |
| B5602 | 1976 | Hulled | 70 | 17.06 | 59.05 | 4.49 | 31.72 | 2.95 | 9.93 |
| B5604 | 1976 | Hulled | 70 | 17.65 | 58.81 | 4.20 | 33.32 | 2.94 | 9.07 |
| B5607 | 1976 | Hulled | 82 | 16.69 | 59.23 | 4.41 | 25.94 | 2.68 | 9.43 |
| B5608 | 1976 | Hulled | 86 | 15.95 | 58.64 | 4.50 | 31.87 | 2.97 | 9.60 |
| B5609 | 1976 | Hulled | 60 | 16.66 | 59.07 | 4.18 | 36.56 | 3.15 | 8.90 |
| B5611 | 1976 | Hulled | 74 | 17.42 | 58.78 | 4.06 | 38.47 | 3.21 | 9.01 |
| B5615 | 1976 | Hulled | 96 | 15.64 | 60.00 | 3.94 | 29.49 | 2.98 | 9.08 |
| B5616 | 1976 | Hulled | 54 | 18.26 | 58.60 | 3.87 | 41.54 | 3.16 | 10.33 |
| B5617 | 1976 | Hulled | 82 | 15.52 | 58.77 | 4.51 | 33.20 | 3.03 | 9.24 |
| B5618 | 1976 | Hulled | 76 | 15.64 | 58.75 | 4.47 | 35.46 | 3.20 | 8.92 |
| B5620 | 1976 | Hulled | 38 | 17.24 | 58.14 | 4.03 | 42.92 | 3.23 | 9.99 |
| B5621 | 1976 | Hulled | 94 | 17.00 | 59.43 | 4.04 | 30.89 | 3.14 | 8.31 |
| B5622 | 1976 | Hulled | 64 | 16.35 | 59.40 | 4.25 | 29.89 | 2.90 | 9.16 |
| B5623 | 1976 | Hulled | 88 | 17.30 | 58.40 | 4.62 | 31.34 | 2.94 | 9.91 |
| B5624 | 1976 | Hulled | 90 | 15.58 | 59.00 | 4.59 | 25.25 | 2.64 | 10.25 |
| B5625 | 1976 | Hulled | 78 | 15.67 | 59.46 | 4.14 | 32.18 | 2.94 | 9.42 |
| B5627 | 1976 | Hulled | 78 | 14.64 | 59.36 | 3.96 | 36.16 | 3.16 | 8.86 |
| B5628 | 1976 | Hulled | 90 | 14.95 | 59.42 | 3.96 | 30.66 | 2.96 | 8.97 |
| B5629 | 1976 | Hulled | 76 | 16.39 | 59.04 | 4.07 | 42.73 | 3.32 | 9.08 |
| B5630 | 1976 | Hulled | 92 | 16.02 | 58.16 | 4.01 | 40.89 | 3.32 | 8.61 |
| B5631 | 1976 | Hulled | 78 | 17.12 | 59.43 | 4.30 | 31.18 | 3.07 | 8.43 |
| B5632 | 1976 | Hulled | 70 | 15.19 | 57.97 | 4.17 | 44.63 | 3.35 | 9.82 |

| | | | | | | | | | |
|---|---|---|---|---|---|---|---|---|---|
| B5633 | 1976 | Hulled | 78 | 16.33 | 59.04 | 4.33 | 30.93 | 3.13 | 8.55 |
| B5634 | 1976 | Hulled | 12 | 15.84 | 67.43 | 1.89 | 24.05 | 3.35 | 6.64 |
| B5635 | 1976 | Hulled | 98 | 16.83 | 58.76 | 4.04 | 38.89 | 3.13 | 9.27 |
| B5636 | 1976 | Hulled | 84 | 16.09 | 59.33 | 4.07 | 40.67 | 3.26 | 9.39 |
| B5637 | 1976 | Hulled | 84 | 15.95 | 59.08 | 3.96 | 37.24 | 3.18 | 9.25 |
| B5638 | 1976 | Hulled | 62 | 16.46 | 58.85 | 3.82 | 41.85 | 3.29 | 9.23 |
| B5639 | 1976 | Hulled | 22 | 16.13 | 66.04 | 1.97 | 27.97 | 3.53 | 6.80 |
| B5640 | 1976 | Hulled | 74 | 17.56 | 57.94 | 4.39 | 37.75 | 3.13 | 9.79 |
| B5642 | 1976 | Hulled | 82 | 16.00 | 59.18 | 3.98 | 40.45 | 3.21 | 9.31 |
| B5643 | 1976 | Hulled | 74 | 17.04 | 60.02 | 3.45 | 32.91 | 3.05 | 8.72 |
| B5644 | 1976 | Hulled | 70 | 17.62 | 58.69 | 4.05 | 34.11 | 3.16 | 8.10 |
| B5645 | 1976 | Hulled | 54 | 16.34 | 64.00 | 2.48 | 28.00 | 2.94 | 7.05 |
| B5655 | 1976 | Hulled | 28 | 21.36 | 64.92 | 1.87 | 25.08 | 3.39 | 6.98 |
| B5656 | 1976 | Hulled | 28 | 17.63 | 66.22 | 1.34 | 29.48 | 3.56 | 6.96 |
| B5657 | 1976 | Hulled | 76 | 15.91 | 59.01 | 3.87 | 37.83 | 3.19 | 8.73 |
| B5658 | 1976 | Hulled | 88 | 16.40 | 59.50 | 3.55 | 38.87 | 3.36 | 8.31 |
| B5659 | 1976 | Hulled | 78 | 15.88 | 57.48 | 4.41 | 36.44 | 3.19 | 8.69 |
| B5660 | 1976 | Hulled | 64 | 17.53 | 59.40 | 3.67 | 30.00 | 3.10 | 8.12 |
| B5661 | 1976 | Hulled | 8 | 15.66 | 68.28 | 1.70 | 30.31 | 3.54 | 7.14 |
| B5662 | 1976 | Hulled | 86 | 17.46 | 58.01 | 4.49 | 30.94 | 3.00 | 8.82 |
| B5663 | 1976 | Hulled | 76 | 17.21 | 59.05 | 4.10 | 26.64 | 2.89 | 8.29 |
| B5664 | 1976 | Hulled | 22 | 15.30 | 69.62 | 1.50 | 30.25 | 3.59 | 7.04 |
| B5665 | 1976 | Hulled | 26 | 16.32 | 67.79 | 1.88 | 24.85 | 3.36 | 7.05 |
| B5666 | 1976 | Hulled | 18 | 16.15 | 66.41 | 1.96 | 28.99 | 3.57 | 7.09 |
| B5667 | 1976 | Hulled | 12 | 15.32 | 67.52 | 1.87 | 30.23 | 3.64 | 6.89 |
| B5668 | 1976 | Hulled | 18 | 15.67 | 68.23 | 1.89 | 27.79 | 3.51 | 6.92 |
| B5669 | 1976 | Hulled | 92 | 17.20 | 59.05 | 3.96 | 32.00 | 3.01 | 9.31 |

| | | | | | | | | | |
|---|---|---|---|---|---|---|---|---|---|
| B5670 | 1976 | Hulled | 94 | 16.85 | 59.49 | 3.77 | 31.51 | 2.94 | 8.56 |
| B5671 | 1976 | Hulled | 94 | 18.55 | 58.47 | 4.40 | 24.70 | 2.80 | 8.43 |
| B5672 | 1976 | Hulled | 84 | 16.91 | 59.09 | 3.97 | 31.98 | 2.94 | 8.44 |
| B5673 | 1976 | Hulled | 32 | 18.43 | 59.41 | 3.41 | 36.42 | 3.03 | 8.87 |
| B5674 | 1976 | Hulled | 84 | 17.60 | 58.19 | 4.08 | 33.88 | 3.02 | 8.90 |
| B5676 | 1976 | Hulled | 96 | 16.60 | 59.68 | 3.72 | 30.58 | 2.93 | 8.70 |
| B5677 | 1976 | Hulled | 28 | 19.46 | 67.05 | 1.91 | 24.58 | 3.41 | 7.01 |
| B5678 | 1976 | Hulled | 74 | 15.59 | 60.01 | 3.59 | 28.01 | 2.88 | 8.33 |
| B5679 | 1976 | Hulled | 90 | 15.49 | 59.60 | 3.66 | 29.40 | 2.92 | 8.50 |
| B5680 | 1976 | Hulled | 82 | 17.41 | 58.25 | 4.28 | 29.46 | 2.83 | 9.23 |
| B5681 | 1976 | Hulled | 30 | 17.45 | 62.66 | 2.43 | 33.25 | 3.21 | 7.48 |
| B5682 | 1976 | Hulled | 34 | 17.82 | 58.33 | 4.34 | 30.31 | 2.86 | 9.05 |
| B5683 | 1976 | Hulled | 26 | 20.18 | 67.21 | 1.78 | 26.50 | 3.43 | 7.06 |
| B5684 | 1976 | Hulled | 68 | 16.58 | 59.38 | 3.75 | 36.67 | 3.25 | 8.17 |
| B5685 | 1976 | Hulled | 20 | 19.31 | 64.36 | 2.19 | 27.90 | 3.01 | 6.62 |
| B5686 | 1976 | Hulled | 76 | 15.50 | 58.44 | 3.82 | 39.06 | 3.13 | 9.40 |
| B5687 | 1976 | Hulled | 86 | 15.70 | 59.33 | 3.82 | 33.98 | 3.15 | 8.42 |
| B5688 | 1976 | Hulled | 86 | 16.79 | 59.26 | 3.98 | 34.64 | 3.19 | 8.45 |
| B5689 | 1976 | Hulled | 16 | 15.10 | 66.93 | 1.75 | 31.57 | 3.62 | 7.14 |
| B5690 | 1976 | Hulled | 18 | 15.33 | 66.77 | 1.71 | 31.61 | 3.62 | 7.12 |
| B5691 | 1976 | Hulled | 88 | 15.18 | 58.64 | 4.28 | 35.38 | 3.14 | 8.79 |
| B5692 | 1976 | Hulled | 88 | 15.04 | 58.91 | 4.32 | 33.09 | 3.05 | 8.70 |
| B5693 | 1976 | Hulled | 88 | 16.83 | 59.08 | 3.88 | 30.66 | 2.94 | 8.82 |
| B5696 | 1976 | Hulled | 90 | 16.27 | 58.78 | 4.38 | 28.25 | 2.88 | 9.85 |
| B5697 | 1976 | Hulled | 78 | 16.12 | 59.95 | 3.65 | 32.05 | 2.98 | 9.19 |
| B5698 | 1976 | Hulled | 68 | 16.74 | 58.26 | 4.24 | 40.27 | 3.24 | 9.49 |
| B5700 | 1976 | Hulled | 34 | 18.40 | 59.51 | 3.54 | 41.52 | 3.33 | 8.50 |

| | | | | | | | | | |
|---|---|---|---|---|---|---|---|---|---|
| B5701 | 1976 | Hulled | 92 | 17.06 | 57.87 | 4.75 | 28.78 | 2.81 | 11.14 |
| B5704 | 1976 | Hulled | 74 | 17.03 | 58.67 | 3.95 | 38.87 | 3.19 | 9.59 |
| B5705 | 1976 | Hulled | 50 | 17.87 | 58.36 | 3.88 | 43.87 | 3.33 | 9.05 |
| B5706 | 1976 | Hulled | 40 | 19.92 | 58.84 | 3.80 | 40.10 | 3.33 | 9.59 |
| B5707 | 1976 | Hulled | 52 | 17.44 | 58.88 | 4.02 | 40.17 | 3.30 | 9.27 |
| B5711 | 1976 | Hulled | 60 | 19.53 | 58.63 | 3.84 | 38.60 | 3.33 | 8.73 |
| B5718 | 1976 | Hulled | 60 | 17.13 | 59.57 | 3.64 | 37.80 | 3.36 | 8.64 |
| B5722 | 1976 | Hulled | 26 | 21.03 | 57.40 | 5.49 | 40.69 | 3.34 | 10.60 |
| B5723 | 1976 | Hulled | 94 | 16.22 | 58.75 | 4.54 | 35.72 | 3.27 | 9.57 |
| B5739 | 1976 | Hulled | 74 | 18.10 | 60.43 | 3.74 | 33.39 | 3.23 | 8.95 |
| B5747 | 1976 | Hulled | 8 | 21.80 | 55.82 | 5.17 | 42.21 | 3.66 | 10.34 |
| B5748 | 1976 | Hulled | 70 | 20.70 | 56.18 | 5.68 | 37.19 | 3.55 | 9.69 |
| B5750 | 1976 | Hulled | 70 | 20.94 | 56.74 | 5.42 | 39.40 | 3.63 | 10.77 |
| B5752 | 1976 | Hulled | 64 | 19.40 | 56.02 | 5.86 | 30.89 | 3.13 | 11.64 |
| B5789 | 1976 | Hulled | 84 | 18.78 | 57.86 | 4.51 | 52.62 | 3.51 | 10.40 |
| B5792 | 1976 | Hulled | 40 | 15.42 | 60.25 | 3.73 | 48.86 | 3.48 | 10.13 |
| B5804 | 1976 | Hulled | 90 | 17.41 | 58.95 | 4.07 | 28.08 | 3.03 | 8.42 |
| B5821 | 1976 | Hulled | 88 | 11.35 | 62.70 | 3.00 | 36.29 | 3.29 | 9.27 |
| B5839 | 1976 | Hulled | 76 | 16.91 | 59.42 | 3.79 | 33.38 | 3.13 | 9.63 |
| B5841 | 1976 | Hulled | 24 | 12.43 | 60.01 | 2.94 | 32.26 | 3.02 | 8.41 |
| B5842 | 1976 | Hulled | 36 | 15.57 | 59.54 | 2.75 | 39.08 | 3.20 | 8.63 |
| B5843 | 1976 | Hulled | 14 | 15.03 | 59.13 | 3.29 | 36.06 | 3.17 | 8.22 |
| B5844 | 1976 | Hulled | 6 | 15.74 | 64.53 | 1.75 | 34.08 | 3.59 | 7.35 |
| B5845 | 1976 | Hulled | 6 | 15.17 | 65.32 | 1.91 | 33.60 | 3.51 | 7.37 |
| B5846 | 1976 | Hulled | 10 | 13.50 | 60.39 | 2.62 | 35.64 | 3.18 | 7.73 |
| B5852 | 1976 | Hulled | 38 | 18.48 | 58.07 | 5.38 | 42.03 | 3.52 | 11.43 |
| B5853 | 1976 | Hulled | 60 | 18.94 | 58.48 | 4.81 | 41.29 | 3.42 | 10.98 |

| | | | | | | | | | |
|---|---|---|---|---|---|---|---|---|---|
| B5854 | 1976 | Hulled | 52 | 17.14 | 57.72 | 5.25 | 41.34 | 3.40 | 11.44 |
| B5856 | 1976 | Hulled | 62 | 18.89 | 57.59 | 5.08 | 42.05 | 3.41 | 11.36 |
| B5857 | 1976 | Hulled | 64 | 19.44 | 56.99 | 5.69 | 42.16 | 3.41 | 12.11 |
| B5864 | 1976 | Hulled | 36 | 18.77 | 59.99 | 4.31 | 39.14 | 3.40 | 9.48 |
| B5869 | 1976 | Hulled | 2 | 18.60 | 66.28 | 2.00 | 31.12 | 3.60 | 8.16 |
| B5872 | 1976 | Hulled | 86 | 18.05 | 58.43 | 5.35 | 38.71 | 3.27 | 11.29 |
| B5874 | 1976 | Hulled | 88 | 18.71 | 57.65 | 5.21 | 44.57 | 3.44 | 11.56 |
| B5875 | 1976 | Hulled | 64 | 21.22 | 56.66 | 6.42 | 36.53 | 3.28 | 13.76 |
| B5876 | 1976 | Hulled | 84 | 19.17 | 57.78 | 5.18 | 36.93 | 3.21 | 11.22 |
| B5879 | 1976 | Hulled | 66 | 22.08 | 57.64 | 6.40 | 35.05 | 3.32 | 11.64 |
| B5880 | 1976 | Hulled | 96 | 20.42 | 56.45 | 5.78 | 35.48 | 3.19 | 11.40 |
| B5884 | 1976 | Hulled | 60 | 19.30 | 58.06 | 4.82 | 35.82 | 3.18 | 11.84 |
| B5885 | 1976 | Hulled | 48 | 18.72 | 57.79 | 5.05 | 37.10 | 3.22 | 11.80 |
| B5887 | 1976 | Hulled | 48 | 20.66 | 57.28 | 5.34 | 33.24 | 3.03 | 11.28 |
| B5891 | 1976 | Hulled | 36 | 20.26 | 58.45 | 4.34 | 33.42 | 3.22 | 8.42 |
| B5892 | 1976 | Hulled | 34 | 19.90 | 58.84 | 4.28 | 34.61 | 3.24 | 8.40 |
| B5893 | 1976 | Hulled | 22 | 20.45 | 58.89 | 4.38 | 33.09 | 3.19 | 8.85 |
| B5894 | 1976 | Hulled | 44 | 19.53 | 57.38 | 5.24 | 48.63 | 3.67 | 11.24 |
| B5896 | 1976 | Hulled | 42 | 19.66 | 56.52 | 5.08 | 47.17 | 3.63 | 11.17 |
| B5898 | 1976 | Hulled | 30 | 19.56 | 57.18 | 5.08 | 48.17 | 3.66 | 11.22 |
| B5899 | 1976 | Hulled | 40 | 19.67 | 58.28 | 4.48 | 51.41 | 3.70 | 11.28 |
| B5900 | 1976 | Hulled | 46 | 20.02 | 58.19 | 4.74 | 48.69 | 3.64 | 11.25 |
| B5901 | 1976 | Hulled | 40 | 20.50 | 57.73 | 5.03 | 49.10 | 3.69 | 11.12 |
| B5902 | 1976 | Hulled | 70 | 18.87 | 57.81 | 4.71 | 32.66 | 3.13 | 10.43 |
| B5903 | 1976 | Hulled | 64 | 19.64 | 58.16 | 4.69 | 36.36 | 3.21 | 10.86 |
| B5904 | 1976 | Hulled | 72 | 19.73 | 58.37 | 4.59 | 32.61 | 3.11 | 10.53 |
| B5239 | 1976 | Hulled | 60 | 14.68 | 56.67 | 6.63 | 46.25 | 3.65 | 9.81 |

| | | | | | | | | | |
|---|---|---|---|---|---|---|---|---|---|
| B5240 | 1976 | Hulled | 26 | 18.90 | 58.55 | 4.95 | 46.62 | 3.53 | 9.31 |
| B5241 | 1976 | Hulled | 86 | 19.35 | 59.11 | 3.92 | 35.96 | 3.30 | 8.45 |
| B5246 | 1976 | Hulled | 40 | 20.68 | 58.23 | 4.37 | 44.78 | 3.53 | 9.41 |
| B5247 | 1976 | Hulled | 60 | 18.99 | 59.00 | 4.19 | 32.29 | 3.13 | 8.49 |
| B5248 | 1976 | Hulled | 52 | 21.21 | 57.89 | 4.35 | 39.90 | 3.51 | 8.37 |
| B5250 | 1976 | Hulled | 30 | 20.18 | 58.96 | 4.18 | 38.35 | 3.26 | 9.56 |
| B5251 | 1976 | Hulled | 36 | 20.26 | 59.46 | 3.89 | 32.92 | 3.17 | 8.51 |
| B5252 | 1976 | Hulled | 22 | 19.62 | 59.13 | 4.09 | 44.85 | 3.62 | 8.57 |
| B5253 | 1976 | Hulled | 72 | 19.45 | 60.60 | 3.65 | 37.38 | 3.53 | 8.03 |
| B5254 | 1976 | Hulled | 58 | 19.95 | 59.06 | 4.01 | 43.57 | 3.40 | 9.71 |
| B5255 | 1976 | Hulled | 86 | 17.21 | 59.03 | 4.47 | 34.96 | 3.23 | 8.57 |
| B5258 | 1976 | Hulled | 70 | 20.61 | 57.78 | 4.30 | 43.12 | 3.41 | 9.28 |
| B5259 | 1976 | Hulled | 60 | 20.03 | 58.89 | 3.93 | 38.40 | 3.31 | 8.66 |
| B5260 | 1976 | Hulled | 14 | 20.15 | 58.70 | 3.97 | 44.98 | 3.54 | 9.66 |
| B5261 | 1976 | Hulled | 12 | 19.10 | 58.35 | 4.24 | 39.24 | 3.36 | 8.49 |
| B5262 | 1976 | Hulled | 82 | 20.53 | 59.03 | 4.00 | 34.84 | 3.17 | 8.79 |
| B5263 | 1976 | Hulled | 32 | 19.02 | 58.12 | 4.22 | 40.41 | 3.48 | 8.78 |
| B5264 | 1976 | Hulled | 26 | 18.88 | 59.13 | 4.14 | 46.75 | 3.63 | 10.42 |
| B5265 | 1976 | Hulled | 22 | 20.51 | 57.63 | 4.66 | 44.44 | 3.50 | 12.24 |
| B5267 | 1976 | Hulled | 36 | 17.60 | 57.03 | 5.09 | 44.80 | 3.47 | 8.96 |
| B5268 | 1976 | Hulled | 66 | 19.66 | 59.04 | 3.93 | 37.59 | 3.25 | 8.77 |
| B5269 | 1976 | Hulled | 4 | 18.90 | 59.00 | 4.15 | 45.71 | 3.40 | 11.26 |
| B5270 | 1976 | Hulled | 38 | 18.03 | 57.80 | 5.49 | 33.83 | 3.14 | 8.33 |
| B5271 | 1976 | Hulled | 68 | 19.60 | 59.28 | 3.79 | 37.30 | 3.25 | 8.47 |
| B5272 | 1976 | Hulled | 80 | 21.21 | 58.06 | 4.48 | 34.79 | 3.14 | 8.77 |
| B5273 | 1976 | Hulled | 26 | 18.96 | 59.30 | 3.90 | 40.38 | 3.43 | 8.13 |
| B5274 | 1976 | Hulled | 24 | 20.18 | 61.29 | 2.90 | 45.53 | 3.44 | 8.92 |

| | | | | | | | | | |
|---|---|---|---|---|---|---|---|---|---|
| B5275 | 1976 | Hulled | 66 | 20.28 | 58.81 | 4.03 | 37.12 | 3.18 | 10.31 |
| B5276 | 1976 | Hulled | 60 | 19.37 | 57.64 | 5.29 | 42.12 | 3.40 | 11.58 |
| B5277 | 1976 | Hulled | 68 | 20.51 | 57.24 | 4.98 | 41.90 | 3.38 | 8.79 |
| B5278 | 1976 | Hulled | 52 | 19.39 | 58.06 | 4.44 | 41.31 | 3.46 | 8.93 |
| B5279 | 1976 | Hulled | 92 | 19.89 | 57.93 | 4.35 | 31.41 | 3.05 | 8.41 |
| B5280 | 1976 | Hulled | 52 | 19.99 | 59.03 | 3.80 | 41.07 | 3.43 | 8.89 |
| B5281 | 1976 | Hulled | 38 | 19.90 | 58.22 | 4.26 | 40.58 | 3.43 | 9.51 |
| B5282 | 1976 | Hulled | 66 | 19.67 | 59.27 | 4.00 | 41.09 | 3.47 | 9.48 |
| B5283 | 1976 | Hulled | 68 | 20.11 | 58.75 | 4.50 | 44.61 | 3.39 | 9.79 |
| B5284 | 1976 | Hulled | 30 | 18.69 | 59.32 | 4.10 | 40.56 | 3.39 | 9.80 |
| B5285 | 1976 | Hulled | 28 | 20.21 | 59.26 | 4.37 | 45.12 | 3.48 | 9.32 |
| B5286 | 1976 | Hulled | 44 | 20.19 | 58.28 | 4.24 | 44.48 | 3.67 | 9.39 |
| B5287 | 1976 | Hulled | 54 | 18.97 | 58.91 | 4.22 | 38.65 | 3.25 | 10.61 |
| B5288 | 1976 | Hulled | 48 | 18.55 | 57.97 | 4.30 | 45.30 | 3.56 | 9.11 |
| B5289 | 1976 | Hulled | 28 | 16.80 | 59.38 | 3.97 | 46.73 | 3.60 | 8.71 |
| B5290 | 1976 | Hulled | 18 | 17.77 | 60.53 | 3.70 | 39.80 | 3.45 | 8.82 |
| B5291 | 1976 | Hulled | 44 | 18.07 | 60.90 | 3.66 | 42.18 | 3.46 | 9.13 |
| B5294 | 1976 | Hulled | 22 | 18.51 | 58.65 | 4.36 | 44.92 | 3.44 | 10.44 |
| B5295 | 1976 | Hulled | 44 | 19.81 | 59.85 | 4.12 | 38.75 | 3.26 | 9.80 |
| B5296 | 1976 | Hulled | 76 | 18.06 | 59.44 | 4.37 | 40.98 | 3.34 | 9.44 |
| B5297 | 1976 | Hulled | 58 | 18.82 | 58.39 | 4.60 | 49.16 | 3.72 | 9.05 |
| B5298 | 1976 | Hulled | 72 | 19.43 | 58.58 | 4.08 | 45.44 | 3.55 | 9.74 |
| B5301 | 1976 | Hulled | 30 | 17.41 | 58.65 | 4.55 | 42.47 | 3.46 | 9.56 |
| B5303 | 1976 | Hulled | 68 | 18.50 | 59.84 | 4.22 | 28.34 | 2.89 | 9.22 |
| B5304 | 1976 | Hulled | 46 | 15.19 | 59.15 | 3.71 | 32.40 | 3.03 | 9.22 |
| B5307 | 1976 | Hulled | 84 | 14.85 | 59.41 | 3.90 | 35.70 | 3.32 | 8.62 |
| B5316 | 1976 | Hulled | 46 | 21.37 | 57.99 | 3.96 | 40.30 | 3.60 | 8.42 |

| | | | | | | | | | |
|---|---|---|---|---|---|---|---|---|---|
| B5319 | 1976 | Hulled | 86 | 20.00 | 59.22 | 4.16 | 22.26 | 2.80 | 8.62 |
| B5324 | 1976 | Hulled | 100 | 19.15 | 58.04 | 4.74 | 25.58 | 2.98 | 8.46 |
| B5326 | 1976 | Hulled | 74 | 18.80 | 58.50 | 4.69 | 26.26 | 3.01 | 9.42 |
| B5328 | 1976 | Hulled | 90 | 18.14 | 57.85 | 5.82 | 34.40 | 3.33 | 8.67 |
| B5330 | 1976 | Hulled | 86 | 19.68 | 59.73 | 4.13 | 30.78 | 3.13 | 9.23 |
| B5339 | 1976 | Hulled | 92 | 18.99 | 59.08 | 4.34 | 29.28 | 3.17 | 8.03 |
| B5348 | 1976 | Hulled | 48 | 22.59 | 55.35 | 4.96 | 35.16 | 3.29 | 9.02 |
| B5349 | 1976 | Hulled | 84 | 20.03 | 58.94 | 4.77 | 34.88 | 3.28 | 8.08 |
| B5444 | 1976 | Hulled | 84 | 19.61 | 56.42 | 5.00 | 25.40 | 2.73 | 8.81 |
| B5445 | 1976 | Hulled | 88 | 15.30 | 59.97 | 4.27 | 31.58 | 3.01 | 8.40 |
| B5446 | 1976 | Hulled | 74 | 14.44 | 59.57 | 4.17 | 38.91 | 3.32 | 8.75 |
| B5447 | 1976 | Hulled | 52 | 14.87 | 59.31 | 4.23 | 33.22 | 3.01 | 8.69 |
| B5448 | 1976 | Hulled | 42 | 15.96 | 59.10 | 4.08 | 29.28 | 2.80 | 8.42 |
| B5449 | 1976 | Hulled | 78 | 16.18 | 58.41 | 4.28 | 34.88 | 3.05 | 9.22 |
| B5450 | 1976 | Hulled | 72 | 15.22 | 59.45 | 4.16 | 38.15 | 3.07 | 9.49 |
| B5451 | 1976 | Hulled | 44 | 16.08 | 58.65 | 4.19 | 40.80 | 3.29 | 9.46 |
| B5452 | 1976 | Hulled | 74 | 16.17 | 60.71 | 3.90 | 31.86 | 2.92 | 8.54 |
| B5453 | 1976 | Hulled | 80 | 16.83 | 58.63 | 4.37 | 30.10 | 3.09 | 8.27 |
| B5454 | 1976 | Hulled | 90 | 16.31 | 58.62 | 4.44 | 28.61 | 3.03 | 8.26 |
| B5455 | 1976 | Hulled | 76 | 16.19 | 58.98 | 4.60 | 34.07 | 3.19 | 8.41 |
| B5456 | 1976 | Hulled | 86 | 14.58 | 59.79 | 3.96 | 27.03 | 2.93 | 8.51 |
| B5457 | 1976 | Hulled | 82 | 14.45 | 60.41 | 4.18 | 26.00 | 2.88 | 8.45 |
| B5458 | 1976 | Hulled | 82 | 15.07 | 60.45 | 4.26 | 30.87 | 3.00 | 8.80 |
| B5459 | 1976 | Hulled | 90 | 16.58 | 58.87 | 4.01 | 26.70 | 2.97 | 7.85 |
| B5460 | 1976 | Hulled | 82 | 16.13 | 59.44 | 4.22 | 28.32 | 2.91 | 10.12 |
| B5461 | 1976 | Hulled | 80 | 15.46 | 59.16 | 4.72 | 36.57 | 3.10 | 9.15 |
| B5462 | 1976 | Hulled | 74 | 15.28 | 59.64 | 3.77 | 37.58 | 3.11 | 9.06 |

| | | | | | | | | | |
|---|---|---|---|---|---|---|---|---|---|
| B5463 | 1976 | Hulled | 54 | 17.10 | 58.38 | 4.18 | 32.73 | 2.91 | 9.48 |
| B5464 | 1976 | Hulled | 86 | 16.13 | 59.36 | 4.28 | 33.52 | 3.02 | 9.43 |
| B5465 | 1976 | Hulled | 58 | 15.48 | 58.90 | 4.41 | 36.56 | 3.23 | 8.90 |
| B5466 | 1976 | Hulled | 84 | 14.86 | 60.31 | 3.78 | 40.17 | 3.37 | 8.94 |
| B5468 | 1976 | Hulled | 18 | 14.31 | 61.04 | 3.65 | 32.18 | 3.22 | 7.01 |
| B5469 | 1976 | Hulled | 88 | 18.08 | 65.13 | 2.11 | 34.33 | 3.08 | 9.51 |
| B5470 | 1976 | Hulled | 8 | 16.61 | 59.46 | 4.10 | 27.75 | 3.08 | 6.64 |
| B5471 | 1976 | Hulled | 82 | 18.02 | 65.63 | 2.05 | 28.37 | 2.91 | 8.76 |
| B5472 | 1976 | Hulled | 94 | 14.90 | 59.27 | 4.17 | 30.08 | 2.98 | 8.88 |
| B5473 | 1976 | Hulled | 90 | 15.66 | 59.21 | 4.40 | 27.00 | 2.86 | 8.87 |
| B5474 | 1976 | Hulled | 80 | 15.48 | 59.22 | 4.23 | 29.04 | 2.86 | 8.89 |
| B5475 | 1976 | Hulled | 74 | 15.43 | 58.67 | 4.19 | 32.68 | 3.03 | 9.06 |
| B5476 | 1976 | Hulled | 50 | 14.98 | 59.71 | 3.94 | 35.54 | 3.12 | 9.59 |
| B5478 | 1976 | Hulled | 82 | 15.30 | 59.27 | 4.03 | 31.83 | 3.07 | 8.46 |
| B5479 | 1976 | Hulled | 66 | 15.13 | 59.77 | 4.02 | 34.29 | 3.02 | 9.27 |
| B5480 | 1976 | Hulled | 76 | 17.01 | 58.84 | 4.07 | 35.54 | 3.13 | 9.05 |
| B5481 | 1976 | Hulled | 60 | 16.15 | 59.25 | 4.02 | 34.71 | 3.14 | 8.54 |
| B5482 | 1976 | Hulled | 86 | 14.72 | 60.12 | 3.75 | 34.24 | 3.06 | 8.94 |
| B5483 | 1976 | Hulled | 78 | 15.19 | 58.94 | 4.32 | 32.60 | 3.09 | 8.62 |
| B5484 | 1976 | Hulled | 82 | 15.60 | 60.61 | 3.49 | 32.69 | 3.18 | 8.64 |
| B5485 | 1976 | Hulled | 18 | 14.64 | 60.84 | 3.45 | 25.55 | 2.97 | 6.37 |
| B5486 | 1976 | Hulled | 74 | 15.44 | 68.25 | 2.29 | 35.12 | 3.16 | 8.49 |
| B18314 | 1979 | Hulled | 70 | 14.24 | 68.61 | 1.83 | 42.96 | 3.56 | 7.92 |
| B18324 | 1979 | Hulled | 76 | 13.59 | 64.65 | 2.76 | 43.79 | 3.69 | 8.13 |
| B18317 | 1979 | Hulled | 96 | 13.16 | 65.70 | 2.91 | 47.23 | 3.65 | 8.29 |
| B18291 | 1979 | Hulled | 68 | 13.17 | 63.22 | 3.34 | 41.68 | 3.56 | 8.13 |
| B17899 | 1979 | Hulled | 6 | 14.47 | 58.61 | 3.38 | 43.60 | 3.70 | 9.71 |

| | | | | | | | | | |
|---|---|---|---|---|---|---|---|---|---|
| B18149 | 1979 | Hulled | 4 | 18.53 | 57.29 | 3.41 | 44.37 | 3.57 | 10.11 |
| B17920 | 1979 | Hulled | 4 | 16.86 | 56.14 | 3.42 | 34.34 | 3.09 | 9.38 |
| B17855 | 1979 | Hulled | 10 | 16.84 | 58.53 | 3.47 | 47.06 | 3.64 | 10.08 |
| B17925 | 1979 | Hulled | 6 | 14.66 | 57.70 | 3.56 | 41.08 | 3.33 | 10.49 |
| B17740 | 1979 | Hulled | 4 | 15.63 | 59.56 | 3.59 | 35.05 | 3.23 | 10.00 |
| B17980 | 1979 | Hulled | 12 | 16.31 | 57.68 | 3.62 | 37.35 | 3.27 | 9.46 |
| B18138 | 1979 | Hulled | 18 | 18.80 | 58.57 | 3.63 | 39.28 | 3.50 | 9.70 |
| B18292 | 1979 | Hulled | 64 | 12.19 | 63.85 | 3.65 | 39.71 | 3.53 | 7.97 |
| B17712 | 1979 | Hulled | 2 | 16.24 | 71.68 | 3.65 | 37.79 | 3.44 | 8.75 |
| B18222 | 1979 | Hulled | 18 | 15.14 | 60.36 | 3.68 | 46.97 | 3.72 | 8.71 |
| B17892 | 1979 | Hulled | 10 | 15.66 | 56.83 | 3.70 | 46.55 | 3.84 | 10.43 |
| B18013 | 1979 | Hulled | 18 | 16.65 | 56.42 | 3.71 | 41.33 | 3.39 | 10.43 |
| B17896 | 1979 | Hulled | 32 | 14.41 | 60.67 | 3.79 | 37.36 | 3.29 | 10.20 |
| B17834 | 1979 | Hulled | 10 | 19.05 | 54.32 | 3.79 | 47.87 | 3.69 | 11.27 |
| B18136 | 1979 | Hulled | 0 | 18.04 | 57.25 | 3.86 | 45.24 | 3.57 | 10.36 |
| B18119 | 1979 | Hulled | 26 | 16.01 | 57.38 | 3.95 | 37.15 | 3.41 | 9.93 |
| B18127 | 1979 | Hulled | 18 | 15.50 | 58.12 | 3.98 | 33.62 | 3.17 | 9.50 |
| B17854 | 1979 | Hulled | 40 | 15.28 | 57.64 | 4.00 | 43.05 | 3.50 | 10.24 |
| B17984 | 1979 | Hulled | 34 | 13.55 | 60.39 | 4.03 | 45.59 | 3.61 | 10.66 |
| B18120 | 1979 | Hulled | 12 | 16.49 | 58.71 | 4.05 | 43.49 | 3.63 | 10.48 |
| B18125 | 1979 | Hulled | 18 | 16.56 | 56.35 | 4.06 | 37.74 | 3.26 | 10.44 |
| B18037 | 1979 | Hulled | 24 | 15.06 | 57.32 | 4.08 | 34.26 | 3.16 | 9.45 |
| B18122 | 1979 | Hulled | 8 | 15.72 | 57.13 | 4.09 | 34.35 | 3.19 | 10.15 |
| B17879 | 1979 | Hulled | 36 | 14.60 | 56.72 | 4.10 | 35.69 | 3.27 | 9.40 |
| B17860 | 1979 | Hulled | 38 | 17.41 | 54.75 | 4.11 | 46.00 | 3.46 | 10.84 |
| B18137 | 1979 | Hulled | 28 | 17.03 | 56.66 | 4.13 | 45.30 | 3.58 | 10.58 |
| B18110 | 1979 | Hulled | 28 | 14.67 | 54.86 | 4.13 | 40.28 | 3.49 | 9.70 |

| | | | | | | | | | |
|---|---|---|---|---|---|---|---|---|---|
| B18048 | 1979 | Hulled | 16 | 15.19 | 54.42 | 4.16 | 36.39 | 3.27 | 10.07 |
| B17850 | 1979 | Hulled | 12 | 18.31 | 54.42 | 4.16 | 42.08 | 3.50 | 10.38 |
| B17849 | 1979 | Hulled | 18 | 18.77 | 55.47 | 4.17 | 41.27 | 3.43 | 10.40 |
| B17926 | 1979 | Hulled | 6 | 14.18 | 57.04 | 4.17 | 35.71 | 3.28 | 9.81 |
| B17893 | 1979 | Hulled | 48 | 15.45 | 54.59 | 4.18 | 31.20 | 3.07 | 9.67 |
| B17805 | 1979 | Hulled | 22 | 14.84 | 56.32 | 4.18 | 34.83 | 3.20 | 9.35 |
| B17835 | 1979 | Hulled | 18 | 17.47 | 54.32 | 4.21 | 44.20 | 3.57 | 10.93 |
| B18163 | 1979 | Hulled | 10 | 15.61 | 56.75 | 4.22 | 38.60 | 3.33 | 10.83 |
| B17993 | 1979 | Hulled | 8 | 16.42 | 57.63 | 4.24 | 34.18 | 3.24 | 9.71 |
| B18132 | 1979 | Hulled | 8 | 14.27 | 58.95 | 4.24 | 36.72 | 3.35 | 9.50 |
| B17857 | 1979 | Hulled | 42 | 16.34 | 56.09 | 4.27 | 45.43 | 3.59 | 10.91 |
| B17853 | 1979 | Hulled | 32 | 17.58 | 65.24 | 4.27 | 47.12 | 3.63 | 10.93 |
| B17671 | 1979 | Hulled | 0 | 15.59 | 57.22 | 4.29 | 41.33 | 3.47 | 10.22 |
| B17988 | 1979 | Hulled | 24 | 15.84 | 55.19 | 4.30 | 34.74 | 3.20 | 9.95 |
| B18111 | 1979 | Hulled | 16 | 16.43 | 56.40 | 4.34 | 32.61 | 3.18 | 9.62 |
| B18140 | 1979 | Hulled | 14 | 15.26 | 55.97 | 4.36 | 32.83 | 3.23 | 9.85 |
| B17814 | 1979 | Hulled | 22 | 18.30 | 57.38 | 4.37 | 41.19 | 3.41 | 11.31 |
| B18124 | 1979 | Hulled | 18 | 14.15 | 58.06 | 4.37 | 34.17 | 3.21 | 10.09 |
| B17821 | 1979 | Hulled | 20 | 16.89 | 59.44 | 4.38 | 43.39 | 3.70 | 10.31 |
| B18340 | 1979 | Hulled | 38 | 16.28 | 57.72 | 4.38 | 31.02 | 3.31 | 9.16 |
| B17976 | 1979 | Hulled | 2 | 14.32 | 55.49 | 4.39 | 32.28 | 3.04 | 9.90 |
| B17908 | 1979 | Hulled | 18 | 14.70 | 58.68 | 4.42 | 30.98 | 3.16 | 9.29 |
| B17817 | 1979 | Hulled | 40 | 12.74 | 59.37 | 4.42 | 36.10 | 3.23 | 9.69 |
| B17690 | 1979 | Hulled | 0 | 15.02 | 58.81 | 4.48 | 39.43 | 3.31 | 10.51 |
| B17825 | 1979 | Hulled | 34 | 15.20 | 55.24 | 4.51 | 30.06 | 3.00 | 10.47 |
| B17856 | 1979 | Hulled | 22 | 16.88 | 56.92 | 4.51 | 44.27 | 3.48 | 11.20 |
| B17918 | 1979 | Hulled | 42 | 15.15 | 56.99 | 4.56 | 30.58 | 2.95 | 10.28 |

| | | | | | | | | | |
|---|---|---|---|---|---|---|---|---|---|
| B18121 | 1979 | Hulled | 12 | 15.56 | 56.74 | 4.56 | 42.89 | 3.46 | 11.56 |
| B18118 | 1979 | Hulled | 4 | 16.53 | 54.89 | 4.57 | 39.27 | 3.34 | 10.93 |
| B17809 | 1979 | Hulled | 18 | 15.71 | 55.88 | 4.58 | 44.03 | 3.66 | 12.68 |
| B18020 | 1979 | Hulled | 8 | 13.53 | 57.13 | 4.58 | 40.89 | 3.45 | 11.24 |
| B18114 | 1979 | Hulled | 20 | 14.78 | 59.00 | 4.58 | 40.74 | 3.46 | 9.97 |
| B17713 | 1979 | Hulled | 0 | 15.21 | 57.71 | 4.58 | 37.98 | 3.22 | 10.28 |
| B17927 | 1979 | Hulled | 8 | 15.26 | 54.23 | 4.60 | 34.70 | 3.26 | 10.03 |
| B18028 | 1979 | Hulled | 12 | 15.27 | 56.69 | 4.60 | 35.34 | 3.24 | 9.96 |
| B18311 | 1979 | Hulled | 76 | 15.52 | 58.14 | 4.60 | 42.31 | 3.56 | 8.49 |
| B18036 | 1979 | Hulled | 22 | 15.93 | 62.49 | 4.62 | 36.03 | 3.32 | 9.87 |
| B17901 | 1979 | Hulled | 2 | 16.39 | 65.05 | 4.64 | 43.46 | 3.48 | 11.48 |
| B18182 | 1979 | Hulled | 56 | 15.26 | 57.22 | 4.67 | 50.19 | 3.88 | 9.11 |
| B17692 | 1979 | Hulled | 0 | 13.85 | 60.00 | 4.69 | 38.08 | 3.39 | 9.57 |
| B18157 | 1979 | Hulled | 8 | 15.02 | 55.89 | 4.69 | 31.10 | 3.12 | 9.80 |
| B18112 | 1979 | Hulled | 38 | 15.43 | 58.39 | 4.69 | 36.68 | 3.35 | 9.69 |
| B17985 | 1979 | Hulled | 18 | 17.22 | 56.16 | 4.70 | 34.13 | 3.13 | 10.58 |
| B18032 | 1979 | Hulled | 24 | 14.61 | 57.20 | 4.70 | 38.21 | 3.35 | 10.48 |
| B18162 | 1979 | Hulled | 4 | 17.54 | 58.77 | 4.70 | 37.09 | 3.43 | 10.15 |
| B18159 | 1979 | Hulled | 8 | 15.17 | 56.28 | 4.71 | 29.46 | 3.03 | 10.04 |
| B17703 | 1979 | Hulled | 0 | 15.36 | 56.39 | 4.74 | 43.07 | 3.54 | 10.41 |
| B17684 | 1979 | Hulled | 2 | 15.94 | 65.32 | 4.76 | 43.50 | 3.58 | 9.79 |
| B17820 | 1979 | Hulled | 10 | 17.41 | 61.55 | 4.77 | 38.07 | 3.58 | 10.23 |
| B18144 | 1979 | Hulled | 40 | 14.35 | 56.65 | 4.78 | 38.99 | 3.37 | 10.19 |
| B17823 | 1979 | Hulled | 18 | 13.40 | 54.97 | 4.82 | 34.02 | 3.28 | 9.92 |
| B18051 | 1979 | Hulled | 30 | 15.86 | 54.07 | 4.84 | 40.14 | 3.38 | 9.91 |
| B17699 | 1979 | Hulled | 0 | 15.48 | 56.76 | 4.85 | 40.15 | 3.39 | 10.64 |
| B17977 | 1979 | Hulled | 10 | 14.38 | 56.46 | 4.90 | 31.81 | 3.00 | 10.03 |

| | | | | | | | | | |
|---|---|---|---|---|---|---|---|---|---|
| B17743 | 1979 | Hulled | 0 | 17.23 | 59.28 | 4.92 | 39.95 | 3.45 | 10.05 |
| B17975 | 1979 | Hulled | 30 | 14.52 | 60.25 | 4.94 | 30.82 | 3.04 | 9.39 |
| B18007 | 1979 | Hulled | 36 | 14.24 | 62.54 | 4.95 | 33.05 | 3.18 | 9.52 |
| B17973 | 1979 | Hulled | 10 | 16.21 | 66.67 | 4.96 | 44.52 | 3.64 | 11.03 |
| B17803 | 1979 | Hulled | 32 | 15.01 | 59.01 | 4.97 | 33.31 | 3.16 | 10.68 |
| B18024 | 1979 | Hulled | 0 | 15.53 | 67.01 | 4.97 | 32.50 | 3.12 | 10.28 |
| B17811 | 1979 | Hulled | 14 | 19.48 | 51.20 | 5.03 | 39.82 | 3.51 | 12.01 |
| B17979 | 1979 | Hulled | 36 | 14.18 | 59.82 | 5.04 | 27.04 | 2.98 | 9.72 |
| B17802 | 1979 | Hulled | 22 | 15.24 | 60.87 | 5.05 | 35.82 | 3.21 | 11.09 |
| B17678 | 1979 | Hulled | 0 | 16.08 | 53.09 | 5.05 | 45.77 | 3.56 | 10.22 |
| B17696 | 1979 | Hulled | 0 | 14.33 | 57.84 | 5.09 | 44.39 | 3.62 | 11.08 |
| B17966 | 1979 | Hulled | 18 | 14.72 | 53.55 | 5.11 | 29.70 | 2.93 | 10.45 |
| B17863 | 1979 | Hulled | 18 | 14.90 | 62.90 | 5.14 | 33.98 | 3.06 | 10.73 |
| B17822 | 1979 | Hulled | 20 | 14.29 | 64.13 | 5.14 | 34.87 | 3.15 | 10.77 |
| B18019 | 1979 | Hulled | 0 | 14.71 | 54.12 | 5.14 | 42.09 | 3.67 | 11.01 |
| B17905 | 1979 | Hulled | 32 | 14.64 | 55.76 | 5.19 | 31.78 | 3.06 | 10.66 |
| B18004 | 1979 | Hulled | 0 | 16.94 | 65.73 | 5.24 | 41.24 | 3.47 | 10.74 |
| B18142 | 1979 | Hulled | 10 | 14.64 | 56.39 | 5.26 | 33.48 | 3.34 | 9.98 |
| B18164 | 1979 | Hulled | 22 | 14.95 | 61.13 | 5.26 | 29.57 | 3.12 | 9.53 |
| B17827 | 1979 | Hulled | 10 | 16.17 | 60.59 | 5.32 | 41.94 | 3.42 | 11.20 |
| B17804 | 1979 | Hulled | 38 | 15.55 | 56.54 | 5.36 | 31.67 | 3.19 | 10.88 |
| B17715 | 1979 | Hulled | 0 | 14.72 | 58.86 | 5.39 | 32.95 | 3.15 | 9.90 |
| B17865 | 1979 | Hulled | 60 | 13.82 | 53.82 | 5.55 | 35.00 | 3.05 | 11.27 |
| B17930 | 1979 | Hulled | 10 | 15.81 | 53.30 | 5.65 | 34.69 | 3.34 | 10.90 |
| B17708 | 1979 | Hulled | 2 | 14.72 | 65.52 | 5.75 | 43.61 | 3.58 | 12.01 |
| B17488 | 1979 | Hulled | 18 | 15.81 | 57.75 | 6.20 | 49.86 | 3.89 | 11.44 |
| B17916 | 1979 | Hulled | 2 | 17.89 | 93.57 | 6.23 | 41.28 | 3.80 | 9.62 |

| | | | | | | | | | |
|---|---|---|---|---|---|---|---|---|---|
| B18012 | 1979 | Hulled | 12 | 14.47 | 58.59 | 6.33 | 33.35 | 3.27 | 10.27 |
| B17961 | 1979 | Hulled | 30 | 17.40 | 93.50 | 6.40 | 32.45 | 3.24 | 10.41 |
| B18015 | 1979 | Hulled | 18 | 13.82 | 57.62 | 6.66 | 35.71 | 3.37 | 10.53 |
| B17793 | 1979 | Hulled | 26 | 17.65 | 97.59 | 6.82 | 39.09 | 3.40 | 10.07 |
| B17991 | 1979 | Hulled | 6 | 18.00 | 90.20 | 6.90 | 35.29 | 3.44 | 9.83 |
| B4017 | 2003 | Hulled | 98 | 15.07 | 49.64 | 7.12 | 43.37 | 3.35 | 13.44 |
| B5090 | 2003 | Hulled | 96 | 16.85 | 50.56 | 6.64 | 25.26 | 3.04 | 9.91 |
| B4507 | 2003 | Hulled | 62 | 17.80 | 52.37 | 5.45 | 43.26 | 3.53 | 11.77 |
| B5103 | 2003 | Hulled | 98 | 17.30 | 52.52 | 6.28 | 33.46 | 2.99 | 11.14 |
| B4575 | 2003 | Hulled | 94 | 15.37 | 52.96 | 7.06 | 39.44 | 3.40 | 12.11 |
| B4661 | 2003 | Hulled | 90 | 12.47 | 52.99 | 6.39 | 42.55 | 3.40 | 13.73 |
| B5094 | 2003 | Hulled | 94 | 16.03 | 53.24 | 5.86 | 40.67 | 3.32 | 10.70 |
| B5120 | 2003 | Hulled | 94 | 16.20 | 53.35 | 6.30 | 31.58 | 3.11 | 10.80 |
| B5703 | 2003 | Hulled | 98 | 16.20 | 53.56 | 6.19 | 30.47 | 3.07 | 11.16 |
| B4490 | 2003 | Hulled | 94 | 17.59 | 53.89 | 5.62 | 34.57 | 3.32 | 10.31 |
| B4544 | 2003 | Hulled | 70 | 13.63 | 53.90 | 7.70 | 53.51 | 3.73 | 13.39 |
| B5104 | 2003 | Hulled | 90 | 18.55 | 54.51 | 4.71 | 38.88 | 3.39 | 9.96 |
| B3973 | 2003 | Hulled | 86 | 14.91 | 54.75 | 4.90 | 34.59 | 3.45 | 8.74 |
| B18333 | 2003 | Hulled | 48 | 18.97 | 55.05 | 5.98 | 42.89 | 3.57 | 11.15 |
| B4019 | 2003 | Hulled | 84 | 15.98 | 55.36 | 6.39 | 47.37 | 3.59 | 12.39 |
| B4515 | 2003 | Hulled | 98 | 15.15 | 55.88 | 5.57 | 41.04 | 3.70 | 10.79 |
| B4559 | 2003 | Hulled | 98 | 13.53 | 55.88 | 6.97 | 50.56 | 3.68 | 12.30 |
| B4421 | 2003 | Hulled | 98 | 15.80 | 56.24 | 4.57 | 46.47 | 3.54 | 10.47 |
| B3698 | 2003 | Hulled | 90 | 18.46 | 56.31 | 4.41 | 34.08 | 3.35 | 10.08 |
| B3975 | 2003 | Hulled | 100 | 14.42 | 56.33 | 4.77 | 38.05 | 3.21 | 12.20 |
| B3959 | 2003 | Hulled | 90 | 16.60 | 56.33 | 4.25 | 41.35 | 3.42 | 10.71 |
| B3976 | 2003 | Hulled | 100 | 14.36 | 56.40 | 5.10 | 38.23 | 3.28 | 11.69 |

| | | | | | | | | | |
|---|---|---|---|---|---|---|---|---|---|
| B4383 | 2003 | Hulled | 36 | 16.87 | 56.58 | 4.63 | 54.62 | 3.84 | 11.24 |
| B4504 | 2003 | Hulled | 88 | 14.75 | 56.59 | 5.30 | 39.17 | 3.36 | 11.98 |
| B3966 | 2003 | Hulled | 90 | 15.88 | 56.70 | 5.57 | 35.35 | 3.20 | 11.59 |
| B4002 | 2003 | Hulled | 78 | 19.28 | 56.72 | 4.62 | 34.12 | 3.42 | 9.99 |
| B3828 | 2003 | Hulled | 74 | 13.74 | 56.82 | 5.06 | 39.26 | 3.25 | 11.61 |
| B3367 | 2003 | Hulled | 70 | 17.03 | 57.11 | 3.22 | 51.95 | 3.78 | 10.31 |
| B4639 | 2003 | Hulled | 98 | 13.61 | 57.11 | 5.88 | 42.25 | 3.61 | 12.34 |
| B3990 | 2003 | Hulled | 94 | 14.30 | 57.20 | 5.39 | 41.21 | 3.46 | 12.34 |
| B4510 | 2003 | Hulled | 94 | 15.85 | 57.20 | 4.35 | 42.49 | 3.70 | 9.95 |
| B4576 | 2003 | Hulled | 100 | 14.42 | 57.47 | 5.91 | 39.90 | 3.48 | 10.91 |
| B4573 | 2003 | Hulled | 96 | 14.27 | 57.60 | 5.83 | 36.07 | 3.23 | 11.49 |
| B4432 | 2003 | Hulled | 92 | 18.32 | 57.70 | 3.66 | 51.31 | 3.72 | 9.64 |
| B4502 | 2003 | Hulled | 74 | 14.79 | 57.70 | 5.48 | 38.14 | 3.35 | 11.06 |
| B4243 | 2003 | Hulled | 86 | 14.50 | 57.77 | 4.62 | 47.10 | 3.70 | 10.21 |
| B3692 | 2003 | Hulled | 100 | 12.99 | 57.90 | 5.41 | 35.98 | 3.23 | 11.96 |
| B4496 | 2003 | Hulled | 82 | 14.85 | 57.95 | 4.29 | 49.10 | 3.74 | 10.11 |
| B4673 | 2003 | Hulled | 94 | 13.55 | 58.01 | 5.07 | 47.65 | 3.57 | 13.38 |
| B4150 | 2003 | Hulled | 88 | 15.65 | 58.30 | 4.41 | 40.26 | 3.39 | 10.54 |
| B4431 | 2003 | Hulled | 96 | 15.23 | 58.34 | 4.77 | 44.17 | 3.54 | 11.93 |
| B4433 | 2003 | Hulled | 98 | 11.84 | 58.36 | 6.13 | 40.36 | 3.33 | 12.57 |
| B4561 | 2003 | Hulled | 94 | 14.71 | 58.72 | 5.04 | 41.08 | 3.32 | 10.67 |
| B4539 | 2003 | Hulled | 90 | 14.85 | 58.77 | 5.19 | 41.30 | 3.42 | 10.27 |
| B4119 | 2003 | Hulled | 96 | 13.68 | 58.86 | 4.97 | 37.69 | 3.42 | 11.79 |
| B4634 | 2003 | Hulled | 98 | 13.64 | 59.19 | 4.44 | 51.34 | 3.75 | 11.81 |
| B5173 | 2003 | Hulled | 98 | 11.80 | 59.21 | 4.87 | 35.68 | 3.09 | 11.86 |
| B4149 | 2003 | Hulled | 82 | 15.30 | 59.54 | 3.98 | 39.10 | 3.31 | 9.87 |
| B3999 | 2003 | Hulled | 96 | 14.98 | 60.02 | 3.48 | 51.44 | 3.65 | 9.81 |

| | | | | | | | | | |
|---|---|---|---|---|---|---|---|---|---|
| B4523 | 2003 | Hulled | 98 | 13.52 | 60.06 | 4.72 | 37.40 | 3.42 | 11.42 |
| B4636 | 2003 | Hulled | 96 | 12.82 | 60.70 | 4.55 | 49.48 | 3.74 | 11.25 |
| B4486 | 2003 | Hulled | 94 | 13.68 | 60.98 | 3.82 | 48.79 | 3.76 | 9.65 |
| B4695 | 2003 | Hulled | 96 | 13.46 | 60.99 | 4.00 | 46.60 | 3.68 | 9.84 |
| B3533 | 2003 | Hulled | 94 | 13.93 | 61.36 | 3.46 | 44.75 | 3.73 | 9.60 |
| B4687 | 2003 | Hulled | 86 | 14.59 | 61.52 | 3.70 | 41.19 | 3.53 | 9.32 |
| B4631 | 2003 | Hulled | 92 | 15.02 | 62.18 | 3.36 | 49.86 | 3.83 | 9.47 |
| B4524 | 2003 | Hulled | 94 | 12.95 | 62.53 | 3.93 | 44.78 | 3.74 | 9.25 |
| B4607 | 2004 | Hulled | 76 | 13.93 | 65.29 | 5.85 | 33.81 | 3.23 | 11.43 |
| B3650 | 2004 | Hulled | 96 | 12.34 | 52.78 | 7.08 | 43.63 | 3.55 | 13.65 |
| B4786 | 2004 | Hulled | 94 | 14.86 | 52.48 | 6.07 | 31.53 | 3.08 | 9.74 |
| B3651 | 2004 | Hulled | 94 | 12.27 | 55.01 | 6.65 | 51.09 | 3.75 | 12.70 |
| B4341 | 2004 | Hulled | 90 | 15.24 | 62.78 | 5.21 | 44.04 | 3.93 | 10.69 |
| B3462 | 2004 | Hulled | 90 | 12.41 | 54.36 | 6.56 | 37.90 | 3.42 | 12.50 |
| B5100 | 2004 | Hulled | 92 | 13.24 | 54.52 | 5.65 | 32.90 | 2.99 | 11.23 |
| B3418 | 2004 | Hulled | 74 | 12.85 | 55.91 | 5.96 | 43.87 | 3.67 | 10.77 |
| B4179 | 2004 | Hulled | 76 | 14.08 | 57.66 | 5.45 | 39.04 | 3.47 | 9.04 |
| B5115 | 2004 | Hulled | 100 | 14.39 | 56.53 | 4.62 | 35.20 | 3.26 | 10.36 |
| B5118 | 2004 | Hulled | 78 | 16.24 | 55.29 | 4.11 | 43.36 | 3.46 | 9.13 |
| B5098 | 2004 | Hulled | 96 | 15.84 | 54.94 | 5.04 | 22.16 | 2.78 | 9.70 |
| B5088 | 2004 | Hulled | 94 | 15.24 | 55.51 | 5.25 | 24.60 | 2.96 | 9.37 |
| B3415 | 2004 | Hulled | 90 | 12.16 | 55.26 | 6.29 | 44.39 | 3.62 | 10.97 |
| B5114 | 2004 | Hulled | 94 | 14.74 | 56.39 | 4.64 | 31.39 | 3.05 | 11.57 |
| B4506 | 2004 | Hulled | 96 | 14.04 | 56.76 | 5.26 | 29.19 | 3.07 | 10.35 |
| B4564 | 2004 | Hulled | 90 | 17.44 | 55.83 | 5.57 | 39.77 | 3.55 | 11.59 |
| B5101 | 2004 | Hulled | 94 | 14.05 | 54.65 | 5.27 | 31.36 | 2.99 | 9.68 |
| B3608 | 2004 | Hulled | 96 | 14.18 | 56.73 | 4.66 | 38.26 | 3.26 | 11.75 |

| | | | | | | | | | |
|---|---|---|---|---|---|---|---|---|---|
| B3706 | 2004 | Hulled | 100 | 12.73 | 59.63 | 4.66 | 31.24 | 3.36 | 8.83 |
| B3712 | 2004 | Hulled | 100 | 13.35 | 58.80 | 4.04 | 26.44 | 3.04 | 7.74 |
| B3457 | 2004 | Hulled | 84 | 14.09 | 57.93 | 5.39 | 46.22 | 3.78 | 10.08 |
| B3601 | 2004 | Hulled | 90 | 12.64 | 59.90 | 4.35 | 36.72 | 3.47 | 8.30 |
| B3672 | 2004 | Hulled | 100 | 11.30 | 56.89 | 6.11 | 42.92 | 3.47 | 11.70 |
| B3700 | 2004 | Hulled | 88 | 13.68 | 58.96 | 4.25 | 31.44 | 3.34 | 7.75 |
| B3991 | 2004 | Hulled | 98 | 11.92 | 59.69 | 5.28 | 33.95 | 3.29 | 11.90 |
| B3468 | 2004 | Hulled | 90 | 16.49 | 58.82 | 3.42 | 39.48 | 3.50 | 8.02 |
| B3529 | 2004 | Hulled | 90 | 13.59 | 58.09 | 4.88 | 36.51 | 3.21 | 10.28 |
| B3653 | 2004 | Hulled | 92 | 13.38 | 55.81 | 5.50 | 40.63 | 3.59 | 11.36 |
| B3691 | 2004 | Hulled | 54 | 12.66 | 59.54 | 5.39 | 36.73 | 3.35 | 9.34 |
| B3384 | 2004 | Hulled | 96 | 14.95 | 57.42 | 4.15 | 37.62 | 3.44 | 10.22 |
| B3570 | 2004 | Hulled | 98 | 11.42 | 58.77 | 5.71 | 47.29 | 3.45 | 11.94 |
| B3645 | 2004 | Hulled | 88 | 11.73 | 55.56 | 6.33 | 48.13 | 3.60 | 12.14 |
| B3646 | 2004 | Hulled | 100 | 11.32 | 55.56 | 6.54 | 47.06 | 3.55 | 12.62 |
| B3656 | 2004 | Hulled | 88 | 12.67 | 56.70 | 5.92 | 50.00 | 3.83 | 10.62 |
| B3674 | 2004 | Hulled | 98 | 12.56 | 56.28 | 6.13 | 42.74 | 3.49 | 10.43 |
| B4572 | 2004 | Hulled | 98 | 15.89 | 57.94 | 4.88 | 32.87 | 3.17 | 10.44 |
| B4583 | 2004 | Hulled | 98 | 13.91 | 60.75 | 3.74 | 33.63 | 3.23 | 9.55 |
| B3473 | 2004 | Hulled | 90 | 13.31 | 61.33 | 3.53 | 44.87 | 3.87 | 9.25 |
| B3635 | 2004 | Hulled | 58 | 12.06 | 58.73 | 5.51 | 37.74 | 3.31 | 9.80 |
| B4488 | 2004 | Hulled | 100 | 12.79 | 60.29 | 4.47 | 34.90 | 3.32 | 9.99 |
| B3359 | 2004 | Hulled | 96 | 11.76 | 64.20 | 3.75 | 38.48 | 3.45 | 8.93 |
| B3528 | 2004 | Hulled | 96 | 12.38 | 60.87 | 3.95 | 45.85 | 3.72 | 9.96 |
| B3725 | 2004 | Hulled | 96 | 13.01 | 62.02 | 3.23 | 40.66 | 3.53 | 9.42 |
| B3727 | 2004 | Hulled | 98 | 12.59 | 57.02 | 5.93 | 44.59 | 3.61 | 11.03 |
| B3957 | 2004 | Hulled | 98 | 13.84 | 62.12 | 3.12 | 41.57 | 3.70 | 9.49 |

| | | | | | | | | | |
|---|---|---|---|---|---|---|---|---|---|
| B4355 | 2004 | Hulled | 92 | 14.67 | 62.54 | 3.23 | 26.40 | 3.11 | 8.03 |
| B5093 | 2004 | Hulled | 100 | 14.67 | 55.25 | 5.41 | 26.61 | 2.83 | 10.15 |
| B5097 | 2004 | Hulled | 98 | 16.23 | 56.61 | 4.68 | 22.62 | 2.96 | 7.39 |
| B3980 | 2004 | Hulled | 94 | 13.32 | 57.65 | 4.89 | 37.92 | 3.48 | 10.09 |
| B4481 | 2004 | Hulled | 84 | 12.80 | 59.00 | 4.79 | 33.86 | 3.21 | 12.08 |
| B4522 | 2004 | Hulled | 96 | 13.74 | 61.29 | 3.95 | 32.55 | 3.26 | 11.08 |
| B3346 | 2004 | Hulled | 96 | 12.81 | 60.10 | 4.27 | 44.14 | 3.48 | 10.10 |
| B3401 | 2004 | Hulled | 100 | 13.02 | 61.25 | 3.85 | 44.96 | 3.73 | 8.42 |
| B3431 | 2004 | Hulled | 94 | 12.81 | 62.50 | 3.13 | 39.86 | 3.55 | 8.99 |
| B5092 | 2004 | Hulled | 100 | 15.94 | 54.17 | 5.42 | 25.21 | 2.73 | 9.80 |
| B5105 | 2004 | Hulled | 98 | 14.90 | 55.94 | 4.79 | 32.76 | 3.11 | 8.97 |
| B5106 | 2004 | Hulled | 98 | 14.48 | 55.73 | 4.90 | 33.73 | 3.08 | 9.89 |
| B3664 | 2004 | Hulled | 98 | 12.81 | 62.29 | 3.75 | 32.56 | 3.21 | 10.42 |
| B3658 | 2004 | Hulled | 94 | 11.57 | 58.29 | 6.15 | 48.78 | 3.54 | 11.84 |
| B4418 | 2004 | Hulled | 96 | 13.03 | 59.44 | 4.38 | 32.82 | 3.21 | 9.49 |
| B4517 | 2004 | Hulled | 86 | 13.14 | 60.17 | 4.59 | 37.19 | 3.48 | 10.06 |
| B4567 | 2004 | Hulled | 96 | 15.85 | 59.12 | 4.59 | 38.82 | 3.52 | 10.48 |
| B3662 | 2004 | Hulled | 98 | 12.20 | 62.25 | 3.55 | 31.84 | 3.06 | 9.09 |
| B3665 | 2004 | Hulled | 100 | 12.72 | 60.06 | 4.17 | 32.37 | 3.15 | 10.23 |
| B4007 | 2004 | Hulled | 92 | 14.39 | 59.12 | 4.48 | 31.01 | 3.33 | 7.64 |
| B3595 | 2004 | Hulled | 92 | 13.78 | 60.44 | 3.55 | 47.11 | 3.75 | 9.13 |
| B3648 | 2004 | Hulled | 92 | 11.48 | 59.60 | 5.74 | 43.28 | 3.60 | 11.41 |
| B3655 | 2004 | Hulled | 90 | 14.20 | 57.93 | 5.53 | 42.89 | 3.33 | 11.91 |
| B4240 | 2004 | Hulled | 98 | 13.99 | 58.25 | 5.01 | 32.94 | 3.21 | 11.11 |
| B4512 | 2004 | Hulled | 100 | 13.15 | 60.02 | 4.38 | 30.42 | 3.20 | 8.87 |
| B3474 | 2004 | Hulled | 98 | 14.00 | 60.19 | 3.66 | 33.39 | 3.29 | 8.32 |
| B3545 | 2004 | Hulled | 92 | 12.43 | 61.96 | 4.08 | 32.45 | 3.20 | 8.61 |

| | | | | | | | | | |
|---|---|---|---|---|---|---|---|---|---|
| B3593 | 2004 | Hulled | 94 | 12.85 | 60.82 | 3.45 | 46.85 | 3.68 | 8.78 |
| B3657 | 2004 | Hulled | 94 | 11.70 | 57.58 | 5.96 | 42.07 | 3.51 | 11.28 |
| B4159 | 2004 | Hulled | 98 | 11.81 | 58.83 | 5.33 | 42.41 | 3.45 | 11.52 |
| B4493 | 2004 | Hulled | 100 | 11.70 | 61.13 | 5.22 | 35.15 | 3.40 | 10.06 |
| B4521 | 2004 | Hulled | 92 | 13.27 | 60.92 | 4.08 | 37.00 | 3.40 | 9.47 |
| B3714 | 2004 | Hulled | 100 | 12.64 | 61.86 | 3.87 | 34.49 | 3.27 | 8.05 |
| B4001 | 2004 | Hulled | 100 | 12.96 | 61.02 | 3.66 | 31.59 | 3.27 | 8.56 |
| B4115 | 2004 | Hulled | 96 | 12.64 | 61.34 | 4.60 | 38.64 | 3.43 | 10.15 |
| B3442 | 2004 | Hulled | 72 | 13.28 | 61.51 | 3.66 | 46.10 | 3.67 | 8.88 |
| B3498 | 2004 | Hulled | 100 | 16.00 | 59.31 | 3.35 | 42.62 | 3.73 | 9.98 |
| B4501 | 2004 | Hulled | 76 | 15.90 | 61.19 | 3.14 | 46.12 | 3.77 | 9.04 |
| B4514 | 2004 | Hulled | 98 | 12.76 | 61.72 | 4.18 | 31.16 | 3.25 | 9.13 |
| B5108 | 2004 | Hulled | 100 | 13.91 | 58.37 | 4.18 | 33.40 | 3.18 | 9.85 |
| B3668 | 2004 | Hulled | 94 | 13.49 | 59.62 | 3.66 | 31.66 | 3.25 | 10.10 |
| B3702 | 2004 | Hulled | 94 | 13.60 | 59.83 | 4.18 | 33.45 | 3.39 | 7.06 |
| B3434 | 2004 | Hulled | 84 | 14.14 | 61.05 | 3.87 | 46.98 | 3.83 | 8.19 |
| B3447 | 2004 | Hulled | 100 | 12.77 | 62.41 | 3.14 | 37.11 | 3.36 | 8.40 |
| B3659 | 2004 | Hulled | 84 | 12.36 | 57.07 | 5.97 | 52.35 | 3.75 | 10.82 |
| B4161 | 2004 | Hulled | 100 | 13.19 | 59.79 | 4.71 | 40.42 | 3.35 | 11.28 |
| B4518 | 2004 | Hulled | 88 | 12.88 | 61.36 | 4.29 | 32.39 | 3.32 | 9.21 |
| B3984 | 2004 | Hulled | 98 | 11.73 | 61.99 | 4.40 | 33.63 | 3.19 | 10.88 |
| B3402 | 2004 | Hulled | 100 | 12.16 | 61.32 | 4.09 | 40.45 | 3.56 | 8.56 |
| B3405 | 2004 | Hulled | 100 | 12.79 | 61.32 | 3.77 | 45.11 | 3.70 | 8.81 |
| B3521 | 2004 | Hulled | 98 | 11.01 | 65.62 | 3.04 | 34.70 | 3.32 | 7.82 |
| B3603 | 2004 | Hulled | 94 | 11.11 | 58.70 | 5.45 | 43.30 | 3.63 | 12.42 |
| B4453 | 2004 | Hulled | 96 | 13.84 | 60.48 | 4.19 | 48.24 | 3.57 | 11.26 |
| B4520 | 2004 | Hulled | 100 | 12.37 | 59.22 | 5.14 | 31.81 | 3.25 | 9.59 |

| | | | | | | | | | |
|---|---|---|---|---|---|---|---|---|---|
| B4585 | 2004 | Hulled | 94 | 14.36 | 60.27 | 4.40 | 50.10 | 3.73 | 10.91 |
| B3364 | 2004 | Hulled | 68 | 16.14 | 59.85 | 3.46 | 55.91 | 3.98 | 10.38 |
| B3989 | 2004 | Hulled | 100 | 13.00 | 60.90 | 4.30 | 37.89 | 3.45 | 11.68 |
| B3397 | 2004 | Hulled | 100 | 12.59 | 62.43 | 3.57 | 40.23 | 3.64 | 9.19 |
| B3619 | 2004 | Hulled | 98 | 10.60 | 63.69 | 3.67 | 43.27 | 3.63 | 9.26 |
| B3670 | 2004 | Hulled | 100 | 11.65 | 62.22 | 4.09 | 35.61 | 3.27 | 10.05 |
| B3992 | 2004 | Hulled | 96 | 12.17 | 60.23 | 5.04 | 35.86 | 3.22 | 10.22 |
| B4114 | 2004 | Hulled | 94 | 12.49 | 62.01 | 4.51 | 42.57 | 3.61 | 10.17 |
| B4177 | 2004 | Hulled | 94 | 12.17 | 62.01 | 3.67 | 44.80 | 3.70 | 9.81 |
| B3694 | 2004 | Hulled | 88 | 13.54 | 58.55 | 4.62 | 30.39 | 3.31 | 7.85 |
| B4156 | 2004 | Hulled | 90 | 13.54 | 59.60 | 4.30 | 44.79 | 3.64 | 11.08 |
| B3605 | 2004 | Hulled | 92 | 13.55 | 62.71 | 3.26 | 41.75 | 3.55 | 9.12 |
| B3630 | 2004 | Hulled | 92 | 13.24 | 62.92 | 3.47 | 42.91 | 3.63 | 8.68 |
| B3679 | 2004 | Hulled | 100 | 12.29 | 61.97 | 4.31 | 37.20 | 3.34 | 10.18 |
| B3689 | 2004 | Hulled | 90 | 11.87 | 64.39 | 3.15 | 43.22 | 3.62 | 8.38 |
| B3730 | 2004 | Hulled | 90 | 12.61 | 63.34 | 3.15 | 39.67 | 3.67 | 7.70 |
| B4519 | 2004 | Hulled | 92 | 12.71 | 60.40 | 4.52 | 31.92 | 3.33 | 9.11 |
| B4677 | 2004 | Hulled | 94 | 12.29 | 62.92 | 3.68 | 39.64 | 3.44 | 8.44 |
| B4694 | 2004 | Hulled | 98 | 12.61 | 62.50 | 3.36 | 39.88 | 3.58 | 8.40 |
| B3465 | 2004 | Hulled | 94 | 14.41 | 62.25 | 3.36 | 41.43 | 3.66 | 7.80 |
| B3472 | 2004 | Hulled | 90 | 15.14 | 60.46 | 3.47 | 46.53 | 3.88 | 9.31 |
| B3525 | 2004 | Hulled | 94 | 12.09 | 62.88 | 4.10 | 38.80 | 3.53 | 9.47 |
| B3588 | 2004 | Hulled | 78 | 12.51 | 62.67 | 3.47 | 47.66 | 3.74 | 8.29 |
| B3628 | 2004 | Hulled | 94 | 12.62 | 63.72 | 3.36 | 38.05 | 3.53 | 8.22 |
| B3683 | 2004 | Hulled | 100 | 12.20 | 62.46 | 3.68 | 40.59 | 3.49 | 8.41 |
| B3690 | 2004 | Hulled | 92 | 13.25 | 60.99 | 4.00 | 32.29 | 3.26 | 8.62 |
| B3726 | 2004 | Hulled | 90 | 12.41 | 64.25 | 3.15 | 40.17 | 3.46 | 8.50 |

| | | | | | | | | | |
|---|---|---|---|---|---|---|---|---|---|
| B3963 | 2004 | Hulled | 98 | 11.67 | 64.67 | 3.47 | 42.27 | 3.63 | 8.75 |
| B4419 | 2004 | Hulled | 78 | 14.83 | 61.09 | 3.79 | 37.11 | 3.38 | 8.81 |
| B4509 | 2004 | Hulled | 84 | 12.62 | 63.09 | 4.10 | 34.18 | 3.41 | 8.59 |
| B4511 | 2004 | Hulled | 92 | 13.99 | 61.51 | 4.31 | 32.02 | 3.33 | 7.77 |
| B4568 | 2004 | Hulled | 94 | 14.62 | 60.88 | 4.52 | 38.06 | 3.43 | 9.53 |
| B4580 | 2004 | Hulled | 98 | 15.46 | 60.15 | 3.36 | 43.18 | 3.58 | 8.16 |
| B5110 | 2004 | Hulled | 94 | 13.88 | 58.89 | 4.42 | 33.60 | 3.15 | 9.38 |
| B3411 | 2004 | Hulled | 92 | 13.16 | 62.53 | 3.58 | 48.39 | 3.87 | 8.51 |
| B3466 | 2004 | Hulled | 98 | 12.21 | 63.16 | 3.37 | 35.12 | 3.47 | 9.08 |
| B3508 | 2004 | Hulled | 94 | 12.95 | 63.89 | 3.26 | 41.98 | 3.65 | 7.87 |
| B3516 | 2004 | Hulled | 98 | 14.32 | 62.74 | 3.16 | 43.49 | 3.69 | 7.88 |
| B3581 | 2004 | Hulled | 94 | 13.05 | 63.26 | 2.84 | 43.35 | 3.60 | 8.64 |
| B3610 | 2004 | Hulled | 98 | 12.63 | 64.21 | 3.16 | 40.09 | 3.52 | 8.69 |
| B3624 | 2004 | Hulled | 94 | 12.11 | 64.84 | 3.05 | 41.66 | 3.65 | 8.84 |
| B4387 | 2004 | Hulled | 92 | 14.21 | 62.11 | 3.37 | 43.19 | 3.70 | 8.46 |
| B4391 | 2004 | Hulled | 94 | 12.11 | 62.00 | 4.21 | 57.79 | 3.83 | 10.26 |
| B4447 | 2004 | Hulled | 88 | 14.84 | 62.63 | 3.26 | 33.51 | 3.51 | 6.74 |
| B4508 | 2004 | Hulled | 96 | 14.21 | 62.74 | 3.26 | 33.24 | 3.27 | 7.97 |
| B4513 | 2004 | Hulled | 100 | 13.05 | 62.11 | 4.21 | 31.87 | 3.40 | 7.97 |
| B4606 | 2004 | Hulled | 90 | 14.32 | 59.26 | 4.95 | 31.89 | 2.99 | 10.39 |
| B4645 | 2004 | Hulled | 90 | 13.68 | 61.26 | 3.47 | 45.02 | 3.53 | 8.87 |
| B4646 | 2004 | Hulled | 76 | 15.37 | 61.05 | 3.05 | 49.18 | 3.78 | 8.34 |
| B4688 | 2004 | Hulled | 92 | 16.63 | 61.79 | 2.84 | 37.02 | 3.37 | 8.26 |
| B4693 | 2004 | Hulled | 74 | 14.32 | 60.32 | 3.79 | 47.48 | 3.74 | 9.42 |
| B5107 | 2004 | Hulled | 90 | 14.53 | 58.95 | 4.00 | 34.22 | 3.13 | 8.34 |
| B3663 | 2004 | Hulled | 100 | 12.42 | 62.95 | 3.47 | 32.32 | 3.22 | 8.33 |
| B4647 | 2004 | Hulled | 92 | 13.37 | 62.42 | 3.79 | 44.83 | 3.68 | 7.96 |

| | | | | | | | | | |
|---|---|---|---|---|---|---|---|---|---|
| B3382 | 2004 | Hulled | 72 | 12.75 | 63.33 | 3.48 | 44.16 | 3.54 | 8.97 |
| B3400 | 2004 | Hulled | 86 | 14.65 | 61.96 | 3.27 | 41.18 | 3.55 | 8.70 |
| B3407 | 2004 | Hulled | 94 | 11.91 | 61.75 | 4.00 | 48.95 | 3.78 | 8.83 |
| B3470 | 2004 | Hulled | 100 | 13.07 | 64.91 | 2.85 | 39.02 | 3.59 | 8.23 |
| B3480 | 2004 | Hulled | 88 | 14.44 | 64.07 | 2.95 | 39.29 | 3.53 | 7.72 |
| B3481 | 2004 | Hulled | 96 | 12.86 | 63.75 | 3.27 | 46.13 | 3.69 | 8.00 |
| B3590 | 2004 | Hulled | 90 | 14.54 | 62.28 | 3.37 | 36.52 | 3.32 | 8.66 |
| B3597 | 2004 | Hulled | 92 | 14.23 | 61.75 | 3.16 | 43.85 | 3.65 | 8.58 |
| B3625 | 2004 | Hulled | 92 | 12.86 | 65.02 | 2.63 | 38.41 | 3.50 | 7.75 |
| B3643 | 2004 | Hulled | 94 | 13.59 | 62.70 | 3.48 | 29.01 | 3.23 | 7.28 |
| B3718 | 2004 | Hulled | 96 | 11.59 | 64.91 | 3.48 | 42.51 | 3.68 | 8.87 |
| B4255 | 2004 | Hulled | 84 | 11.80 | 62.91 | 4.11 | 29.58 | 3.04 | 10.01 |
| B4382 | 2004 | Hulled | 100 | 11.38 | 62.91 | 3.90 | 37.90 | 3.51 | 7.64 |
| B4445 | 2004 | Hulled | 98 | 12.75 | 58.38 | 5.58 | 51.06 | 3.53 | 11.67 |
| B4449 | 2004 | Hulled | 94 | 11.91 | 59.64 | 4.64 | 40.46 | 3.38 | 10.65 |
| B4632 | 2004 | Hulled | 90 | 13.49 | 62.91 | 3.48 | 38.46 | 3.48 | 7.94 |
| B5113 | 2004 | Hulled | 78 | 13.80 | 58.38 | 4.21 | 30.96 | 3.04 | 8.17 |
| B3389 | 2004 | Hulled | 78 | 13.40 | 64.03 | 3.27 | 46.95 | 3.81 | 7.62 |
| B3412 | 2004 | Hulled | 86 | 12.45 | 63.29 | 3.59 | 44.00 | 3.74 | 8.15 |
| B3427 | 2004 | Hulled | 100 | 12.34 | 64.45 | 3.06 | 40.79 | 3.52 | 8.80 |
| B3461 | 2004 | Hulled | 92 | 13.61 | 61.92 | 3.80 | 42.13 | 3.58 | 8.30 |
| B3638 | 2004 | Hulled | 94 | 12.66 | 62.55 | 4.11 | 46.26 | 3.77 | 8.03 |
| B4392 | 2004 | Hulled | 96 | 11.18 | 63.19 | 4.01 | 35.15 | 3.22 | 10.49 |
| B4401 | 2004 | Hulled | 98 | 11.92 | 63.50 | 3.69 | 39.91 | 3.55 | 8.72 |
| B4455 | 2004 | Hulled | 100 | 10.97 | 59.70 | 5.27 | 39.33 | 3.27 | 10.82 |
| B4498 | 2004 | Hulled | 84 | 14.14 | 61.60 | 3.90 | 46.60 | 3.73 | 8.73 |
| B4516 | 2004 | Hulled | 96 | 12.45 | 63.29 | 3.69 | 36.04 | 3.48 | 8.29 |

| | | | | | | | | | |
|---|---|---|---|---|---|---|---|---|---|
| B4578 | 2004 | Hulled | 90 | 14.77 | 61.71 | 3.16 | 40.28 | 3.53 | 8.13 |
| B4611 | 2004 | Hulled | 86 | 13.29 | 63.71 | 3.27 | 49.01 | 3.88 | 8.60 |
| B4623 | 2004 | Hulled | 98 | 13.08 | 64.87 | 3.59 | 43.72 | 3.70 | 9.41 |
| B4649 | 2004 | Hulled | 72 | 15.08 | 64.35 | 2.32 | 42.52 | 3.74 | 7.42 |
| B3409 | 2004 | Hulled | 94 | 14.78 | 61.03 | 3.80 | 46.07 | 3.76 | 8.14 |
| B3429 | 2004 | Hulled | 84 | 13.52 | 64.94 | 2.75 | 37.85 | 3.48 | 8.20 |
| B3455 | 2004 | Hulled | 72 | 14.26 | 63.67 | 3.27 | 41.35 | 3.59 | 7.61 |
| B3499 | 2004 | Hulled | 90 | 12.35 | 64.31 | 3.17 | 46.04 | 3.76 | 8.62 |
| B3616 | 2004 | Hulled | 96 | 13.31 | 63.46 | 3.38 | 38.37 | 3.50 | 8.49 |
| B3622 | 2004 | Hulled | 96 | 12.14 | 64.20 | 2.96 | 38.38 | 3.52 | 8.23 |
| B3686 | 2004 | Hulled | 98 | 12.46 | 65.47 | 2.75 | 43.11 | 3.58 | 8.11 |
| B3721 | 2004 | Hulled | 94 | 12.67 | 64.84 | 2.64 | 40.41 | 3.50 | 7.87 |
| B3747 | 2004 | Hulled | 94 | 13.94 | 61.99 | 3.38 | 45.54 | 3.66 | 7.77 |
| B4174 | 2004 | Hulled | 98 | 12.04 | 64.10 | 3.80 | 47.24 | 3.72 | 9.87 |
| B4228 | 2004 | Hulled | 100 | 13.73 | 60.51 | 4.01 | 46.24 | 3.72 | 9.12 |
| B4426 | 2004 | Hulled | 84 | 11.72 | 61.77 | 3.91 | 44.47 | 3.36 | 11.47 |
| B4581 | 2004 | Hulled | 92 | 13.09 | 63.36 | 3.06 | 41.27 | 3.50 | 8.45 |
| B4640 | 2004 | Hulled | 94 | 12.67 | 64.63 | 3.06 | 37.99 | 3.32 | 7.75 |
| B3579 | 2004 | Hulled | 98 | 12.57 | 64.52 | 3.17 | 37.78 | 3.48 | 8.03 |
| B3464 | 2004 | Hulled | 92 | 13.85 | 64.90 | 3.07 | 44.07 | 3.68 | 8.26 |
| B3479 | 2004 | Hulled | 90 | 12.68 | 64.38 | 3.59 | 40.33 | 3.67 | 7.63 |
| B3626 | 2004 | Hulled | 92 | 13.21 | 65.33 | 2.96 | 40.16 | 3.60 | 7.94 |
| B3958 | 2004 | Hulled | 100 | 12.79 | 64.90 | 2.85 | 41.13 | 3.52 | 8.70 |
| B4000 | 2004 | Hulled | 78 | 13.21 | 62.16 | 4.12 | 34.01 | 3.20 | 10.01 |
| B4209 | 2004 | Hulled | 100 | 12.37 | 63.53 | 3.38 | 36.37 | 3.46 | 8.23 |
| B4463 | 2004 | Hulled | 98 | 12.58 | 60.89 | 4.44 | 36.34 | 3.20 | 10.02 |
| B4653 | 2004 | Hulled | 100 | 13.53 | 63.11 | 3.28 | 43.08 | 3.69 | 7.63 |

| | | | | | | | | | |
|---|---|---|---|---|---|---|---|---|---|
| B4008 | 2004 | Hulled | 94 | 12.68 | 63.64 | 3.59 | 28.98 | 3.17 | 8.35 |
| B3406 | 2004 | Hulled | 76 | 13.76 | 63.17 | 3.70 | 46.84 | 3.75 | 7.97 |
| B3449 | 2004 | Hulled | 100 | 13.65 | 64.87 | 2.86 | 41.25 | 3.56 | 7.55 |
| B3476 | 2004 | Hulled | 98 | 13.12 | 64.66 | 3.07 | 37.85 | 3.41 | 8.64 |
| B3506 | 2004 | Hulled | 92 | 13.65 | 63.39 | 3.07 | 41.63 | 3.50 | 9.05 |
| B3612 | 2004 | Hulled | 82 | 14.60 | 63.92 | 2.75 | 44.61 | 3.73 | 7.67 |
| B3685 | 2004 | Hulled | 92 | 11.43 | 64.97 | 3.39 | 39.64 | 3.62 | 7.62 |
| B3722 | 2004 | Hulled | 82 | 12.80 | 64.76 | 3.39 | 44.02 | 3.57 | 8.25 |
| B3732 | 2004 | Hulled | 98 | 12.59 | 64.87 | 2.75 | 42.33 | 3.68 | 9.27 |
| B4160 | 2004 | Hulled | 94 | 14.60 | 61.69 | 3.81 | 49.15 | 3.80 | 9.11 |
| B4423 | 2004 | Hulled | 92 | 12.59 | 60.53 | 4.55 | 37.49 | 3.47 | 7.76 |
| B4505 | 2004 | Hulled | 94 | 13.97 | 64.44 | 2.75 | 45.62 | 3.69 | 8.31 |
| B4525 | 2004 | Hulled | 92 | 13.33 | 63.07 | 3.49 | 37.64 | 3.54 | 8.62 |
| B4528 | 2004 | Hulled | 76 | 15.24 | 61.90 | 3.49 | 47.28 | 3.66 | 8.39 |
| B4579 | 2004 | Hulled | 92 | 14.18 | 62.65 | 3.17 | 43.10 | 3.56 | 8.29 |
| B4633 | 2004 | Hulled | 80 | 11.85 | 65.29 | 3.17 | 40.54 | 3.55 | 8.61 |
| B4648 | 2004 | Hulled | 78 | 13.23 | 64.55 | 3.17 | 44.59 | 3.64 | 8.06 |
| B4690 | 2004 | Hulled | 98 | 11.64 | 65.61 | 3.39 | 34.82 | 3.40 | 8.28 |
| B3460 | 2004 | Hulled | 90 | 13.03 | 66.00 | 2.65 | 40.25 | 3.57 | 7.67 |
| B4018 | 2004 | Hulled | 96 | 11.65 | 62.18 | 4.98 | 48.94 | 3.69 | 10.73 |
| B4148 | 2004 | Hulled | 100 | 11.55 | 64.30 | 3.39 | 42.05 | 3.62 | 8.80 |
| B4176 | 2004 | Hulled | 94 | 12.82 | 63.03 | 3.50 | 47.80 | 3.69 | 8.76 |
| B4215 | 2004 | Hulled | 94 | 12.92 | 64.19 | 3.39 | 42.05 | 3.63 | 7.69 |
| B4414 | 2004 | Hulled | 100 | 11.76 | 66.00 | 2.97 | 37.22 | 3.44 | 7.80 |
| B4422 | 2004 | Hulled | 96 | 12.82 | 63.45 | 3.50 | 42.82 | 3.63 | 8.82 |
| B4450 | 2004 | Hulled | 94 | 13.24 | 62.82 | 3.60 | 42.73 | 3.39 | 8.74 |
| B4456 | 2004 | Hulled | 94 | 14.51 | 61.02 | 3.81 | 41.52 | 3.57 | 7.93 |

| | | | | | | | | | | |
|---|---|---|---|---|---|---|---|---|---|---|
| B4610 | 2004 | Hulled | 64 | 13.24 | 61.55 | 3.92 | 34.42 | 3.13 | 10.15 |
| B4531 | 2004 | Hulled | 70 | 14.41 | 61.02 | 3.92 | 45.31 | 3.69 | 10.01 |
| B3345 | 2004 | Hulled | 94 | 14.32 | 63.41 | 2.97 | 40.34 | 3.53 | 7.57 |
| B3494 | 2004 | Hulled | 96 | 12.51 | 63.73 | 3.71 | 32.01 | 3.21 | 7.67 |
| B3618 | 2004 | Hulled | 94 | 14.00 | 62.57 | 3.61 | 31.32 | 3.11 | 7.54 |
| B4461 | 2004 | Hulled | 92 | 13.47 | 63.10 | 3.50 | 49.38 | 3.78 | 8.44 |
| B4563 | 2004 | Hulled | 98 | 14.53 | 60.55 | 4.14 | 44.95 | 3.56 | 10.73 |
| B4582 | 2004 | Hulled | 100 | 13.57 | 63.73 | 3.08 | 44.57 | 3.55 | 8.25 |
| B4599 | 2004 | Hulled | 98 | 12.09 | 67.23 | 2.23 | 37.85 | 3.54 | 9.03 |
| B3611 | 2004 | Hulled | 74 | 14.01 | 65.07 | 3.18 | 41.66 | 3.59 | 7.62 |
| B4151 | 2004 | Hulled | 90 | 16.99 | 63.06 | 2.76 | 44.51 | 3.76 | 7.59 |
| B4420 | 2004 | Hulled | 94 | 13.91 | 61.68 | 3.72 | 47.77 | 3.62 | 8.54 |
| B4499 | 2004 | Hulled | 100 | 13.06 | 63.27 | 4.14 | 42.55 | 3.68 | 7.57 |
| B4532 | 2004 | Hulled | 96 | 13.48 | 64.12 | 3.18 | 36.81 | 3.47 | 8.12 |
| B4598 | 2004 | Hulled | 100 | 12.63 | 66.35 | 2.76 | 40.44 | 3.56 | 7.97 |
| B3477 | 2004 | Hulled | 84 | 13.28 | 65.89 | 2.87 | 40.82 | 3.60 | 7.12 |
| B3680 | 2004 | Hulled | 72 | 11.90 | 66.84 | 3.08 | 40.25 | 3.37 | 9.05 |
| B3746 | 2004 | Hulled | 98 | 14.24 | 64.08 | 2.76 | 44.98 | 3.68 | 7.46 |
| B4210 | 2004 | Hulled | 98 | 12.86 | 65.46 | 2.98 | 34.17 | 3.39 | 7.82 |
| B4211 | 2004 | Hulled | 100 | 12.33 | 62.06 | 4.36 | 36.86 | 3.42 | 8.55 |
| B4600 | 2004 | Hulled | 100 | 13.82 | 63.12 | 3.93 | 48.63 | 3.80 | 8.74 |
| B4624 | 2004 | Hulled | 90 | 12.96 | 65.78 | 3.61 | 38.88 | 3.48 | 7.65 |
| B3965 | 2004 | Hulled | 88 | 12.98 | 62.13 | 4.36 | 38.43 | 3.31 | 8.65 |
| B4424 | 2004 | Hulled | 90 | 14.89 | 63.51 | 2.98 | 41.68 | 3.43 | 8.63 |
| B4494 | 2004 | Hulled | 98 | 12.98 | 62.23 | 3.51 | 45.70 | 3.64 | 9.80 |
| B3419 | 2004 | Hulled | 68 | 14.70 | 65.28 | 2.98 | 39.44 | 3.51 | 7.21 |
| B3488 | 2004 | Hulled | 96 | 13.21 | 64.96 | 3.19 | 36.14 | 3.48 | 7.10 |

| | | | | | | | | | |
|---|---|---|---|---|---|---|---|---|---|
| B4207 | 2004 | Hulled | 100 | 12.46 | 64.22 | 3.51 | 40.63 | 3.66 | 7.97 |
| B4602 | 2004 | Hulled | 100 | 13.42 | 64.32 | 3.51 | 39.31 | 3.67 | 7.31 |
| B3483 | 2004 | Hulled | 90 | 11.30 | 66.84 | 3.20 | 42.68 | 3.45 | 8.05 |
| B3636 | 2004 | Hulled | 84 | 14.39 | 65.88 | 2.77 | 39.38 | 3.55 | 7.34 |
| B3997 | 2004 | Hulled | 96 | 11.62 | 65.46 | 3.73 | 38.73 | 3.33 | 8.96 |
| B4271 | 2004 | Hulled | 96 | 14.39 | 64.07 | 3.09 | 43.41 | 3.51 | 8.41 |
| B4558 | 2004 | Hulled | 100 | 12.15 | 65.67 | 3.52 | 41.27 | 3.54 | 9.28 |
| B4601 | 2004 | Hulled | 96 | 12.47 | 65.88 | 3.20 | 38.29 | 3.59 | 7.17 |
| B4553 | 2004 | Hulled | 80 | 13.23 | 63.61 | 3.52 | 44.93 | 3.58 | 9.64 |
| B4213 | 2004 | Hulled | 98 | 12.39 | 65.81 | 3.42 | 37.09 | 3.54 | 7.49 |
| B4248 | 2004 | Hulled | 96 | 14.64 | 62.39 | 3.95 | 21.54 | 2.98 | 7.36 |
| B4399 | 2004 | Hulled | 92 | 14.64 | 65.81 | 2.67 | 43.05 | 3.66 | 8.29 |
| B4541 | 2004 | Hulled | 84 | 12.82 | 66.56 | 2.99 | 42.69 | 3.59 | 8.14 |
| B4625 | 2004 | Hulled | 100 | 13.26 | 66.10 | 3.32 | 36.79 | 3.51 | 7.57 |
| B3519 | 2004 | Hulled | 50 | 14.45 | 65.95 | 2.89 | 39.28 | 3.52 | 7.08 |
| B4540 | 2004 | Hulled | 98 | 14.56 | 63.92 | 3.43 | 37.60 | 3.39 | 9.05 |
| B4403 | 2004 | Hulled | 90 | 13.43 | 67.88 | 2.58 | 43.21 | 3.66 | 7.93 |
| B3998 | 2004 | Hulled | 82 | 13.66 | 66.99 | 3.55 | 33.18 | 3.39 | 7.21 |
| B3748 | 2016 | Hulled | 92 | 16.82 | 49.44 | 9.58 | 30.21 | 3.14 | 13.40 |
| B9385 | 2016 | Hulled | 100 | 16.91 | 49.59 | 7.27 | 44.23 | 3.52 | 11.55 |
| B8389 | 2016 | Hulled | 96 | 16.07 | 51.60 | 8.03 | 36.76 | 3.43 | 12.48 |
| B9293 | 2016 | Hulled | 85 | 17.79 | 51.84 | 6.54 | 48.19 | 3.72 | 12.66 |
| B7580 | 2016 | Hulled | 82 | 14.13 | 52.30 | 9.12 | 48.21 | 3.59 | 12.92 |
| B17685 | 2016 | Hulled | 76 | 20.31 | 52.58 | 7.63 | 41.42 | 3.52 | 11.40 |
| B9038 | 2016 | Hulled | 94 | 16.86 | 52.62 | 5.86 | 35.93 | 3.24 | 10.57 |
| B9206 | 2016 | Hulled | 98 | 17.63 | 53.40 | 6.08 | 40.41 | 3.37 | 10.71 |
| B17414 | 2016 | Hulled | 98 | 15.22 | 53.93 | 7.66 | 41.57 | 3.48 | 12.13 |

| | | | | | | | | | |
|---|---|---|---|---|---|---|---|---|---|
| B8503 | 2016 | Hulled | 94 | 16.01 | 54.24 | 7.33 | 28.17 | 2.91 | 11.02 |
| B9441 | 2016 | Hulled | 100 | 16.70 | 54.30 | 6.76 | 52.30 | 3.82 | 12.32 |
| B9409 | 2016 | Hulled | 94 | 15.81 | 54.52 | 6.47 | 48.06 | 3.70 | 12.09 |
| B9146 | 2016 | Hulled | 98 | 15.70 | 54.65 | 5.68 | 50.06 | 3.66 | 10.71 |
| B9085 | 2016 | Hulled | 94 | 15.06 | 54.82 | 6.15 | 54.43 | 3.67 | 11.96 |
| B9317 | 2016 | Hulled | 86 | 15.98 | 54.92 | 6.45 | 42.16 | 3.56 | 11.21 |
| B3342 | 2016 | Hulled | 98 | 14.68 | 55.12 | 5.58 | 45.60 | 3.78 | 9.80 |
| B7088 | 2016 | Hulled | 30 | 14.64 | 55.17 | 7.06 | 38.58 | 3.40 | 12.16 |
| B7133 | 2016 | Hulled | 66 | 15.76 | 55.30 | 6.18 | 43.84 | 3.54 | 10.49 |
| B9100 | 2016 | Hulled | 96 | 15.20 | 55.53 | 6.62 | 32.30 | 3.25 | 10.92 |
| B9187 | 2016 | Hulled | 96 | 14.96 | 55.64 | 5.53 | 52.53 | 3.77 | 10.30 |
| B3647 | 2016 | Hulled | 94 | 13.25 | 55.69 | 8.70 | 42.19 | 3.47 | 13.49 |
| B8965 | 2016 | Hulled | 98 | 14.82 | 55.75 | 5.28 | 45.27 | 3.52 | 9.82 |
| B9250 | 2016 | Hulled | 94 | 14.49 | 55.81 | 5.55 | 44.79 | 3.61 | 11.72 |
| B9164 | 2016 | Hulled | 90 | 15.68 | 55.84 | 5.64 | 36.34 | 3.28 | 10.97 |
| B5321 | 2016 | Hulled | 38 | 14.74 | 55.98 | 5.67 | 29.95 | 3.12 | 10.24 |
| B9024 | 2016 | Hulled | 94 | 15.45 | 56.00 | 6.00 | 40.91 | 3.48 | 10.08 |
| B5771 | 2016 | Hulled | 78 | 16.91 | 56.16 | 4.70 | 37.03 | 3.45 | 8.68 |
| B9122 | 2016 | Hulled | 66 | 14.74 | 56.19 | 6.45 | 34.06 | 3.29 | 10.31 |
| B18587 | 2016 | Hulled | 96 | 14.87 | 56.23 | 5.34 | 30.05 | 3.31 | 7.71 |
| B7105 | 2016 | Hulled | 62 | 14.21 | 56.44 | 7.62 | 38.01 | 3.32 | 12.76 |
| B7364 | 2016 | Hulled | 94 | 15.08 | 56.51 | 7.02 | 43.64 | 3.40 | 12.15 |
| B7119 | 2016 | Hulled | 98 | 15.93 | 56.53 | 5.96 | 35.36 | 3.43 | 10.33 |
| B9009 | 2016 | Hulled | 100 | 13.32 | 56.76 | 5.64 | 52.07 | 3.65 | 12.01 |
| B20476 | 2016 | Hulled | 100 | 16.98 | 57.14 | 6.31 | 33.47 | 3.65 | 8.72 |
| B4971 | 2016 | Hulled | 74 | 14.17 | 57.70 | 5.95 | 48.06 | 3.77 | 11.35 |
| B17768 | 2016 | Hulled | 94 | 16.67 | 57.97 | 6.94 | 37.72 | 3.34 | 11.63 |

| | | | | | | | | | |
|---|---|---|---|---|---|---|---|---|---|
| B8994 | 2016 | Hulled | 98 | 13.90 | 57.98 | 5.05 | 42.01 | 3.49 | 10.65 |
| B8065 | 2016 | Hulled | 94 | 14.86 | 57.99 | 6.45 | 44.26 | 3.73 | 10.93 |
| B8980 | 2016 | Hulled | 100 | 14.06 | 58.12 | 4.55 | 42.32 | 3.52 | 10.31 |
| B9358 | 2016 | Hulled | 98 | 18.60 | 58.27 | 6.17 | 50.54 | 3.85 | 11.61 |
| B6178 | 2016 | Hulled | 92 | 12.65 | 58.37 | 5.71 | 38.92 | 3.56 | 10.18 |
| B17707 | 2016 | Hulled | 88 | 20.92 | 58.38 | 6.66 | 43.51 | 3.68 | 11.98 |
| B9229 | 2016 | Hulled | 98 | 12.79 | 58.42 | 5.82 | 35.68 | 3.21 | 12.04 |
| B17727 | 2016 | Hulled | 90 | 15.79 | 58.46 | 6.15 | 44.21 | 3.57 | 11.65 |
| B18393 | 2016 | Hulled | 100 | 15.34 | 59.08 | 5.32 | 37.22 | 3.47 | 8.87 |
| B6067 | 2016 | Hulled | 94 | 15.55 | 59.22 | 4.53 | 37.82 | 3.58 | 8.70 |
| B7818 | 2016 | Hulled | 92 | 15.05 | 59.67 | 4.91 | 38.60 | 3.43 | 9.29 |
| B4441 | 2016 | Hulled | 10 | 13.09 | 60.10 | 6.28 | 45.52 | 3.56 | 11.13 |
| B7229 | 2016 | Hulled | 94 | 14.68 | 60.19 | 4.24 | 48.76 | 3.83 | 9.70 |
| B19632 | 2016 | Hulled | 92 | 12.76 | 60.25 | 4.81 | 41.48 | 3.45 | 11.04 |
| B24234 | 2016 | Hulled | 92 | 16.54 | 60.34 | 4.45 | 37.76 | 3.54 | 8.19 |
| B9052 | 2016 | Hulled | 92 | 14.65 | 61.92 | 5.56 | 54.81 | 3.92 | 11.11 |
| B20943 | 2016 | Hulled | 96 | 14.03 | 62.45 | 3.48 | 51.11 | 3.88 | 9.36 |
| B20957 | 2016 | Hulled | 100 | 12.17 | 62.54 | 4.09 | 48.74 | 3.88 | 9.49 |
| B20929 | 2016 | Hulled | 84 | 13.44 | 63.70 | 3.70 | 45.95 | 3.83 | 8.78 |
| B20906 | 2016 | Hulled | 94 | 12.17 | 64.95 | 4.09 | 45.93 | 3.91 | 9.72 |
| B5005 | 2017 | Hulled | 98 | 11.49 | 63.43 | 3.87 | 29.43 | 3.24 | 8.85 |
| B19012 | 2017 | Hulled | 96 | 11.69 | 62.67 | 4.62 | 36.70 | 3.56 | 8.96 |
| B20556 | 2017 | Hulled | 98 | 12.17 | 64.11 | 3.36 | 44.81 | 3.71 | 8.52 |
| B20216 | 2017 | Hulled | 90 | 12.42 | 57.76 | 6.00 | 46.42 | 3.50 | 13.43 |
| B20975 | 2017 | Hulled | 88 | 12.79 | 66.14 | 3.25 | 49.58 | 3.96 | 9.57 |
| B20156 | 2017 | Hulled | 94 | 12.81 | 58.96 | 5.21 | 45.50 | 3.62 | 12.30 |
| B19884 | 2017 | Hulled | 94 | 12.83 | 55.25 | 7.07 | 41.16 | 3.39 | 13.40 |

| | | | | | | | | | |
|---|---|---|---|---|---|---|---|---|---|
| B19036 | 2017 | Hulled | 98 | 12.92 | 62.81 | 3.85 | 43.35 | 3.56 | 10.08 |
| B20167 | 2017 | Hulled | 100 | 12.95 | 58.89 | 6.58 | 37.98 | 3.46 | 12.13 |
| B18964 | 2017 | Hulled | 96 | 13.03 | 62.98 | 3.96 | 43.05 | 3.56 | 10.08 |
| B19020 | 2017 | Hulled | 92 | 13.06 | 61.24 | 3.63 | 55.44 | 3.86 | 10.55 |
| B19004 | 2017 | Hulled | 90 | 13.09 | 65.65 | 3.35 | 40.21 | 3.52 | 9.43 |
| B20039 | 2017 | Hulled | 88 | 13.19 | 61.16 | 4.88 | 45.46 | 3.74 | 10.10 |
| B19871 | 2017 | Hulled | 94 | 13.22 | 64.52 | 3.75 | 46.23 | 3.73 | 10.02 |
| B20267 | 2017 | Hulled | 92 | 13.35 | 59.33 | 5.32 | 36.28 | 3.30 | 11.55 |
| B20259 | 2017 | Hulled | 80 | 13.42 | 61.01 | 4.93 | 42.53 | 3.42 | 12.41 |
| B3334 | 2017 | Hulled | 100 | 13.47 | 59.17 | 3.63 | 57.83 | 3.96 | 11.17 |
| B3835 | 2017 | Hulled | 44 | 13.53 | 59.83 | 4.58 | 41.90 | 3.54 | 11.68 |
| B18988 | 2017 | Hulled | 100 | 13.57 | 60.75 | 4.07 | 47.60 | 3.75 | 10.00 |
| B18980 | 2017 | Hulled | 86 | 13.65 | 62.71 | 3.96 | 49.30 | 3.80 | 9.99 |
| B20175 | 2017 | Hulled | 24 | 13.65 | 61.53 | 5.17 | 31.49 | 3.30 | 10.77 |
| B20202 | 2017 | Hulled | 44 | 13.69 | 63.01 | 4.81 | 44.92 | 3.50 | 12.08 |
| B18940 | 2017 | Hulled | 62 | 13.71 | 62.41 | 3.74 | 48.92 | 3.71 | 10.68 |
| B19044 | 2017 | Hulled | 100 | 13.75 | 62.40 | 3.75 | 41.39 | 3.59 | 8.97 |
| B19028 | 2017 | Hulled | 88 | 13.75 | 60.21 | 3.65 | 50.84 | 3.72 | 10.47 |
| B18972 | 2017 | Hulled | 88 | 13.76 | 62.57 | 3.86 | 46.27 | 3.74 | 9.78 |
| B18996 | 2017 | Hulled | 94 | 13.78 | 60.96 | 3.76 | 48.77 | 3.79 | 9.91 |
| B7673 | 2017 | Hulled | 62 | 13.86 | 58.21 | 4.59 | 33.92 | 3.31 | 10.61 |
| B20130 | 2017 | Hulled | 94 | 13.89 | 58.65 | 5.60 | 41.99 | 3.48 | 12.03 |
| B18948 | 2017 | Hulled | 98 | 13.99 | 62.11 | 3.44 | 52.74 | 3.86 | 9.79 |
| B20461 | 2017 | Hulled | 100 | 14.05 | 58.77 | 4.21 | 39.56 | 3.48 | 9.51 |
| B4052 | 2017 | Hulled | 94 | 14.14 | 61.78 | 3.25 | 47.58 | 3.70 | 9.80 |
| B20138 | 2017 | Hulled | 72 | 14.21 | 57.36 | 6.44 | 46.79 | 3.69 | 12.14 |
| B20107 | 2017 | Hulled | 100 | 14.39 | 64.60 | 3.05 | 48.81 | 3.80 | 10.12 |

| | | | | | | | | | |
|---|---|---|---|---|---|---|---|---|---|
| B20079 | 2017 | Hulled | 100 | 14.57 | 65.05 | 3.06 | 41.00 | 3.42 | 10.01 |
| B20048 | 2017 | Hulled | 94 | 14.70 | 59.32 | 5.28 | 45.72 | 3.67 | 11.72 |
| B18864 | 2017 | Hulled | 84 | 14.71 | 57.20 | 5.31 | 35.03 | 3.38 | 11.33 |
| B5243 | 2017 | Hulled | 86 | 14.71 | 53.22 | 6.74 | 54.22 | 4.04 | 12.62 |
| B19052 | 2017 | Hulled | 98 | 14.78 | 62.02 | 3.75 | 51.40 | 3.93 | 9.68 |
| B20314 | 2017 | Hulled | 96 | 14.83 | 53.96 | 7.11 | 59.11 | 3.70 | 13.21 |
| B20234 | 2017 | Hulled | 92 | 14.88 | 53.72 | 7.14 | 34.84 | 3.30 | 10.07 |
| B20408 | 2017 | Hulled | 62 | 14.96 | 57.89 | 5.37 | 36.61 | 3.65 | 9.30 |
| B20185 | 2017 | Hulled | 82 | 14.97 | 57.95 | 4.82 | 48.47 | 3.74 | 12.01 |
| B19060 | 2017 | Hulled | 94 | 14.99 | 60.50 | 3.41 | 49.95 | 3.83 | 9.48 |
| B20296 | 2017 | Hulled | 90 | 15.12 | 59.44 | 4.07 | 34.19 | 3.28 | 9.55 |
| B20288 | 2017 | Hulled | 68 | 15.17 | 56.14 | 5.99 | 27.92 | 3.16 | 9.07 |
| B19535 | 2017 | Hulled | 60 | 15.25 | 57.93 | 4.71 | 38.56 | 3.46 | 10.50 |
| B8264 | 2017 | Hulled | 100 | 15.29 | 55.25 | 5.40 | 35.99 | 3.53 | 12.19 |
| B17604 | 2017 | Hulled | 44 | 15.48 | 55.42 | 6.09 | 43.49 | 3.58 | 12.93 |
| B5919 | 2017 | Hulled | 98 | 15.49 | 58.00 | 5.41 | 37.16 | 3.30 | 10.89 |
| B20397 | 2017 | Hulled | 94 | 15.52 | 56.01 | 5.04 | 43.19 | 3.37 | 12.14 |
| B20278 | 2017 | Hulled | 100 | 16.10 | 57.74 | 3.95 | 32.26 | 3.21 | 9.01 |
| B18956 | 2017 | Hulled | 82 | 16.46 | 58.75 | 4.06 | 50.18 | 3.81 | 9.90 |
| B20540 | 2017 | Hulled | 76 | 16.79 | 66.32 | 2.57 | 35.52 | 3.21 | 8.87 |
| B3754 | 2017 | Hulled | 66 | 17.80 | 59.16 | 2.88 | 37.79 | 3.66 | 10.35 |
| B4324 | 2017 | Hulled | 76 | 18.02 | 55.06 | 5.36 | 44.43 | 3.92 | 9.35 |
| B20372 | 2017 | Hulled | 90 | 18.28 | 54.21 | 5.13 | 29.39 | 3.00 | 11.37 |
| B20548 | 2017 | Hulled | 90 | 22.04 | 65.70 | 1.51 | 30.28 | 3.22 | 7.39 |

**Supplementary Table S3. Summary statistics of correlation analysis of barley seed viability with grain protein, starch, and NDF content, seed weight, width, and length.**

| Harvest Year | Grain Protein Content | | Grain Starch Content | | Grain NDF Content | | Thousand Grain Weight | | Grain Width | | Grain Length | |
|---|---|---|---|---|---|---|---|---|---|---|---|---|
| | r | Pv | r | Pv | r | Pv | r | Pv | r | Pv | r | Pv |
| **1975** | -0.687 | *** | -0.339 | 0.006 | 0.589 | *** | 0.03 | 0.812 | 0.011 | 0.93 | 0.409 | *** |
| **1976** | -0.157 | 0.002 | -0.41 | *** | 0.369 | *** | -0.025 | 0.626 | -0.43 | *** | 0.294 | *** |
| **1979** | -0.334 | *** | 0.044 | 0.63372 | -0.267 | 0.003 | 0.048 | 0.600149 | 0.036 | 0.692112 | -0.436 | *** |
| **2003** | -0.412 | 0.001 | 0.127 | 0.344 | 0.037 | 0.783 | 0.29 | -0.028 | 0.283 | -0.032 | 0.032 | 0.808 |
| **2004** | -0.222 | *** | -0.051 | 0.39 | 0.043 | 0.467 | -0.187 | 0.001 | -0.16 | 0.006 | 0.085 | 0.153 |
| **2016** | 0.101 | 0.458 | -0.014 | 0.916 | -0.145 | 0.283 | 0.138 | 0.309 | 0.191 | 0.157 | -0.114 | 0.402 |
| **2017** | 0.054 | 0.684 | -0.164 | 0.218 | 0.125 | 0.346 | -0.083 | 0.533 | -0.058 | 0.662 | -0.035 | 0.791 |
